# Supplementary material for: Network of large pedigrees reveals social practices of Avar communities
Source: Nature. 2024 Apr 24;629(8011):376–83. doi: 10.1038/s41586-024-07312-4 (PMC11078744; doi:10.1038/s41586-024-07312-4)
Supplement: Supplementary file 1 — This file contains Supplementary text and data, including Supplementary Figs. 1–50, Supplementary Tables 7–11 and Supplementary references. [file 41586_2024_7312_MOESM1_ESM.pdf]

nature portfolio

https://doi.org/10.1038/s41586-024-07312-4

Supplementary information

Network of large pedigrees reveals social practices of Avar communities

In the format provided by the
authors and unedited

Supplementary Information

Network of large pedigrees reveals social practices of Avar communities

Guido Alberto Gnecchi-Ruscone*[,#,1], Zsófia Rácz*[2], Levente Samu[2], Tamás Szeniczey[3], Norbert Faragó[2], Corina Knipper[4], Ronny Friedrich[4], Denisa Zlámalová[5], Luca Traverso[1], Salvatore Liccardo[6,7], Sandra Wabnitz[6,7], Divyaratan Popli[8], Ke Wang[1,9], Rita Radzeviciute[1], Bence Gulyás[10], István Koncz[2], Csilla Balogh[11], Gabriella M. Lezsák[12], Viktor Mácsai[2], Magdalena M. E. Bunbury[13], Olga Spekker[2,14], Petrus le Roux[15], Anna Szécsényi-Nagy[16], Balázs Gusztáv Mende[16], Heidi Colleran[17,18], Tamás Hajdu[3], Patrick Geary[19], Walter Pohl[6,7], Tivadar Vida[#,2,20], Johannes Krause[#,1] & Zuzana Hofmanová[#,1,5]

*co-first

[#]corresponding authors

[1]Department of Archaeogenetics, Max Planck Institute for Evolutionary Anthropology, 04103, Leipzig, Germany
[2]Institute of Archaeological Sciences, ELTE - Eötvös Loránd University, 1088, Budapest, Hungary
[3]Department of Biological Anthropology, ELTE - Eötvös Loránd University, 1117, Budapest, Hungary
[4]Curt Engelhorn Center for Archaeometry gGmbH, 68159 Mannheim, Germany
[5]Department of Archaeology and Museology, Faculty of Arts, Masaryk University, 60200, Brno, Czechia
[6]Department of History, University of Vienna, Universitätsring 1, 1010 Wien
[7]Institute for Medieval Research, Austrian Academy of Sciences, Ignaz-Seipel-Platz 2, 1010 Wien
[8]Department of Genetics, Max Planck Institute for Evolutionary Anthropology, 04103, Leipzig, Germany
[9]MOE Key Laboratory of Contemporary Anthropology, Department of Anthropology and Human Genetics, School of Life Sciences, Fudan University, 200438, Shanghai, China
[10]Hungarian National Museum,1088, Budapest, Hungary
[11]Department of Art History, Istanbul Medeniyet University, 34720, Istanbul, Türkiye
[12]Institute of History, HUN-REN Research Centre for the Humanities, 1097, Budapest, Hungary
[13]ARC Centre of Excellence for Australian Biodiversity and Heritage, College of Arts, Society and Education, James Cook University, Cairns QLD 4870, Australia
[14]Department of Biological Anthropology, University of Szeged, H-6726 Szeged, Hungary
[15]Department of Geological Sciences, University of Cape Town, 7700, Rondebosch, South Africa
[16]Institute of Archaeogenomics, HUN-REN Research Centre for the Humanities, 1097, Budapest, Hungary
[17]BirthRites Lise Meitner Research Group, Max Planck Institute for Evolutionary Anthropology, Leipzig, Germany
[18]Department of Human Behavior, Ecology and Culture, Max Planck Institute for Evolutionary Anthropology
[19]Institute for Advanced Study, Princeton, NJ 08540, USA
[20]Institute of Archaeology, HUN-REN Research Centre for the Humanities, 1097, Budapest, Hungary

Supplementary Information 0
Historical evidence for the population history of the eastern Carpathian Basin (c. 300-800 CE) 2
The role of ethnic groups in the population history of the period 2
Population shifts in the eastern Carpathian Basin, c. 300-800 CE 3
Archaeological and anthropological description of the Avar-period cemeteries of Rákóczifalva, Hajdúnánás, Kunpeszér, and Kunszállás (Hungary) 5
Rákóczifalva-Bagi-földek Site 8 and 8A (Jász-Nagykun-Szolnok county, Hungary) 6
Anthropology 6
Archaeology 7
The Rákóczifalva cemetery, the Transtisza region and the Pontic Steppe in the 6th-7th century 9
Byzantine connections 10
Settlements in Rákóczifalva 11
Sarmatian-, Late-Sarmatian-Hunnic- and Gepidic-period graves 12
Hajdúnánás-Fürj-halom-járás Site 41A (Hajdú-Bihar county, Hungary) 13
Anthropology 14
Archaeology 14
Kunpeszér, Felsőpeszéri út (Bács-Kiskun county, Hungary) 14
Anthropology 15
Archaeology 15
Kunszállás, Fülöpjakab (Bács-Kiskun county, Hungary) 17
Anthropology 17
Archaeology 17
Spatial distribution of pedigrees and unrelated individuals in the Rákóczifalva cemetery 18
Pedigrees, related and unrelated individuals and status 20
Reproductive partners 24
Social organization in the Avar Khaganate and its parallels in historical steppe societies 25
Population shifts and the middle Avar-period transformation 28
Migration history and admixture processes of the DTI and TT the Avar period population: evidence from population genomics, archaeology and history 29
Possible historical contexts for pre-Avar-period admixture 37
European ancestry individuals and the smaller pedigrees of RK 38
Pedigree building strategy 40
Special cases in pedigree building process 41
Genetic relatedness validation with ancIBD, KIN and PMR 45
Strontium isotope data of the burials of Kunpeszér, Kunszállás and Rákóczifalva 51
Carbon and nitrogen isotope data 55
Dataset and collagen quality 55
General characteristics of the human diet 56
Internal differentiation 58
Age 59
Chronology 60
Sex and social differentiation 63
Detailed 14C results and bayesian modeling 70
IBD network statistics 80
References 87

Historical evidence for the population history of the eastern Carpathian Basin (c. 300-800 CE)

By Walter Pohl

The role of ethnic groups in the population history of the period

Although there is no written source from the Carpathian Basin that describes the history of the period before, during and after the Avar period, we have relatively good information on the population movements and the succession of political formations. This provides a historical context for the genetic and archaeological results of this study. Many of the texts come from Eastern Rome, also called Byzantium in modern scholarship, and its capital Constantinople (modern Istanbul), others were written in the Frankish kingdom (which roughly comprised modern France and the South and West of Germany) and in Italy[1]. These texts use ethnic labels to describe population groups and political units, which is their chief way to describe collective agency. Modern scholars can hardly avoid following this terminology. This is basically adequate because it reflects contemporaneous perceptions; but we have to distinguish. In some cases, it probably largely corresponds to the self-identification of the people labeled that way; in others, it describes political allegiances rather than ethnic identities; and yet in others, the names in the texts are only erudite ethnographic descriptions. Historical source critique can often provide criteria to decide between these options[2].

However, we have to be careful because the way in which these terms are used is generally fuzzy; 'Avars', for instance, can sometimes mean an elite or core group, sometimes a broader population, and sometimes designate the entire population of the realm. Over time, these uses generally shift from a narrower to a wider conception of the term[1]. It is difficult to correlate the name taken from the sources with a cultural group emerging from the archaeological record, because we can only guess which artifacts or practices may have been regarded as signs of identity, if at all[3]. The self-identification of the individuals whose genome we have analysed remains elusive, and we certainly cannot take for granted that the ethnic names correspond to genetic ancestries[4].

The group of Avars that arrived north of the Caucasus in 557 CE may be a rare case in which their designation in the texts and their mostly Eastern Asian ancestry seem to have largely overlapped[5]. In the course of the processes of admixture and the social dynamics described in our research, this correspondence was most probably blurred. By the 8th century CE, the written sources subsumed the entire population of the Avar realm under the name 'Avars'[6]. The uniformity of status representation and of cultural expressions of this period shows that these people did not seek to emphasize distinctions between them. We cannot exclude that under the umbrella of a common cultural identity and shared allegiance to the Avar polity, some individuals or groups adhered to other ethnic identities. However, there is no indication in the historical and archaeological sources that Avar identity could have been limited to a predominantly Eastern Asian ancestry, which is present in different individuals to a rather different extent and would have been hard to delineate.

Population shifts in the eastern Carpathian Basin, c. 300-800 CE

The DTI and TT regions, east of the Danube, never were part of the Roman Empire, which had conquered the western part of the Carpathian Basin west of the north-south tract of the Danube, around the turn of the era, and established the province of Pannonia which remained under Roman control for almost half a millennium. In the course of the 1st century CE, a large group of Sarmatians occupied the eastern half of the Carpathian Basin. The Sarmatians, who spoke an Iranian language, had largely replaced the Scythians in the steppes north of the Caucasus and the Black Sea in the 3rd/2nd centuries BCE. In the Carpathian Basin, they remained a regional power, and often operated in close conjunction with the Germanic Quadi who lived in what is now southern Slovakia. Roman observers noticed that the two peoples had come to resemble each other to a considerable degree, although the archaeological evidence only confirms this in the contact zone[7–9]. In the course of the 4th century, the Sarmatians had to face the threat from Goths and Gepids from the east and north-east, and from Vandals who expanded south from their homelands north of the Carpathian Mountains.

The situation in the Carpathian Basin changed in the aftermath of the arrival of the Huns, who came from somewhere in Central Asia to Europe in 375 CE. Goths, Alans and other groups moved west, and some of them settled in the Carpathian Basin. In c. 400, the Huns occupied the Carpathian Basin[10]. Most Vandals and Alans and some Gothic groups left and marched as far as Italy, Spain and North Africa. Many Sarmatians stayed in the TT and also DTI regions, and Ostrogoths, Gepids and others also remained under Hun rule. In the 430s, the short but dramatic story of the Hunnic Empire began, which reached its apogee under their legendary king Attila, who first devastated large parts of the Roman provinces in the Balkan Peninsula, and then organised two large military expeditions into Gaul (451) and Italy (452), at the head of an army which included several, mostly Germanic-speaking peoples[11]. We have a lively report from a Byzantine diplomat who visited Attila's wooden palace somewhere between Danube and Tisza, a rich and bustling cosmopolitan power centre.

Attila died in 453, and his empire collapsed in a series of conflicts between his sons and the subject peoples. The Hun realm dissolved into a number of smaller kingdoms, of which the Gepid kingdom in the TT region was the most durable[12]. The Goths dominated Pannonia until they left for a more profitable future in the Roman Empire. In the southern DTI, a small kingdom of the Sarmatians emerged, and further north, one of the Sciri; north of the Middle Danube, kingdoms of the Suebi and the Heruls existed for a generation or two. Other groups, often mixed between Huns and other groups, joined Roman service in the Balkan provinces immediately after the fall of Attila's kingdom. Some of Attila's sons with their following tried to carve out their own realms, but mostly retreated to the steppes north of the Lower Danube and the Black Sea. In the 6th century, Longobards moved into Pannonia, where they began to challenge the Gepids, their eastern neighbours. In 567, they destroyed the Gepid kingdom. In the following year, they united a large following of many groups still living in the

Carpathian Basin (reputedly Gepids, Bulgars, Suebi, Sarmatians and Pannonian provincials) and invaded Italy, where they founded their kingdom.

This is when the Avars came to the Carpathian Basin[1]. They had arrived north of the Caucasus in 557, and immediately sent an embassy to Constantinople to offer an alliance to the Byzantine emperor. 'Avars' was probably the current name in the Central Eurasian steppes for one of the peoples of the Rouran Empire in Chinese sources. They ruled a powerful empire with its centre in what is now Mongolia, until the Turks defeated them in 552[13]. In the following years, the Rouran disintegrated and were decimated by Chinese and Turkic attacks, inner conflicts and massacres. However, one large group must have escaped from the turmoil in the collapsing empire under the leadership of a khagan, which was the title of the supreme Rouran ruler, and went west. One of the Byzantine sources, probably based on information from the Turks, called them Varchonites and denied their Avar legacy, but that was a partisan view. Backed by the alliance with the Byzantines, the Avars systematically attacked and/or subdued the powers in the Eastern European steppe (Utigurs, Kutrigurs and others) and beyond (Antae, Slavs and even the Franks)[1].

When the Avars moved into the Carpathian Basin, large contingents of horsemen from the Pontic steppe must have accompanied them[1]. The texts mention 10,000 Kutrigurs, plus Utigurs and later mainly Bulgars (which might be an umbrella term including the two former groups). The Central Asian component was reinforced in the 580s by the arrival of three further groups of 'Varchonites'. We have relatively detailed accounts of the wars and negotiations between Avars and Byzantines in the last quarter of the 6th century, which also repeatedly attest to the presence of Gepids and Slavs in Avar armies. These ethnic units fighting for the khagan were clearly distinguishable, and after one Byzantine victory over the Avar army, we get numbers for the prisoners they took, which differ somehow between the two chronicles that report the event: 3000 Avars, 3200 Gepids, 8000 (or 800) Slavs and 3000 (or 2000) 'other barbarians', perhaps Bulgars (mentioned elsewhere in Avar armies). The numbers may be exaggerated, but the proportions are not unlikely[1]. The battle took place in 599 at the river Tisza during the only offensive conducted by the Byzantines into the Avar core area. After their victory, the Byzantines also massacred the peaceful inhabitants of a large Gepid village nearby. In the first quarter of the 7th century the Avars were again on the offensive, which culminated in a siege of the Byzantine capital Constantinople in 626 in an alliance with the Persians, and with large numbers of Bulgars and low-status Slavs bearing the brunt of the attack.

The siege failed, and soon after a Bulgar contender for the throne of the khagan challenged the Avar candidate; the latter prevailed, and thousands of Bulgars fled to the Bavarians, where most of them were killed[1]. In the Pontic steppe, the initiative now passed to Bulgars. Khan Kuvrat established a short-lived steppe empire there in the 630s, which succumbed to the Khazars some decades later. One of his sons, Khan Asparukh, withdrew to the region around the Danube delta and built another Bulgar realm there

in the 680s. Part of the Bulgars also reputedly came to the Avar empire. Bulgar presence is also attested there in the later 7th century in a miracle collection from Thessalonica. It reports how the offspring of the Byzantine captives of the Avar raids at the beginning of the century, who had been settled in Pannonia, mixed with Avars and Bulgars; then, under Bulgar leadership, they forced their way out of the Avar realm and marched on Thessalonica.

This is the last we hear about other peoples under Avar rule. The written evidence about the 8th-century Avars mostly comes from Frankish and Italian works of history, and it only mentions Avars in the Carpathian Basin. These texts were well-informed and have detailed information about the Central Asian titles of rank of the different Avar dignitaries, so their perceptions make it probable that the population of the Avar realm could now rather indistinctly be subsumed under the name ‘Avars’. This would correspond to the relatively uniform expressions of status used in the period, especially the belt sets and their fittings. However, after Frankish armies had subdued the Avar khaganate in the years 791-96, these Avars disappeared rather quickly from the written sources. The last mention of Avar envoys to the Frankish court comes in 822. What exactly happened to the Avar-period population will be the topic of future research.

Archaeological and anthropological description of the Avar-period cemeteries of Rákóczifalva, Hajdúnánás, Kunpeszér, and Kunszállás (Hungary)

In our study, we processed four entire Avar-period cemeteries, two from the core area of the Danube-Tisza Interfluve (DTI) and two from the neighboring Transtisza (TT) region: Kunpeszér (KUP), Kunszállás (KFJ), Hajdúnánás (HNJ), and Rákóczifalva (RKF, RKC), respectively. The biggest burial place with 308 graves is Rákóczifalva consisting of 3 large groups of graves and 2 solitary burials. It was continually occupied from the late 6th to the first half of the 9th century CE, that is during the early, middle and late Avar periods. A small cemetery from Hajdúnánás was also analyzed, which sheds light on how the Avar-period population occupied the northern section of the TT region in the mid-7th century. Both of the cemeteries from the DTI lie close to burial places of the highest Avar elite (like the supposed khagan's grave in Kunbábony): the Kunpeszér site itself contains some richly furnished male and female burials with exquisite gold- and silver-decorated weapons, belts, and jewelry from the first half of the 7th century. Kunszállás was founded in the mid-7th century and belongs to the more uniform material culture of the middle and late Avar period, but the higher social status of certain individuals is represented also in this case by gold artifacts. Furthermore, archaeological features reveal cultural and social differences between, and even within, regions on both sides of the Tisza. An example of this is that, graves rich in precious metals, including gold belt sets, weapons covered with gold sheets, and gold and silver vessels can be observed in the 7th century primarily in the Danube-Tisza Interfluve

region while burials with horse and horse harness were typical of the Transtisza region and some sites in Transdanubia.

Rákóczifalva-Bagi-földek Site 8 and 8A (Jász-Nagykun-Szolnok county, Hungary)

The Rákóczifalva (RK) cemetery was excavated in 2005-2006 by the Institute of Archaeological Sciences of the Eötvös Loránd University (Katalin Sebők, Gábor Szabó, Katalin Kovács, and Gábor Váczi) during rescue excavations of the construction of an emergency flood reservoir. The archaeological material of Site 8 (RKC) and 8A (RKF) was assessed in two MA theses by Viktor Mácsai (2012)[14] and Magdalena M. Schmid (2015)[15], respectively; the anthropological analyses were carried out by Tamás Szeniczey, Tamás Hajdu, and Antónia Marcsik, newly published in the current article.
The site is a part of a large site-complex located by the Tisza, on the former high bank of the river. The Avar-period graves lie on lengthy elevations running from South-East to North-West and divided by former watercourses. (Supplementary Fig. 1) The burial place consists of 308 graves (279 sampled) and was used ca. from 570 to the first half of 9th century CE (Supplementary Table 2). The graves are grouped into three large clusters, two of them are close by (Site 8A) and one (Site 8) is located ca. 100m apart (Fig. 2-3). Site 8 cemetery is formed by a main cluster of burials and two scattered Avar graves. The western group of the cemetery (8A) was used during the late 6th and the 7th century, both the middle and eastern groups mainly in the 2nd half of the 7th and in the 8th century. Avar settlement remains were also unearthed on Site 1, 2, 4, 5, 8, 8/a, 10, 11 and 12, these might be the residence of the population buried in the cemetery[16,17].

Anthropology

By T. Szeniczey, T. Hajdu, A. Marcsik

The remains of 304 individuals from the Avar period could be identified from the anthropological findings. Adults (>20 years) made up 76% of the individuals (231), with nearly comparable numbers of males (122) and females (105). The age group distribution, however, was less balanced among the young adults (20-35 years). The proportion of females exceeded the males possibly as a result of greater maternal mortality. In the case of 4 adult individuals, it was not possible to estimate the sex, moreover another 8 individuals (6 female, 2 male) were classified as juvenile/young adults. The number of the subadults (61) is underrepresented as they only account for 20% percent of the individuals which seems considerably biased compared to the expected proportion in pre-Jennerian populations[18]. Among the subadults, the ratio of neonates is remarkably low (0.65%), however other infant age categories are better represented (1-6 years: 7.2%; 7-13 years: 6.9%; 14-19 years: 5.2%). Thus the under enumeration of children is mostly the consequence of the missing neonates.
In this skeletal material, we did not find signs of perimortem trauma. The identified bone fractures were rather likely consequences of accidents and identified mostly among males. Based on the fracture type,

the majority can be related to horse riding (e.g. clavicle fracture), stress fractures (spondylolysis, due to age, physical activity, even horse riding).

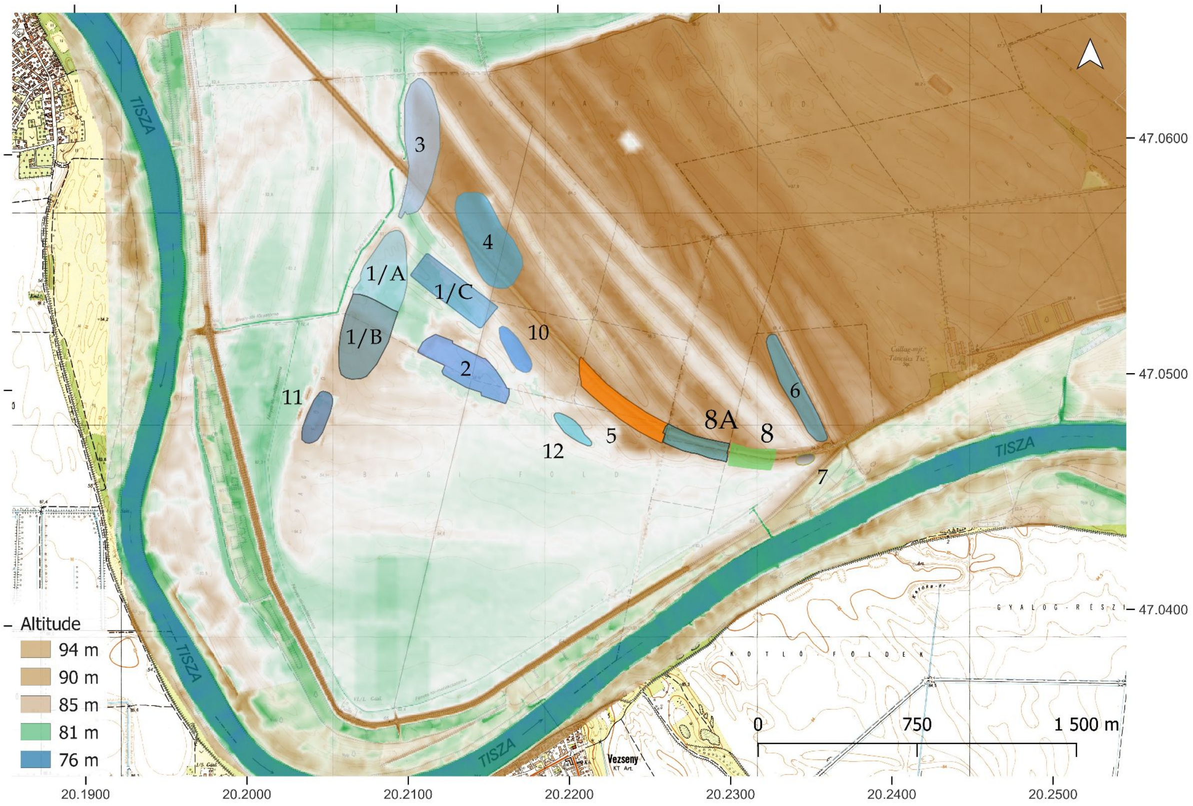

Supplementary Fig. 1 - Excavation sites around Rákóczifalva. Source of the basemap[104].

Archaeology

By V. Mácsai, Zs. Rácz, M. Bunbury

From an archaeological point of view, burial customs and archaeological material of the cemetery fit well the general picture of the Transtisza region in the Avar period. The orientation of the graves is north-south, a new influence from the East, as opposed to the west-east orientation that was common in Central Europe starting in the 4th-5th century. Applying complex grave forms like end-niche graves (4x), putting animal skin with the skull and legs (flayed animal/partial animal; 5x small ruminants, 2x cattles), whole horse (1x) (Supplementary Fig. 2), or horse harness in the grave (21x), were typical ritual phenomena in this region. Partial animal burials appear only in the early period, but large amounts of cattle bones (4x) appear in the latest graves as well. There is also an example (Site 8, G. 5) of two whole cattle skeletons placed over the deceased. Burying the dead with horse harness was particularly important in the RK community compared to other sites.

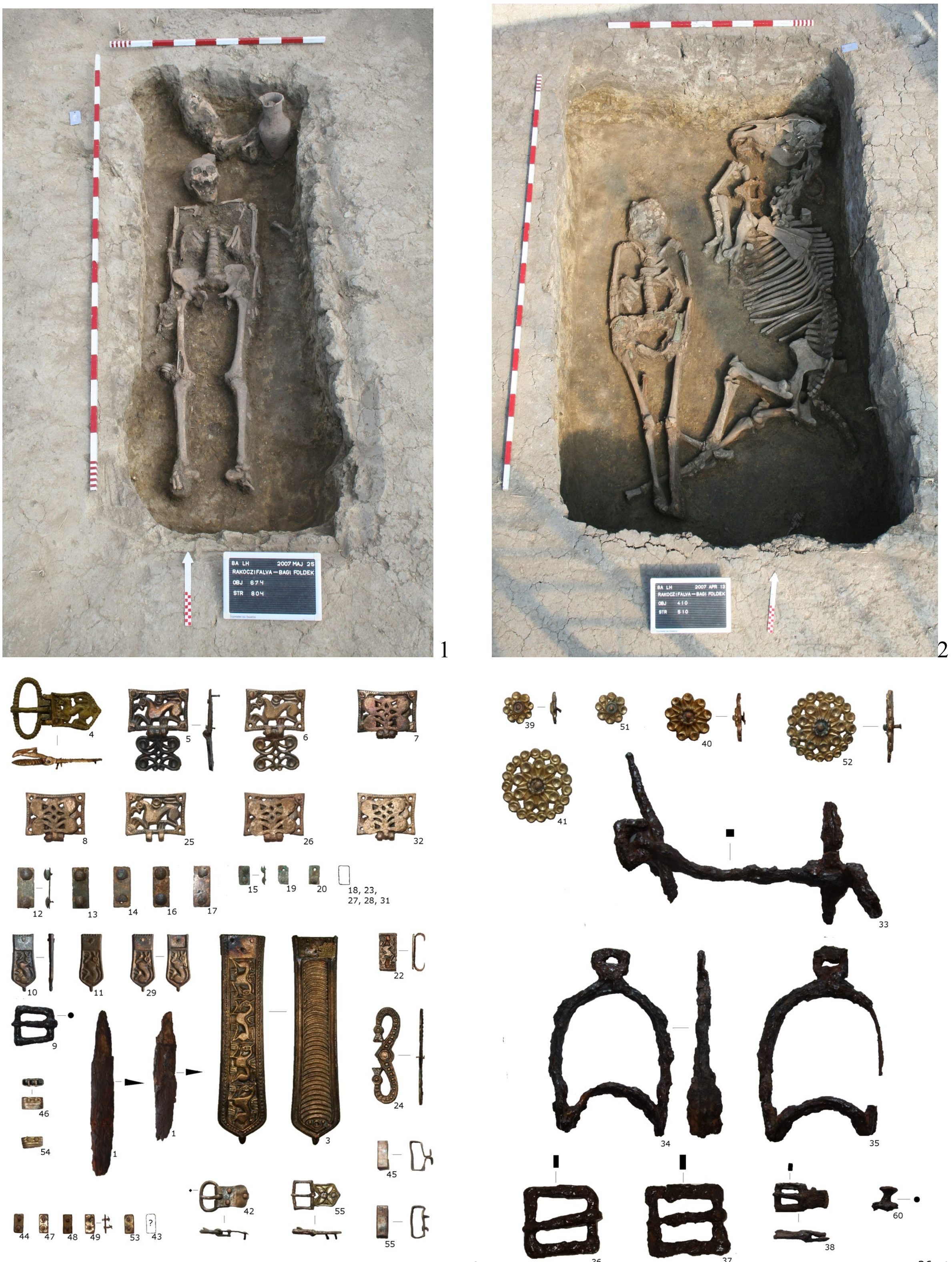

Supplementary Fig. 2 - 1: Early Avar-period female grave with pot and skull and extremities of a small ruminant (674/804, RKF103, G. 154); 2-4. Late Avar-period male grave with belt set, horse and horse harness (410/510, RKF012, G. 26)[105].

Ceramic vessels and animal bones serving as food offerings are relatively common as well in the earlier graves of RK (27x ceramic vessels, 16x animal food offerings in Site 8A, and 1x ceramic vessel and 5x animal bones in Site 8), placing hand-formed pots next to the head of the deceased was a typical phenomenon in the early Avar-period Transtisza region (Supplementary Fig. 2). Grave constructions with piles appeared in the later phases of the cemetery (32 cases in Site 8A, and 29 cases in Site 8), as well as throughout the Avar settlement area, from the middle of the seventh century.

The archaeological material consists of weapons (7 cases, bows, arrowheads and spear in the early and axes in the middle/late phase), belt sets, horse harness sometimes with fittings are found in richer male graves, while iron belt buckles and knives in average male graves and earrings, beads, and spindle whorls in female burials. Hand-formed vessels with funnel-shaped necks are a characteristic feature of the early Avar material culture east of the Tisza (TT region).

The Rákóczifalva cemetery, the Transtisza region and the Pontic Steppe in the 6th-7th century

By B. Gulyás

Several archaeological features of the TT sites are strikingly similar to 'nomadic' burials of the 6th-7th centuries in the Pontic-Caspian Steppe (mainly near the northern shores of the Black Sea, in the Crimean Peninsula, in the northern Caucasus foothills and along the lower and middle Don-Volga rivers) known as the Sivashovka-type burials. This group consists of cca. 100 burials situated in a vast territory between the Dniester River and the eastern foothills of the Ural Mountains (Supplementary Fig. 3), and is mainly defined by similar burial customs[19]. The N-S orientation, which is common in Rákóczifalva, was widespread among the nomadic population of the Hunnic and Post-Hunnic period, while it significantly decreased during the second half of the 6th and the 7th centuries. However, N-S oriented graves are known from the North Pontic Region and the Volga Basin as well. Cattle remains occurred rarely in the graves of the steppe region. Examples can be found in well known sites such as Politotdelskoe, Prepolovenka and Kamennyi Ambar-5, located in the Volga Basin and in the Eastern Urals. However, their position in the grave differs from the cases observed in the TT region, where the flayed skins stretched in the grave. In the graves from the Volga region, folded skin was placed at the feet of the deceased. Stretched cattle skin over the human remains was observed in Topyla (Ukraine), but the chronological position of this site is still debated[20]. The skulls and extremities of the small ruminants appeared in some graves in Rákóczifalva (Supplementary Fig. 2). This custom has excellent analogies among the Sivashovka-type burials. A dozen graves contain such remains, almost exclusively placed next to the feet or the skull of the deceased as it was documented in Rákóczifalva[21]. The burials with only horse harnesses are absent from the contemporary East European steppe, however, this rite was widespread in the Hunnic and Post Hunnic period, like in Ust'-Alma, Kyzyl-Adir, and Shipovo grave 3[19]. The vessels placed next to the skull are typical both in the TT region and in the Pontic-Caspian

steppe as well. The overwhelming majority of the pots are made without a potter's wheel, while the good quality jugs appear only in the Kuban region (Staronizhesteblievskaia, Kholmskoe)[19].

Based on this context, archaeological research suggests that part of the Avar-period TT population originated in this region that would be in agreement with the population genomic results discussed in the main text.

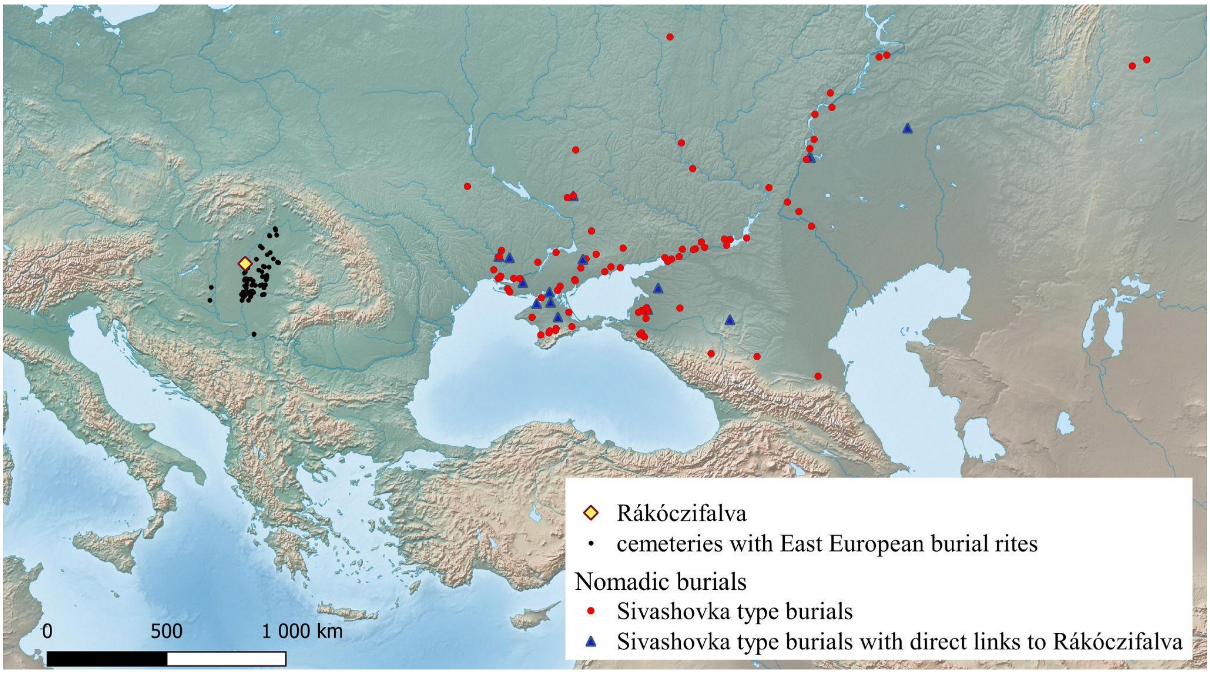

Supplementary Fig. 3 - Distribution of sites of 'Transtisza-type' in the Carpathian Basin and Sivashovka-type burials in the Pontic-Caspian steppe region. Base geographic map taken from[106].

Byzantine connections

By L. Samu

Due to the Avar Khaganate's ambitions of power and its extensive Byzantine connections, a significant amount of Mediterranean objects is found in early Avar graves[22]. The same picture can be traced in the case of the Rákóczifalva community. Original Byzantine/mediterranean artifacts (imports) and locally produced artifacts manufactured in mediterranean style or with special goldsmith techniques were also found in the graves in Rákóczifalva (Supplementary Fig. 4). These types of objects came to light from graves: 35-36, 68, 72, 89, 102, 107, 111, 115, 148, 163, 181, 198, 219, 208, 221, 233. An anthropomorphic gold figure – similar to some artifacts from the territory of the Balkan, north of the Black Sea, and the Caucasus – was also found on the surface of 8A, unfortunately without clear context[23]. These graves mostly belong to the early Avar period, some of them to the middle phase. Their spatial distribution is quite even among these early graves. Strikingly, their concentration was high only in the early 7th century. This picture fits very well to the general consideration, that the different Mediterranean objects are frequent mostly between the last third of the 6th and the middle of the 7th century[24]. It is important to emphasize that the Mediterranean influence is maintained in different ways

also in the second half of the Avar age, namely in the 8th c., which is reflected also in the belt sets and their decorations[25]. But the diversity of these objects in the earliest times was much higher, which can be also seen in the case of Rákóczifalva. This community was part of the exchange system in which also Mediterranean objects were circulating, during these exchanges they were able to reach prestigious potteries, belt elements, jewelleries and such peculiarities, as a Byzantine candleholder. The organic materials could tell us much more, but they were unfortunately unpreserved.

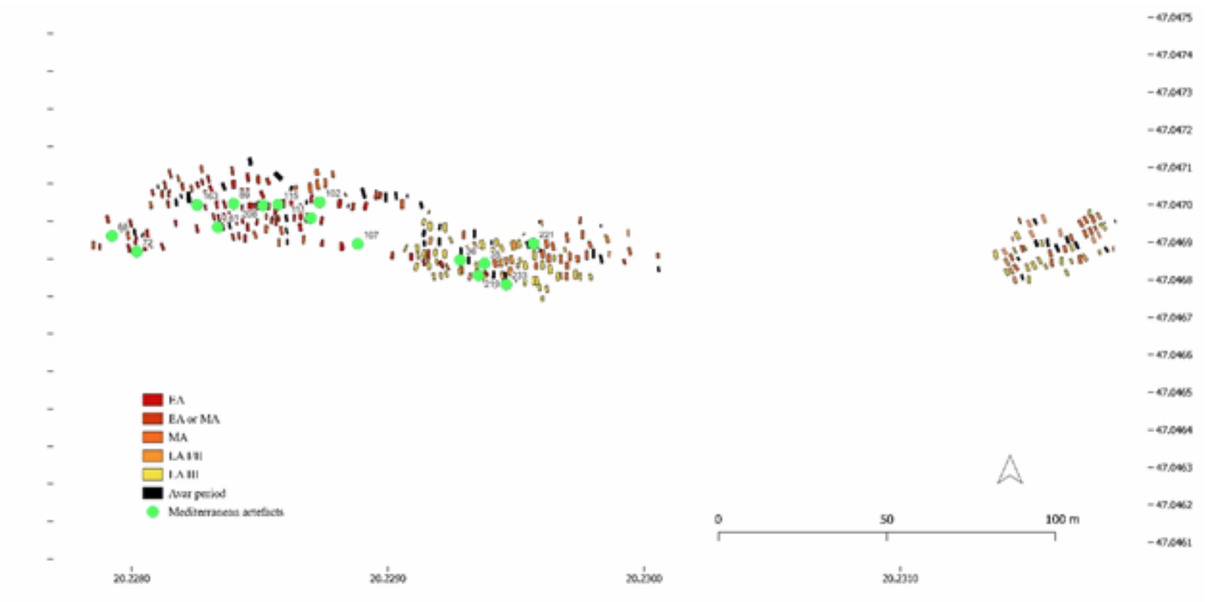

Supplementary Fig. 4 - Distribution of artifacts of mediterranean origin/style in the Rákóczifalva cemetery.

Settlements in Rákóczifalva

By T. Vida, L. Samu

The area of Rákóczifalva and its surroundings was a popular place of settlements and residence during the Migration Period and Early Middle Ages, and was home to populations of various origins. Successive or coexisting communities often settled in the same area for environmental and economic reasons. The above-mentioned SE-NW oriented longish elevations on the bank of the Tisza River (Supplementary Fig. 1) provided opportunities for communities to settle in areas that had excellent water, soil, and grass resources. The archaeological evidence of Sarmatian- and Gepidic-period communities follows each other closely chronologically, while the number of features showing an early settlement of Avars in the area is not high (end of 6th c.)[26]. The structure of the early Avar settlement is made up of groups of a few houses. A total of eight sites from Rákóczifalva have so far yielded Avar-period settlement phenomena (nos. 1/a, 1/c, 2, 4, 5, 8, 8/a, 10, 11, 12) and archaeological features and material from five of them was processed (1/a, 2, 5, 8, 8a)[16,17] (Supplementary Fig. 5). Among the houses, most of them were sunken floored buildings (no. 45), but there were also examples of post-

structure, above-ground buildings (no. 12) and a log cabin-like building/stilt house[16]. Most of the pottery was hand formed (ca. 95.5 %), only sporadic fragments of slowly turned pottery and wheel-turned gray, black, red and yellow pottery was found (4.5%). Some settlement features contained only hand-formed pottery. These typical types of vessels (pots, bottles, storage vessels, backing bells and clay cauldrons) are indicative of the lifestyle of a settled population. The archaeozoological and archaeobotanical material has not yet been processed. Though the ceramic vessels suggest a dating of the settlement features on the surfaces 2, 5, 8A and 8, between the late 6th and early 8th centuries, only the ^{14}C dating of the zoological material will give us a reliable chronological order of the different settlement parts. On the surface 1a we can date the settlement features - based on the ceramic typology - between the 7th and late 8th centuries[17].

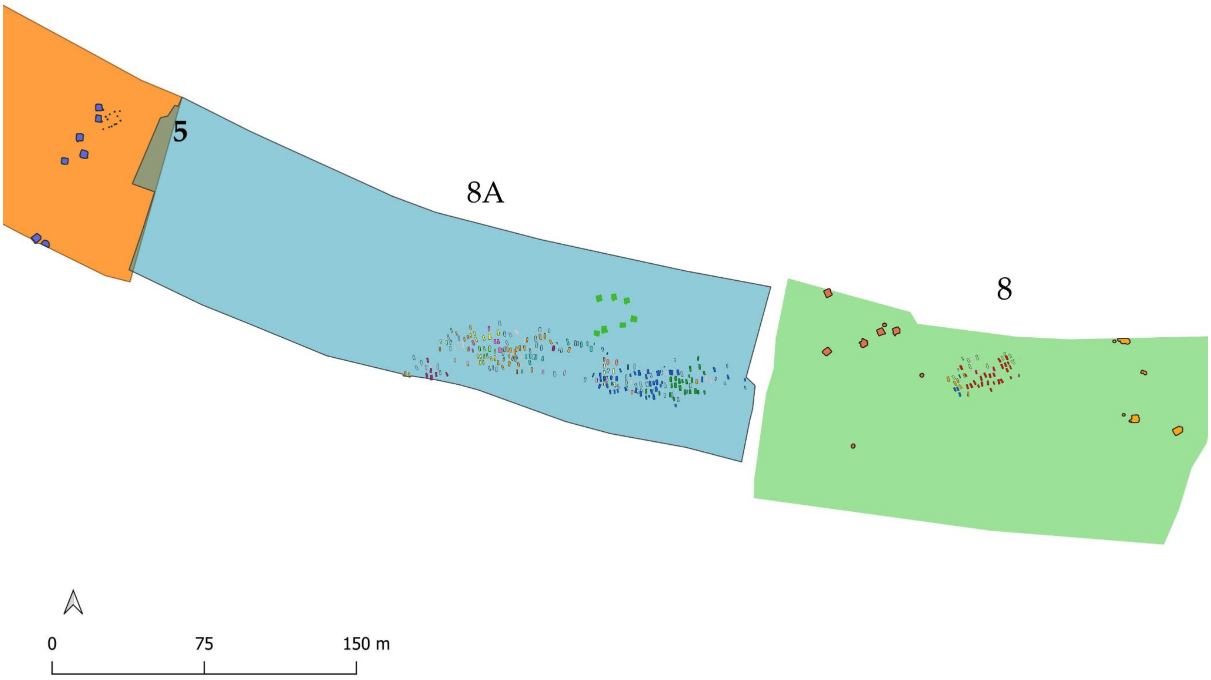

Supplementary Fig. 5 - Avar settlement features near the cemetery on Site 5, 8A, and 8.

Sarmatian-, Late-Sarmatian-Hunnic- and Gepidic-period graves

At the same site-complex of Rákóczifalva (Site 5, 8, and 8A), 56 graves of a Sarmatian cemetery from the 2nd-4th centuries, as well as several sporadic burials from the Sarmatian, Late Sarmatian-Hunnic (4th-5th century) and Gepidic periods (5th-6th century) connected to large settlements were also unearthed[27]. Some of the latter are solitary inhumation burials, others are burials put or thrown in settlement features (pits). In the present paper, these individuals were used as comparative material for the genetic analysis of the Avar-period population. Among the pre-Avar samples we found 20 closely related individuals that formed 5 different pedigrees (Supplementary Fig. 6). Four of these pedigrees

were composed of individuals from the main Sarmatian cemetery and only a pair of first degree individuals were detected among the scattered Gepidic-period burials.

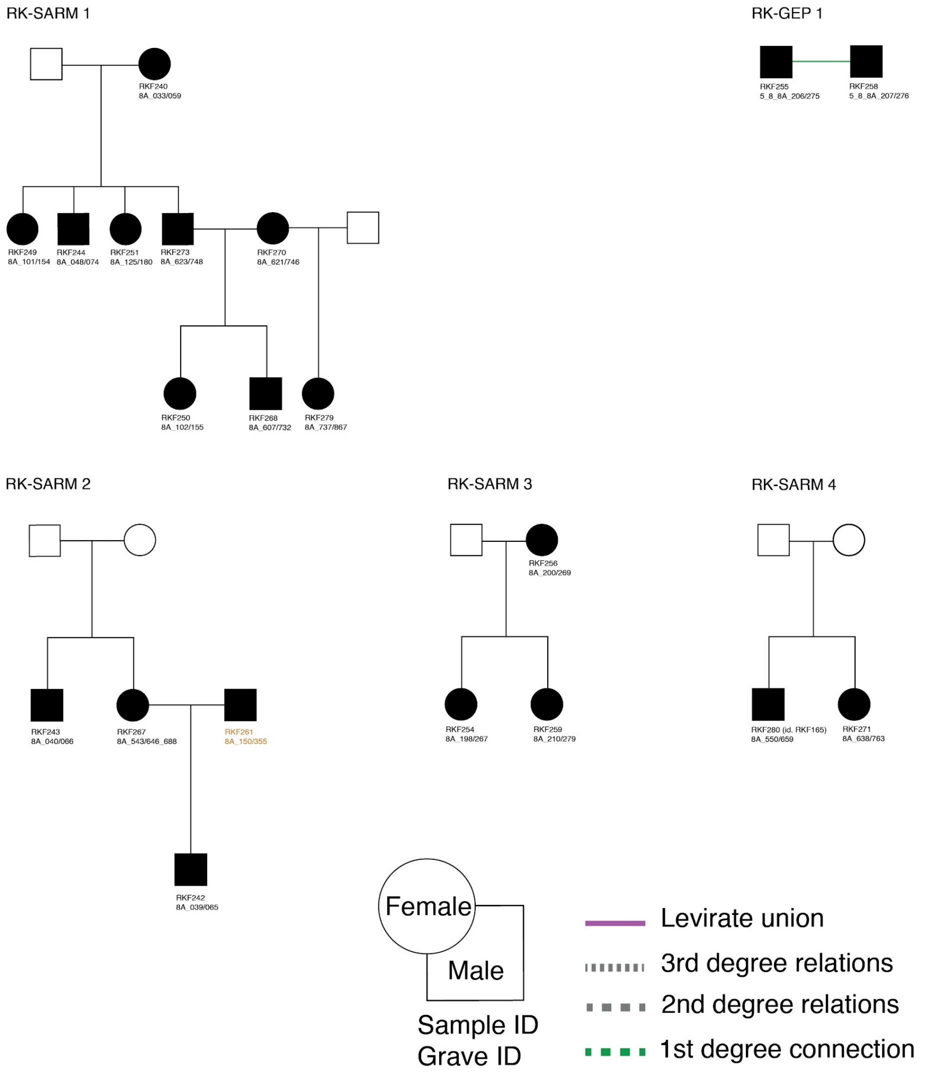

Supplementary Fig. 6 - Sarmatian and Gepidic-period pedigrees in RK site.

Hajdúnánás-Fürj-halom-járás Site 41A (Hajdú-Bihar county, Hungary)

The Hajdúnánás-Fürj-halom-járás site (M3 motorway Site 41A) was a rescue excavation in 2005 led by Gábor Szabó from the Institute of Archaeological Sciences of the Eötvös Loránd University. The Avar cemetery was archaeologically evaluated by Zsófia Rácz and Gergely Szenthe,[28,29] anthropological analysis was carried out by Tamás Hajdu, Zsuzsanna Guba, and Ildikó Pap[28,29]. Radiocarbon and Bayesian analyses based on Byzantine coin finds was elaborated by Zsuzsanna Siklósi[30]. The site is in the Transtisza region, ca. 25 km East of the Tisza River. Besides Sarmatian- and Hunnic-period archaeological features the site yielded 18 inhumation graves - a whole small cemetery

- from the 2nd half of the 7th century (Extended Data Fig. 3). All 18 individuals were successfully sampled for genetic analysis.

Anthropology

By T. Hajdu

Anthropological analysis revealed the remains of 18 individuals from the Avar period. Based on the estimation of age, the number of adults among the skeletons was 13 (4 males, 9 females). The sex ratio appears imbalanced, but based on the small sample size, no definite conclusion can be made. Out of 5 subadults, 4 were in the adolescent stage, covering early, middle, and late stages of development. The youngest was 4-5 years old. The founder individual of pedigree 1, a 35-45-year-old male (G. 19, 187/218, HNJ016), was found with several healed antemortem and unhealed perimortem injuries, the latter on the cheek and neck must have been lethal. Artificial cranial deformation produced by two bandages can be seen on a male individual of G. 6 (76/86, HNJ005), part of pedigree 2 of European ancestry. This habit is rare in the Avar period, and was practiced only by some communities in the Transtisza region.

Archaeology

By Zs. Rácz

The graves formed an east-west oriented row, the burials were oriented north-south, and the deceased were laying in rectangular pits. A coffin or a wooden construction was observed in three graves, and in four cases there were benches along the long sides of the grave. No ceramic vessels have been found at all, but in 13 cases animal bones/food offerings have been placed next to the deceased. In the middle of the cemetery, there were two rich male graves with silver belts (G. 19 and 12), the founder of pedigree 1 buried with a horse and his son buried with horse harness and a Byzantine gold coin. The grave goods of the burials nearby included clothing accessories and jewels, like silver earrings and silver beads, and simple utensils (iron knives, iron belt buckles, spindle whorls). The Byzantine coin and the ^{14}C analyses allow the central male graves to be dated with a high probability to the 2nd half of the 7th century: 662–672 cal AD and 664–711 cal AD (68%)[30].

The north-south orientation and the appearance of the horse harness as status symbol in the graves are cultural elements that are also typical of the contemporaneous burials at Rákóczifalva. The latter has occurred sporadically in Eastern Europe since the Hun period and appears to have been revived in some TT communities in the 7th century.

Kunpeszér, Felsőpeszéri út (Bács-Kiskun county, Hungary)

During a road construction in 1893, a grave was found that contained a pair of pyramid-shaped gold earrings, toilet tools, and a pair of P-shaped sabretache suspension loops, which are known in the literature as the Peszér-Adács findings[31]. On the same site (47° 4'39.11"N; 19°14'33.45"E), between 1982 and 1984, a rescue excavation of approximately 15,000 m2 on a law sand hill was conducted by Elvira H. Tóth; in the process, a Sarmatian cemetery with a large number of graves and a Sarmatian settlement were discovered, and 32 graves from the Avar period were found next to them. After a short preliminary summary of the cemetery[32], the archaeological material was evaluated by Csilla Balogh[33], the anthropological analyses were carried out by Antónia Marcsik[34] (Extended Data Fig. 2). The 32 graves in the cemetery contained 33 individuals, from which 30 genetic samples were taken.

Anthropology

By A. Marcsik

The skeletal remains were categorized into an early and later burial group. Among the early graves 12 adults (7 males, 5 females) and 3 subadults were identified. The subadults were represented by young children of whom one was a neonate. The demographic distribution of the later burial group did not differ from the earlier with the 14 adults (8 males, 6 females) and 4 subadults. In the later burial group an adolescent individual was also identified among the subadults. Although the sex ratio appears to be balanced, there are relatively few subadults, and the small sample size prevents the drawing of any significant conclusions. The individuals buried at the site cannot be interpreted together, since the IBD analysis did not find a connection between the two burial groups. Only one traumatic alteration, a parry-fracture was observed on a 46-51-year-old female (G18, KUP007). The parry-fracture refers to a single fracture in the shaft of the ulna, usually caused by a forceful impact to the forearm as it is raised to defend the face.

Archaeology

By Cs. Balogh

32 graves were excavated and are divided in two groups: 15 burials (6 males, 6 females and 3 children) belong to the first group; they had NNW-SSE orientation and were laying remarkably far (20-40 meters) from each other. Five males were buried with gold- or silver-plated swords with P-shaped suspension loops and bows (Supplementary Fig. 7). The suspension loop of the sword from Grave 3 is the closest parallel to the one known from the high-ranking burial at Csepel. Their belts were decorated with mounts and strap-ends made of silver. The custom of placing an obol in the grave and the presence of goat/sheep bones served as food offerings were also observed in the cemetery. Jewelry made of precious metals (earrings with spherical pendants, pyramid-shaped earrings, etc.) and objects of everyday use (mirrors, pouches, capsules etc.) are characteristic for female burials. The first burial group of

Kunpeszér belongs to the so-called Kunbábony-Bócsa group (early Avar elite graves from the DTI). This small cemetery dated to the late 6th - first half of 7th century can be interpreted as a group of high-ranking families belonging to the military retinue of the Khagan.

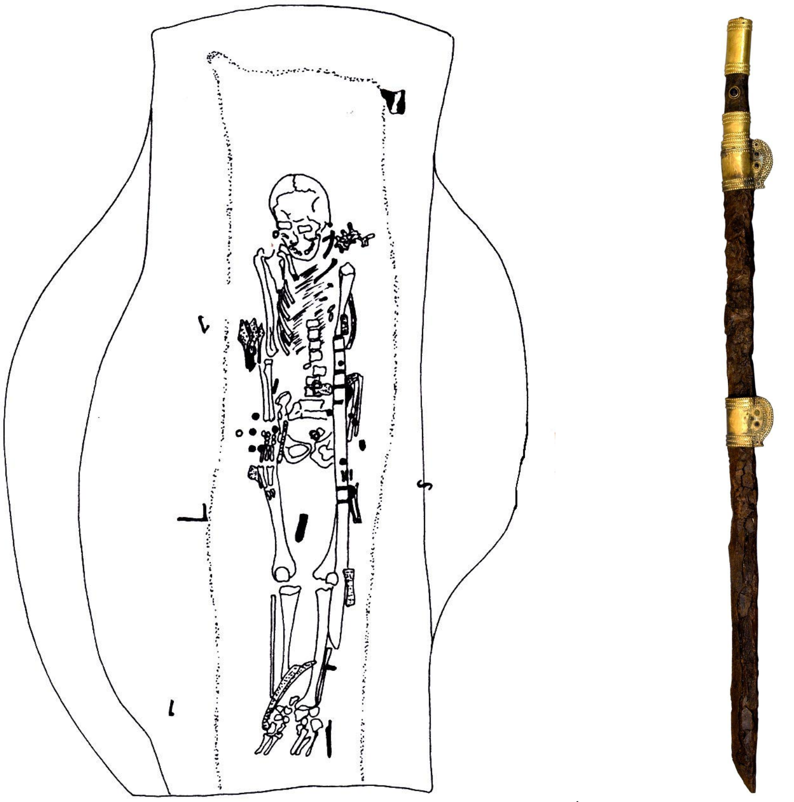

1 2

Supplementary Fig. 7 - 1: Kunpeszér, Grave 3; 2: Sword with sheet gold decoration and P-shaped suspension loops from the grave[105].

Results of a previous genetic study showed that among the elite individuals of the Danube-Tisza area, several samples belong to the same Y-haplotype within the N-Tat haplogroup, which can indicate paternal kinship between the individuals[35].

The second burial group contained 16 disturbed burials with individuals showing European morphological traits as well as mostly European ancestry (see following chapters). These poorly furnished (or even without any kind of grave goods) burials probably belonged to the lower social strata of the Avar society. This cemetery group is dated to the 8th century and shows no connection with the first group. The archaeological material is stored in the Katona József Museum, Kecskemét while the anthropological material is located in the Department of Anthropology at the University of Szeged.

Kunszállás, Fülöpjakab (Bács-Kiskun county, Hungary)

The cemetery of Kunszállás-Fülöpjakab (46°45'2.92"N; 19°44'16.68"E) was excavated by Elvira H. Tóth between 1967 and 1979. The archaeological material was evaluated by Gabriella M. Lezsák, and the skeletal remains by Balázs Gusztáv Mende [36,37] (Extended Data Fig. 1). The 61 graves in the cemetery contained 63 individuals, from which 61 genetic samples were taken.

Anthropology

By B. G. Mende

61% of the skeletons were well-preserved enough to conduct metric and macromorphological analyses on them. The demographic picture of the deceased shows a ratio of 25 subadults to 35 adults (41.7% to 58.3%). Subadult individuals especially concentrate in the last 2 generations of the pedigree. There are more women, especially among younger and middle adults (18–39 years) where they are heavily overrepresented (14 females compared to 4 males), which could be explained by the propagative age characteristics of women. Among individuals estimated to be over 40 years of age, the sex ratio is balanced. The height measurements are evenly distributed, the average height of both sexes falls in the upper range of the medium category. The character of the viscerocranium, and the mean values of the nasomalar and zygomaxillary angles show a pronounced straight facial profile in case of the Kunszállás-Fülöpjakab skulls. The morphological analysis of the teeth indicates the occurrence of shovel-shaped incisors.

Based on the results of the paleopathological analysis, the skeletal material does not show any special cases, with degenerative alterations of the vertebrae and arthrotic changes of the limb joints being the most commonly detected lesion types. The only exception is an adult male from Grave 9, whose left humerus and one of the ribs on the opposite side exhibited healed fractures[37].

Archaeology

By G. M. Lezsák, T. Vida

The site lies on a NW-SE oriented sand hill covering an area of 900 m2. The 61 graves with NW-SE orientation were organized into loose rows: 13 males, 22 females, 5 juveniles, 20 children were buried here. Loosely connected groups of graves can be observed. Coffin use was common, as indicated by the brownish-black imprint of the coffins and the coffin nails. In several cases the coffin was placed on a stand (?), the discolouration of the soil caused by it, forming round or rectangular spots in the four corners of the graves, can be observed in 22 cases. The depth of the graves ranged from 90 to 245 cm, but could not be associated with the social status of the buried.

Food offerings were observed in 41 graves (67%) and were present in all male (13), female (18) and child (11) graves (sheep, chicken, goose, pig, horse). Burial of a sheep rump, typical of nomadic peoples of the Eurasian steppe, was found in 18 graves (30%). Eggs were found in 5 graves. 17 graves (28%)

contained ceramic vessels for food or drink offerings, which were not found in the middle Avar (second half of the 7th c.) but in the late Avar part of the cemetery (8th c.).

Based on the grave finds it can be estimated that the site was used from the second half of the 7th until the end of the 8th century. Male burials with decorated belts are well represented in the cemetery. The chronological frame of the site is given based on the technical details, form and decoration (pressed, platedcast with griffin and tendril pattern) of the belt-fittings. The burials were also richly furnished with weapons: mainly bows, sabres, swords and knives came to light. The higher social status of certain individuals is represented by gold artifacts. The man from Grave 32 – the founder grave of the cemetery – was buried with gold earrings, golden hair clips, gold-foiled strap-ends with interlace pattern made of silver and a sword decorated with gold plates (second half of the 7th century). The burial with its prestigious grave goods could be rated to the single burials of the military elite from Kecskemét-Miklóstelep, Ballószög, and Szeged-Átokháza in the middle Avar period (the so-called Ozora-Tótipuszta group), at the same time its belt set also connects it to the earlier Kunbábony-Bócsa group. From Grave 52 a gold-foiled cast bronze belt set with griffin and floral ornament came to light. The inwrought bronze belt mounts with gold-foiling and palmette motif prove that the cemetery survived until the second half of the 8th century. Compared to the small number of the burials at the site, the percentage of belt sets pressed of gold sheets (Graves 24 and 32) and gold-foiled bronze belts (Graves 20, 52 and 59) are very high.
The cemetery could be interpreted as a burial site of members of the military retinue and their families in the close vicinity of the political center. Cemeteries in the region (Hetényegyháza, Tatárszentgyörgy, Kunadacs, Kunbaracs, Kecskemét-Ballószög, Szabadszállás-Batthyány utca, Gátér, Kiskőrös-Cebe, Kiskőrös-Vágóhíd, Tiszakécske-Óbög, and Homokmégy-halom, Solt-Szőlőhegy, Madaras-Téglavető, Szeged-Fehértó B) suggest that this area remained a seat of power until the 8th century.

Spatial distribution of pedigrees and unrelated individuals in the Rákóczifalva cemetery

A comparison of the cemetery map and the pedigrees reveals that the spatial distribution of burials strictly follows the biological relatedness described in the main text (Fig. 2). All major pedigrees and members of minor pedigrees are found in one cluster each (a few exceptions: some members buried in another cluster from pedigree 2, 4, 6). Regarding smaller lineage units, reproductive partners are often buried next to or close to each other. In 16 out of 34 cases they lied directly next to (ca. 0,5–2 m), 8x close to each other (ca. 3–10 m), 6x in larger distance but in the same cluster (ca. 11–25 m), and 4x far (e.g. opposite side of the grave group or in different groups, ca. 40–150 m) (Supplementary Fig. 8).

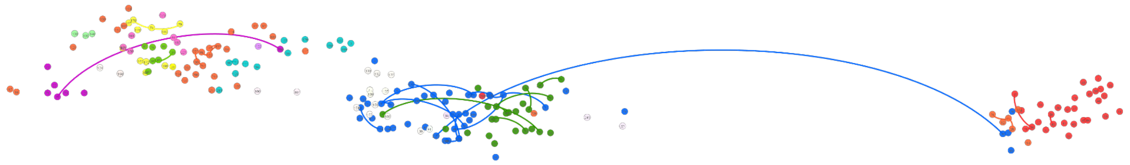

Supplementary Fig. 8 - Spatial distribution of reproductive partners in Rákóczifalva (Map: Levente Samu).

In case of more reproductive partners one is buried next to each other, the other(s) at a larger distance (e.g. RKF042/G. 61 male and RKF140/G. 223, RKF144/G. 229, RKF028/G. 44 females from pedigree 4). Subadult individuals are buried close to parents (Supplementary Fig. 9), although there are exceptions: some young individuals on the edges of the cemetery (RKF162/G. 4, RKF038/G. 55 from pedigree 4). But even adult male siblings are often close to each other, forming a cluster of close relatives (e.g. pedigree 15).

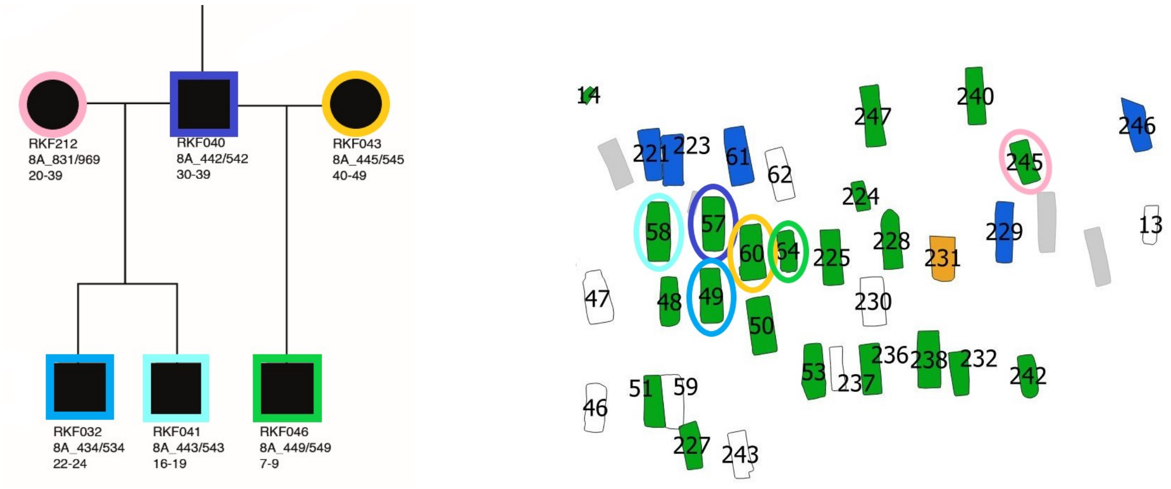

Supplementary Fig. 9 - Position of a small lineage unit (father, two mothers, and three sons) from the late Avar pedigree 5 in the middle part of the cemetery.

When both the parent/s and the descendants of a man (reproductive female partners and children) are present, there is a tendency that the "new family" is buried closer to him than the parents (e.g. RKF042/G. 61 – father – and RKF142/G. 226, RKF152/G. 233, RKF218/G. 5 – adult sons – from pedigree 4). Juvenile individuals, both males and females are buried next or close to the parents: females next to the mother (4x) if there is no mother, to the father (2x); males close to the father or the parents (5x), if there is no father, to the mother (1x). Young males between 18–22 can be buried next to other male relatives, cousins and uncles (e.g. RKF131/G. 197 from pedigree 2). For women, this age group

disappears completely from the lineages and is represented already by young reproductive female partners. In some cases adult children's (sons) graves were next to the father, father's wife, and half-brothers (RKF040/G. 57 in pedigree 5). Brothers and half brothers are often buried close but it occurs that one of them is in a larger distance next to other ('mightier') cousins and uncles (RKF131/G. 197 in pedigree 2). Small groupings according to sex and status are also observable: adult men (mainly those who have their own family) form a small cluster together with a group of exogamous reproductive female partners and non-related women (e.g. in pedigree 2, pedigree 4).
Although in cemeteries with a substantially smaller number of graves, the same pattern can be found in Hajdúnánás and Kunszállás (Extended Data Fig. 1, 5.).
In addition to those who are biologically related to each other, there are also unrelated individuals (individuals without evidence of close biological relationships or reproductive partnerships with others in the pedigree) in the entire area of the cemetery (Fig. 2). Their chronological position (if known) corresponds to the pedigree members to which they lie close. They can be buried 'solitary' in individual 'family clusters', or forming small groups with exogamous women, men without a 'detectable' partner, or even men where multi-partnership can be proven. This situation suggests that unrelated women can be linked to pedigrees and especially to male members of pedigrees. A good example is the case of pedigree 6, where 5 unrelated adult women lied near 6 adult men.

Pedigrees, related and unrelated individuals and status

The descent, gender, age, and birth order of the individuals can all closely correlate with their status. It can be evaluated by comparing archaeological features with the position in the line of descent. The founding role in the life of the communities may have been of particular importance. Founder males (or, in case of brothers, one of the males) were buried in several cases with valuable grave goods considered status symbols: in RK, horse harnesses and belt sets in the early, and belt sets in the middle/late Avar period. Pedigrees 2, 6, and 7 in the early and 3 and 4-5 in the middle/late Avar period are examples of this (Supplementary Fig. 10).
There are only one or two burials per pedigree in the early phase, primarily male siblings, which contain status indicators. The large middle/late Avar pedigrees are more complex. Particularly striking are the cases of the founders of pedigrees 3 and 4-5: one with a pair of siblings, the other with a set of siblings of two and six individuals (related to each other to the third degree), respectively, but only one individual from each was buried with a belt set (RKC033 in pedigree 3 and RKF042 in pedigree 4-5). In the later period, we find that the habit of burying a belt with the deceased is passed down from father to son. This is, however, only observed in one of the late pedigrees, and that is pedigree 4 in Rákóczifalva. Another example is the case of Hajdúnánás: in the middle of the grave row there are two male burials, a father (the founder, HNJ016, G. 19) and son (HNJ011, G. 12) surrounded by (biologically) closely related individuals. Both wore silver belts, the older was buried with a horse, the

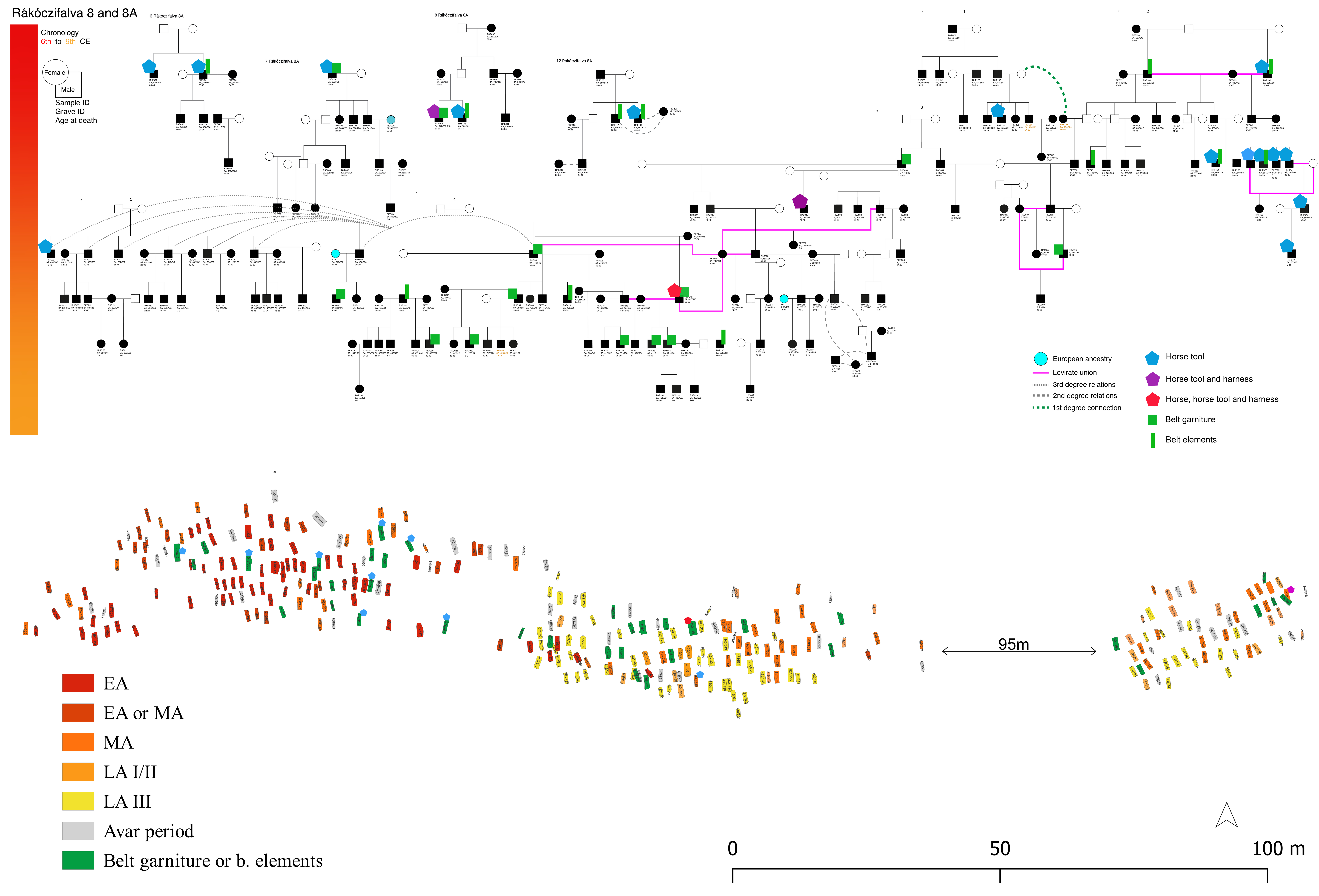
Rákóczifalva 8 and 8A
Chronology
6th to 9th CE
Female
Male
Sample ID
Grave ID
Age at death
6 Rákóczifalva 8A
7 Rákóczifalva 8A
8 Rákóczifalva 8A
12 Rákóczifalva 8A
European ancestry
Levirate union
3rd degree relations
2nd degree relations
1st degree connection
Horse tool
Horse tool and harness
Horse, horse tool and harness
Belt garniture
Belt elements
95m
EA
EA or MA
MA
LA I/II
LA III
Avar period
Belt garniture or b. elements
0
50
100 m

Supplementary Fig. 10 - Distribution of belt sets and horse tools in the pedigrees 6, 7, 8, 12 and 1 to 5 of Rákóczifalva. EA: early Avar period, MA: middle Avar period, LA: late Avar period.

younger with a gilded horse harness and a Byzantine gold solidus. Another son of the founder was interred with a simpler gold belt set, however, he died at a young age (HNJ014, G. 17). At the Kunszállás site, similar phenomena can be observed. The founder's grave contained the most valuable grave goods in the cemetery. Although his sons are likely not buried here, six of his male grandchildren are. Only two of them received belts, one of whom died at a young age. Children's rich equipment may represent a desired but unfulfilled status. These observations are potential evidence for heritable rank in the middle and late Avar periods.

Furthermore, within a pedigree, men with the most biological connections (intra- and intergenerational links, that is links to women who had reproduced with lineage men and to children) are founder men – in late adult/mature age – and their direct descendants. In the case of founder brothers, based on archaeological material and the extent of their families, one of them had a higher status (the above-mentioned RKC033 in pedigree 3, and RKF042 in pedigree 4 with 9 and 11 intra- and intergenerational links, respectively).

There might have been a difference in status not only at the individual level, but also between (sub)lineages. Judging from the archaeological material, the individual lineages/pedigrees are not of the same rank. Status objects and customs considered status indicators do not appear with the same frequency in the pedigrees. A good example is the case of contemporaneous pedigrees 4 and 5 related to each other–the founder brothers are third-degree relatives–, where No. 4 contains 12 belt sets or belt parts, and a burial with a whole horse and horse harness, while No. 5 only one grave – a child – with horse harness. This situation indicates the existence of collateral lineages of different ranks.

The striking differences between lineages/pedigrees and within pedigrees suggest the existence of primogeniture, a common phenomenon in steppe societies[38].

As described in the main text, 202 individuals (132 males, 70 females) had at least one close relative at the RK site and only 64 were unrelated. 36 of the 70 related females were exogamous reproductive partners. There is a strong sex bias among the 64 unrelated individuals as there are 51 females and only 13 males. The vast majority of these women are young adults, whereas males have a more balanced age distribution. Their position in the 'pedigree clusters', chronology, burial customs, and grave goods suggest that they are most likely exogamous partners of lineage men, who did not reproduce, or whose children left the site, therefore are not detected as biologically related but still part of the burial unit. The proportion of young adults is higher among unrelated females than among related females, but the ratio of older adults is the opposite between the two groups (Supplementary Fig. 11). These may have been exogamous partners who had died during pregnancy and childbirth.

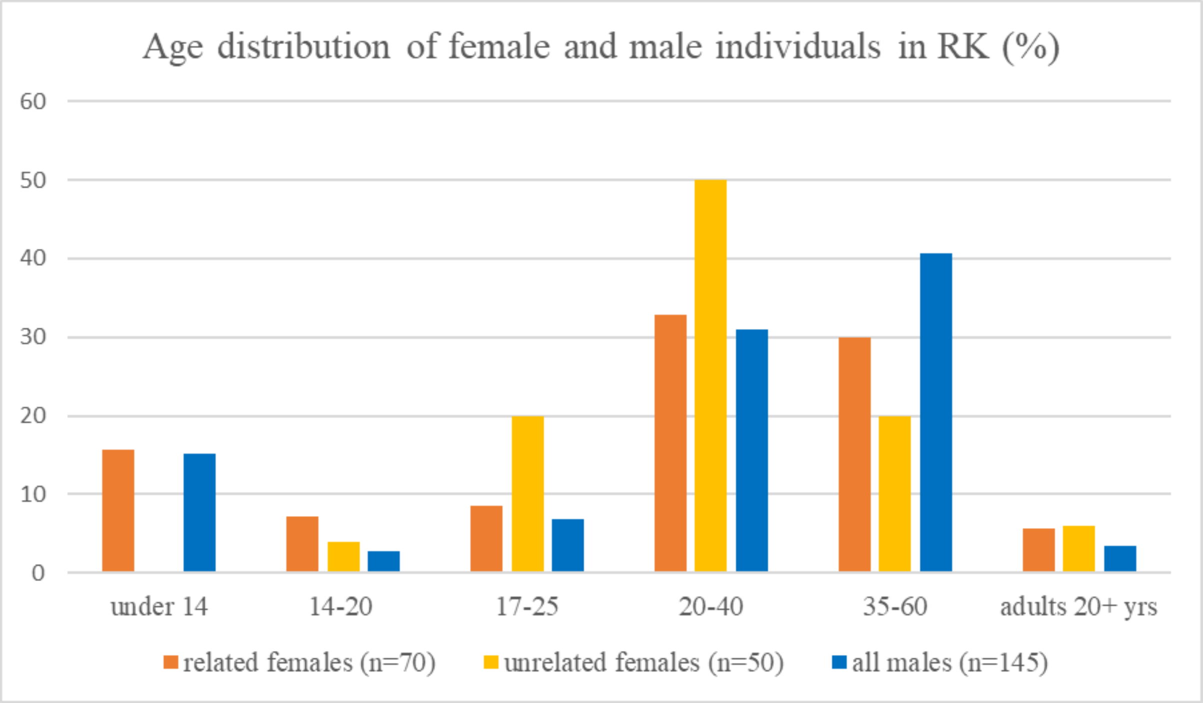

Supplementary Fig. 11 - Age distribution of female and male individuals in the RK cemetery.

When comparing grave goods, unrelated females have the same type of jewelry and utensils as related ones: earrings, beads, bracelets, belt buckles, spindle whorls, and iron knives are the typical finds in their graves. The average quantity of grave goods is also similar in the two groups. Some of the customs typical of the Avar cemeteries in the Transtisza region first appeared in the burials of women–both of exogamous reproductive partners and unrelated females–in Rákóczifalva, like niche-graves (RKF061/G. 88 and RKF132/G. 198), and animal skins/partial ruminants in the graves (RKF056/G. 84, RKF174/G. 76).

Unrelated males might have been of different status: there are two richer graves with belt sets, one from the earliest and one from the latest phase of the RK cemetery (RKF071/G. 102, RKC041/G. 54), but the majority of them are more modest, with no or simple grave goods. RKC041/G. 54 is particularly important because it demonstrates that a man outside the large middle/late Avar pedigree system (pedigree 2-3-4-5) in the latest period could have had a social status similar to that of lineage members. In some cases, a lower status may be associated with unrelated individuals: such is the case of RKC004/G. 8, a very shallow female grave with no grave good next to the burial of a woman belonging to pedigree 4. Children appear only exceptionally among unrelated individuals (RKF233/G. 212), implying that sending young individuals to other families/communities was not common in the Avar society segment we are studying.

Reproductive partners

In Rákóczifalva, a total of 80 cases of reproductive partnership were detected, 47 of which were part of multiple partnerships. We discovered 15 cases involving one male and 7 cases involving one female with multiple reproductive partners. Males have two partners in 10 cases, three partners in four, and four partners in one case (RKF042, 35-49 years old, founder of pedigree 4). The majority of these are older men (7 mature, 4 adult/mature, 2 adult, and 2 unknown individuals). An exception is RKF012, 25-29 years old, the son of RKF042. Female partners' young ages at death may indicate serial monogamy (RKC011: 40-55-year-old male and female partners RKC010: 20-24 years old, RKC013: 18-22 years old), but older females in multiple partnerships suggest polygyny (RKF042: 35-49-year-old male with four reproductive female partners, among them RKF028 and RKF140 are 40-49 years old. RKF180: 45-55-year-old male with two female partners, RKF061 40-59 and RKF185 35-50 years old). Multiple reproductive partners were also discovered in Hajdúnánás and Kunszállás (one and four cases, respectively). Regarding one female with multiple reproductive male partners, most of the cases (5) are levirate matches, that is male partners are biologically closely related. The custom was practiced from the early to the late Avar period. All the known females in such relationships are older individuals (RKF185: 35-50, RKF140: 40-49, RKC007: 50-59 years old), mostly connected to older males (e.g. the above-mentioned RKC007 with 45-55 and 55-59-year-old reproductive male partners). Due to the high age at death of the females, serial partnership is a more plausible explanation for these unions. A unique case is RKF140 (40-49 years old) with four reproductive male partners (two different levirate unions, three of the four males with late adult or mature age at death), where concurrent partners are conceivable.

Only 5 cases of biological relationships between exogamous or non-lineage females were found (siblings: RKC007-RKC017, HNJ010-HNJ011, 1st degree: RKF128-unknown, 2nd degree: RKC044-RKF150, RKC019-RKC015), implying that female partners came from diverse communities.

Based on the pedigrees, we can also conclude the possible beginning of the reproductive age (though this must be supported later by anthropological examinations on childbirth): The youngest mothers were 18-20 (1x) and 18-22 (2x) years old, while the youngest fathers were 24-29 years old (2x) at death. This is consistent with observations that juvenile individuals are buried next to their parents (females to 16-19, males to 18-22), and lineage females disappear from the pedigrees in late juvenile / early adult age (18-20).

Social organization in the Avar Khaganate and its parallels in historical steppe societies

By Zs. Rácz

Recent genetic research suggests that the eastern peoples who founded the Avar khaganate did not originate from the same region, as confirmed by archaeological finds and historical sources: diverse populations from the Eastern as well as the Western Eurasian Steppe coexisted. The Carpathian Basin's central region saw a concentration of Eastern Central Asian immigrants, and archaeological evidence suggests that this region served as the primary settlement area and power center for the Khaganate's ruling clan. The genetic and archaeological picture is more mixed in the Transtisza region, where certain population groups probably came from the Pontic-Caspian steppe. However, based on the four cemeteries we examined, the main characteristics of the social organization were the same in communities with different eastern ancestries and reflected the historical steppe peoples' social structure. These shared characteristics are: patrilineal lineages, patrilocal residence, female exogamy, the occurrence of polygyny and levirate unions, large descent groups and the avoidance of marriage between biological relatives. This system persisted throughout two and a half centuries in the Avar period, and groups with similar social practices seem to have intermarried preferentially. It should be noted that this system differs from that described in previous articles from the Carpathian Basin's pre-Avar era, between the fifth and sixth centuries[39].

It is assumed that a change of lifestyle took place in the Carpathian Basin during the 7th century, when originally nomadic or semi-nomadic communities from the East settled down. This process is perceived through the growing number and spread of settlements that have been used for a long time, as well as through the changes in diet of the Rákóczifalva population (see main text and Supplementary Information chapter below). We can expect the social structure to change in parallel with these transformations, but there is a surprising constancy, at least as seen through the genealogical aspect. The size of the lineage groups buried together, however, has changed: smaller pedigrees can be reconstructed in the early Avar period, and larger ones in the middle and late Avar periods. This shift is most likely to be linked to the emergence of larger cohabiting communities.

Regarding *kinship terminology*, we have no written records for Avar kinship terms, but patrilineal organization and exogamous patrilineal clans support the idea that a type of Omaha system, which was prevalent in Central Asian steppe societies, may have existed. Here, the social pressure derives from distinguishing who belongs in one's patrilineage and who does not. This indicates that kin are divided into groups based on their descent: those who are patrilineal are referred to by one set of terms, while those who are connected through women (by marriage) are referred to by another set of terms[40]. The kinship terminology reflects social practices, most importantly, who may be available for marriage. However, it is also important to point out that cross-cousin marriage, a common practice in unilineal

descent systems[41], was not at all characteristic of Avar society, as revealed by genetic data. Another important factor in Omaha terminology is distinguishing age, gender, and generation on the paternal side, as this system gives authority to the oldest male member in the patrilineage.
A combined analysis of archaeological and genetic data suggests that birth order may have been important in the Avar kinship system too. This is indicated by the different equipment of graves between brothers, half-brothers and paternal cousins, and even the emergence of collateral lineages of different rank can be detected (pedigree 4 and 5 in Rákóczifalva, Supplementary Fig. 10).

The elementary unit of *social organization* in steppe societies is the patrilineally organized family. In tribal genealogical kin type, "a family may grow into a lineage group and the latter into a tribal subsection composed of several lineages, without any radical change in the nature of functions of the group"[42]. Patrilineal genealogies are the constitutive elements of the society, and within them, descent lines are traced and ranked according to birth order of the male founders. The knowledge of genealogies are preserved to modern days, and children as young as 10 or 12 can trace their ancestors back many generations, including those of closely related collateral lines[38]. This concept results in a strictly hierarchical structure in the smaller as well as the larger units of society. Thus, the hierarchical system can also be observed at the clan level (conical clan system), with the ruling clan at the top. Clans wish to move higher up the hierarchy by marrying members of higher ranking clans[43]. We can speculate that this phenomenon is manifested in our observation that the burial of Kunbábony, which is a rich solitary burial interpreted as a possible khagan grave based on status symbols found in the grave, had the highest number of between-sites IBD connections among all of the new and published individuals analyzed (Extended Data Fig. 6b).
The system's ability to wax and wane, fusion and split, has obvious advantages, particularly when a large horde must be brought together militarily quickly. Large organizations, on the other hand, cannot survive for long because they easily fragment.

The smallest level of society–family and family related groups–was based on genealogical relatedness and economic relationships, as described by N. Kradin in the case of the Rouran khaganate. The higher level–clan grouping–was probably based on more distant, partly fictitious, genealogical relationships, and more importantly reciprocal and other communal relations. The highest level of links between tribes might have represented political, cultural, ideological, and economical relations, and fictitious genealogical relationships[44].

As antecedents and parallels to Avar society, the kinship system of the East-Central Asian steppe populations of the period can be considered. Proto-Turkic kinship system is described as a 'tribal genealogical' kin type[42]. Old Turkic kinship system can be reconstructed on the basis of the Orkhon

inscriptions from the 8th century, i.e. it belongs to the Omaha system, showing a patriarchal character[42]. In the Seljuk era (11th-12th c. AD), the kinship terms of several Turkic tribes in Central Asia were changed during the sedentarization process[38,42]. Due to a lack of written records, such changes in the Avar Khaganate cannot be investigated.

Inheritance of authority is recorded among the elite stratum of society: e.g.Chinese sources mention 16 throne successors in the Rouran khaganate (5th century - first half of 6th century AD); half of them were direct successors, and regarding the other half, the title passed on to the collateral line to brothers or from uncles to nephews[44]. Only for the ruling class did genealogy survive, with Chingis Khan's 24 generations of ancestors recounted in History of the Mongols in the 13th century[45].

The social structure, the steppe power's pyramid can be reconstructed based on Chinese sources. For example, the hierarchical levels of the Rouran society comprised the head of the society (the *khagan* and his wife, the *khagatun*), the ruling elite, and the common people. The highest posts and the throne were inherited within the khagan's clan. The dignitaries were classified as high and low ranking, and sources also mention other terms such as military commanders, chiefs, and elders. "In all probability, these terms refer to a wide circle of persons, which comprised the nomadic aristocracy of the clan chieftains, the tribal chiefs and elders, the representatives of servitors who fled from China and other nomads who had served the Rouran for long." It is possible that posts were inherited within one related kin group (clan, lineage), and the khagan could also grant a specific post and title for particular services[44].

Moving to the question of the smallest *cohabiting communities*, to the exact type of families, their form of coexistence is uncertain. In anthropological literature, the nuclear family, the joint family and the extended family have been mentioned as basic units of nomadic society[38]. According to A. Khazanov, in the Eurasian steppes the nuclear family and a variant of it, patrilocal stem-family, were predominant. Additionally, he introduced the notion of 'primary kin groups', which represents a less stable group than an individual family: "a number of separate and independent families which are very closely connected with one another through ties of kinship, reciprocal relations, common residence, etc., the core of which is made up of very close consanguines (microlineage), who are descended from one close ancestor and who in the past have frequently made up one family – brothers, cousins, uncles, nephews, etc."(Khazanov 1994, 128-129)[46]. At the same time he supposed that when nomads became more and more sedentary, the size of their families and the number of generations in the families might have increased[46].

The genetic study of entire Avar cemeteries has revealed that, in accordance with the above, biological relationships were fundamental to the life of households as well as the larger communities/settlements that they formed. In the future, the size of the cohabiting family/household can be estimated using a combination of genetic data from cemeteries and settlement data (like size and number of buildings and household units).

Marriage was regulated by the order of descent and based on lineage exogamy. Intermarriage within the paternal line was only permitted after a certain number of generations, which could range between five and nine[42,47]. A favorable marriage created an opportunity to raise the status of the lineage. According to our evidence, among Avars, remarriage, polygyny, and a specific type of remarriage, levirate unions may have been practiced on a regular basis throughout the period. Levirate practice might be linked to individual status because it is almost exclusively found in connection with men whose burials include status objects.

Population shifts and the middle Avar-period transformation

By L. Samu

From the second half of the 7th century (middle Avar period) there were many different changes in the archaeological record in the Carpathian Basin, what we call the "middle Avar-period transformation"[48]. The influx of Byzantine coins was sinking, new types of belt garnitures, weapons, jewelry and horse harness appeared, the long-distance connections were also changing, and there were regional shifts in the spatial distribution of the settlements too[22,49]. The observed phenomena were explained and interpreted in many different ways[48,50–52].

The archaeogenetic analysis of the Rákóczifalva (RK), Kunszállás (KFJ), Kunpeszér (KPF) and Hajdúnánás (HNJ) sites has a great importance for this long-standing question and fundamentally changes the debate around this topic. In the case of Kunpeszér, after the first small early Avar-period group, a completely new and also genetically different community started to bury their ancestors from the Middle Avar period onwards (see the Kunpeszér chapter). In the case of the Rákóczifalva site we also observe a clear population shift: new individuals, although with the same genetic ancestry as the first ones (except for the Y-haplogroup shift from J1a to J2b), start a new life. Even though a small descent line of the original population also survived on the site (see chapter Rákóczifalva-Bagi-földek). The Kunszállás and Hajdúnánás sites don't have an Early Avar phase, which in itself proves the founding of new communities in these places.

The analysis of the strontium isotope data of three of the communities proved that the founding of these sites is rather the result of an intraregional mobility. Taking together the genetic, isotope and archaeological data we can assume that from the second half of the 7th century individuals and their reproductive partners left their original communities and in new places established new settlements. In some cases this new-founding was prosperous (like RK or KFJ) – the population increased to a large extent, in other cases (HNJ) the community remained small. The phenomena – called middle Avar-period transformation – is the footprint of these new communities, their living strategies and subsistence.

Migration history and admixture processes of the DTI and TT the Avar period population: evidence from population genomics, archaeology and history

Population genomic analyses performed on the newly sequence ancient genomic data show that 88% (Supplementary Table 1) of the Avar period individuals have detectable amounts of a Northeast Asian ancestry profile ultimately traceable to the eastern Eurasian Steppe with varying degrees of admixture with western Eurasian sources (with Northeast Asian ancestry ranging from 100% to 32%, highest and lowest per site median; Fig. 3). With such a dense sampling we are able to explore in more detail the process of these migrations and the genomic composition of the populations involved. We found key regional differences between the DTI (KUP and KFJ) and TT (mostly in RK) sites as well as temporal differences between the early and middle-late Avar period. Proceeding temporally, we confirm previous findings[5] that individuals with the highest and virtually un-admixed eastern Eurasian Steppe ancestry are found almost exclusively in the Early Avar period elite sites of the DTI (Fig. 3). In fact, all the early Avar period KUP individuals carry this genetic profile, except one 1st generation admixed infant previously published[5] and now part of pedigree 1 (A1817; Extended Data Fig. 2) and one new unrelated individual (Fig. 3).

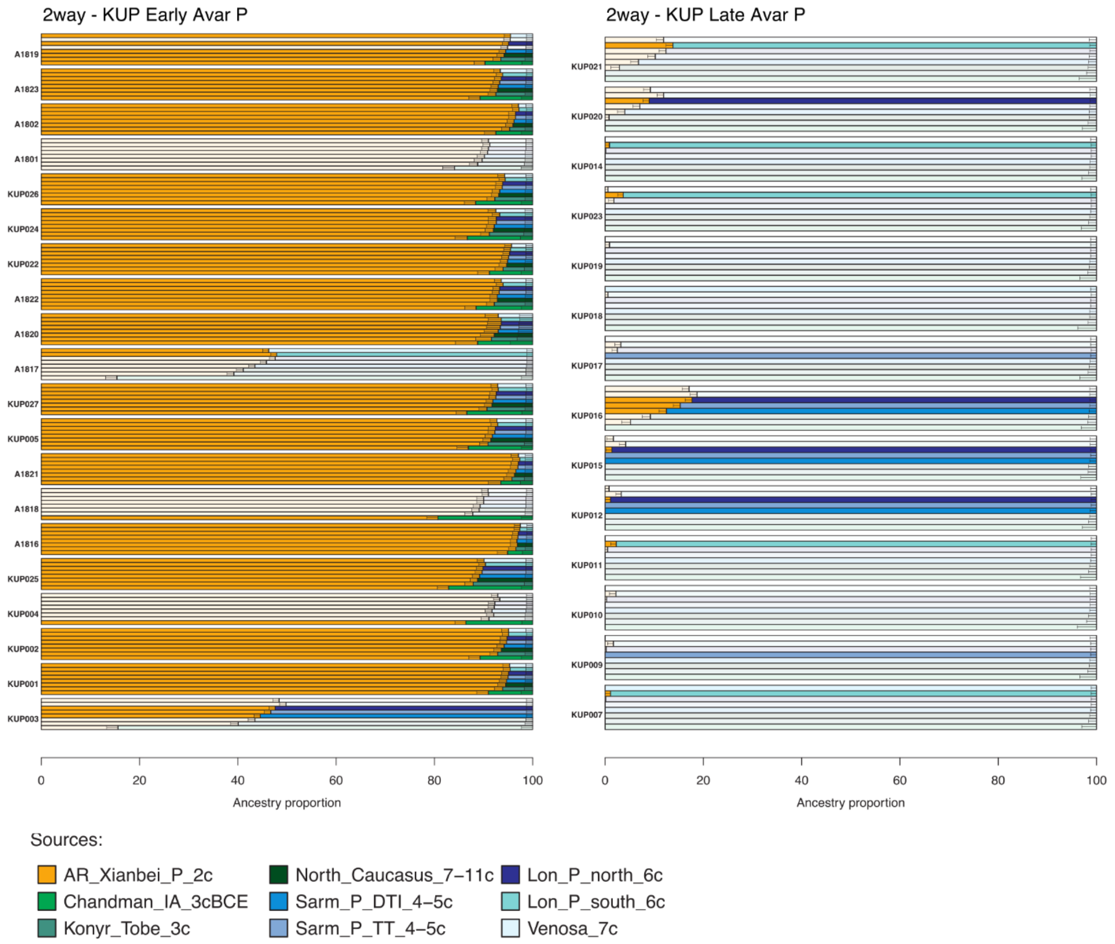

Supplementary Fig. 12 - qpAdm models of the individuals from Kunpeszér (KUP) site. A transparency factor is added to the models with poor fits (p-value < 0.05). For each model plotted (each single histogram bar), the *p*-values are calculated using a likelihood ratio test in which the constrained model (i.e. the qpAdm admixture model tested) is the null hypothesis and the non-constrained (testing all combination of "simpler" models by removing one source at the time) is the alternative hypothesis. No adjustment for multiple testing was not done as not applicable. These models are derived from the (n-1) x (m-1) *f4*-matrix with n the number of the target (1) and admixing sources (2-way = 2) and m are the 12 outgroup populations (Methods). Ancestry proportions are shown with one standard error, obtained using a standard jackknife approach of 23 independent runs, dropping a chromosome at the time (Methods).

We used qpWave/qpAdm following the framework reported in the Method section, as in[5] partly based on the evaluations from recent articles that tested performance of this approach under many different scenarios and parameters[53,54], to test which among contemporaneous or preceding populations available among published ancient genomic data would best describe this East to West Eurasian admixture (Methods; Extended Data Fig. 8a & b). With this modeling approach we do not intend to find the best-fitting models but rather aim to have enough resolution to be able to test models that we can reject compared to others. The method still produces many alternative fitting models (Supplementary Table 5) so we formulate our demographic interpretations based on the models we have the resolution to reject given the available data rather than on all the possible alternative fitting models.

In agreement with a previous study[5] we found that the source of East Asian component was highly homogeneous across all admixed individuals (in KUP and all the four sites) and matches the main eastern genepool found in the Eastern Eurasian Steppe from the Xiongnu (1st c. BCE/CE) to Xianbei (2nd c. CE) the Rouran period (6th c. CE) (Extended Data Fig. 8; Supplementary Fig. 11). Except for the 2 admixed individuals mentioned before, all the early KUP individuals share the same virtually un-admixed profile as the other early Avar period DTI individuals (DTI_EA_east) published in[5] (Extended Data Fig. 8). Virtually un-admixed because they do have small proportions (~5-10%) of a not resolved Western source (Supplementary Fig. 11) but this is shared also with the Rouran period individual and most of the easternmost Xiongnu period individuals[5] and dates back to the middle of the first millennium BCE (Extended Data Fig. 9[5]), reflecting demographic events occuring in the Eastern Eurasian Steppe in the ancestors of all these groups. For this reason we use the DTI_EA_east group as our main proxy for this Northeastern Eurasian component in the models of the individuals from the other sites (Extended Data Fig. 8; Supplementary Table 5; Supplementary Fig. 13 and 14).

We also found that 8 out of 15 EA KUP individuals are genetically related and formed two pedigrees (Extended Data Fig. 2). Instead, the LA KUP individuals, in agreement with their different archaeological contexts, carry almost completely un-admixed European ancestries with few exceptions (Supplementary Fig. 11). For example, KUP016 female with a 15-20% eastern admixture also shares a

20cM IBD connection with the MA individual RKF163 member of the main RK pedigrees (Fig. 3; Supplementary Table 4). Among the LA KUP individuals, we reconstructed three small pedigrees, unrelated to the ones in the EA period, further confirmed by the absence of even more distant IBD connections between the EA and LA individuals at KUP (Fig. 3).

In the TT region instead, already in the earliest stages of the early Avar period the RK individuals have a lower proportion of East Asian ancestry (median of 42% respect to 100% in KUP; Fig. 3) and are more heterogeneously mixed with West and Central Eurasian components (standard deviation of 17% respect to ~0% in KUP excluding the two first generation admixed individuals). In RK the Eastern Eurasian source is again very homogeneously matching DTI_EA_east, only 10% of the RK individuals require slightly different Eastern sources (Supplementary Table 5). The composition of their Western Eurasian ancestry components are instead more heterogeneous. Among the sources tested 15% of the individuals can only be modeled with a proxy of ancient Early Medieval populations from the northern Caucasus regions (North_Caucasus_7-11c) and 22% require a mixture of North_Caucasus_7-11c and unresolved Carpathian Basin proxies describe below (Supplementary Table 7; Supplementary Table 5; Extended Data Fig. 8c). Given the limitations in reference data available from across the Eastern European, Pontic and Kazakh Steppe during the 1st millennium CE, we caution in attributing too specific geographic signal to this ancestry and we refer to this ancestry proxy as a "North of the Caucasus Steppe" source. 33% of the individuals can only be modeled with an ancestry profile that is the most represented in the Carpathian Basin Sarmatian period (4nd to 5th centuries) (Sarm_P_DTI_4_5c and Sarm_P_TT_4_5c or Sarm_P_DTI_TT_4_5c when considered together; Supplementary Table 1; Extended Data Fig. 8c; Supplementary Table 5), that itself could be modeled with ~10% genetic contributions from earlier Iron Age Steppe sources[5]. Finally, 21% percent of the models work with an ancestry profile matching only he later 6th century individuals from the Longobard period site of Szólád in western Hungary (Lon_P_north_6c and Lon_P_south_6c or Lon_P_6c when considered together)[39]. These groups lack this steppe influx but are instead more heterogeneously spread along a European north to south axis of variation[39] (Extended Data Fig. 8b), which is the reason for keeping the more southern and northern group distinct in qpAdm, as in[5]. This same distinction is also found in our new individuals from RK Sarmatian period individuals that can be modeled as ~100% Sarm_P_DTI_TT_4_5c and the RK Gepid period individuals that can be modeled as 100% Lon_P_6c (Extended Data Fig. 8c). This 6th century profile seems to extend also in the Avar period, among the individuals that don't carry East Asian ancestry (Extended Data Fig. 8c) and some of them can be modeled only with Lon_P_north_6c or Lon_P_south_6c. Although some can still be modeled with Sarm_P_DTI_TT_4_5c. In fact, the two distributions of the Sarmatian period and 6th century Longobard/Gepid period individuals are in part overlapping, suggesting partial continuity between the time periods. We believe that for this reason it was not always possible to distinguish between the different sources and 64 models remain unresolved (Supplementary Table 7. Overall the un-admixed European individuals in the Avar period represents a minority among the new data (12% of overall

individuals across the 4 sites) and it includes either unrelated individuals or smaller pedigrees not connected to the ones composed of individuals with admixed ancestry. The only exception are 3 exogamous mothers in RK (RKF078 from pedigree 7, RKC013 from pedigree 3, RKF211 from pedigree 4) that carry 100% European ancestry and that had offsprings with admixed ancestry lineage males (Supplementary Fig. 10). The per-individual admixture dates, including only the individuals we can classify in a chronological phase, suggest that most of this admixture process occurred roughly a century before the beginning of the Avar period (median 490 ± 73 CE) and only 20% of the dates post-date 568 CE, which is the year of arrival of the Avars in the Carpathian Basin[1] (Extended Data Fig. 9). This percentage corresponds to the proportion of individuals we can confidently model with the Lon_P_6c component which reassures us on the fact that even if we did not have the resolution to resolve all the qpAdm models we are capturing a meaningful signal which is that a minor (although still around 20%) fraction of the admixture process that produced the ancestry profile of these communities occurred after they settled in the Carpathian Basin. It is in fact possible to observe a slight decrease of East Asian ancestry in the two sites, RK and KFJ, that span larger time periods.

Instead, the 33% of qpAdm models working with Sarm_P_DTI_TT_4_5c and the 22% that require also a 3-way mixture involving also the of North_Caucasus_7-11c component could suggest that between the Carpathian Basin and the Pontic-Steppe / North Caucasus region there was a gradient of ancestry between these two genomic profiles. With an increased affinity to Europe ancestry on the west and increased affinity to Caucasus ancestry towards east. Genetically, this should be formally tested producing ancient genomes from this time period across this region. Archaeologically this hypothesis would have some ground as Sarmatian cultures were in fact spread across this wider area, therefore assuming a pattern of genetic continuity with isolation-by-distance would be logical. Cultural connections, in addition to genomic ancestry, can support the link between the Carpathian Basin and the Eurasian steppes: it is traceable in certain burial practices, status symbols and prestige objects of the elites, elements related to fighting techniques, as well as in simple utensils[55,56]. At the same time, there are differences between the individual areas where the population of eastern origin settled, particularly during the Early Avar period. These primarily concern some aspects of burial customs, but there are also regional differences in material culture. The RK burial site fits well into the cultural context of the TT's central and southern regions in the late 6th and 7th centuries[57,58] (Supplementary Fig. 3). The graves are oriented N-S, a new influence from the East, as opposed to the W-E orientation that was common in Central Europe starting in the 4th-5th century. Animal skins (partial animals) were put in several graves (pedigree 2, 8, 11, and unrelated individuals), and hand-shaped vessels were placed next to the head of the deceased in 16 cases (pedigree 1, 2, 7, 8, 11, 20, and unrelated individuals). Although rare, complex grave forms such as end-niche graves can be found (pedigree 2, unrelated individuals). Overall, these phenomena are strikingly similar to nomadic burials of the 6th-7th centuries in eastern Europe, known as the Sivashovka (Ukr. Syvashivka) horizon, from the Pontic steppe, the northern Caucasus foothills, and the lower and middle Don-Volga rivers[19] (Supplementary Fig. 2). Based on this

context, archaeological research suggests that part of the Avar-period TT population originated in the Eastern European (also called Pontic) Steppe. In contrast, some cultural features of the elite of the DTI area, which includes the early graves of the KUP site, can be traced back to the Eastern Eurasian Steppe (for example, the crescent-shaped gold pectoral, a status symbol from Kunbábony or the ring-pommel swords covered in gold/silver sheets from several sites)[55,56,59]. These burials are associated with the leaders of the early Avar Khaganate and the Khagan's military retinue of East Eurasian origin. This archaeological association finds a striking parallel with the IBD network results as the individuals in KUP and KFJ are highly interconnected by many shared IBDs (Extended Data Fig. 6; Supplementary Table 4). In summary, our new evidences answer an open question regarding the composition of incoming eastern migrants[1,5]: together with a more homogeneous and un-admixed eastern Eurasian Steppe ancestry elite population found in the core DTI region of the Avar empire, more genetically mixed and heterogeneous communities settled in the neighboring TT region in the Carpathian Basin in the early period.

**Models**	**Number of individuals tested**	**Percentage (%) over total resolved**	**Percentage (%) over total tested**
2-way - **Sarm_P_DTI_TT_4_5c**	60	31.9148936	23.809524
2-way - **Lon_P_6c**	38	20.212766	15.079365
2-way - **North_Caucasus_7-11c**	28	14.893617	11.111111
3-way admixed - North_Caucasus_7-11c + Lon_P_6c **or** Sarm_P_DTI_TT_4_5c	42	22.3404255	16.666667
Other eastern source	20	10.6382979	7.936508
Total resolved	188		74.603175
unresolved after 3-way or failed and no alternative model found	64		25.396825
Total tested	252		

Supplementary Table 7 - Summary of best qpAdm models for Avar period RK individuals using the modelling rationale detailed in Methods. The first source is always DTI_EA_east, except for the 10% of models working with another eastern source (individual models are in Supplementary Table 5).

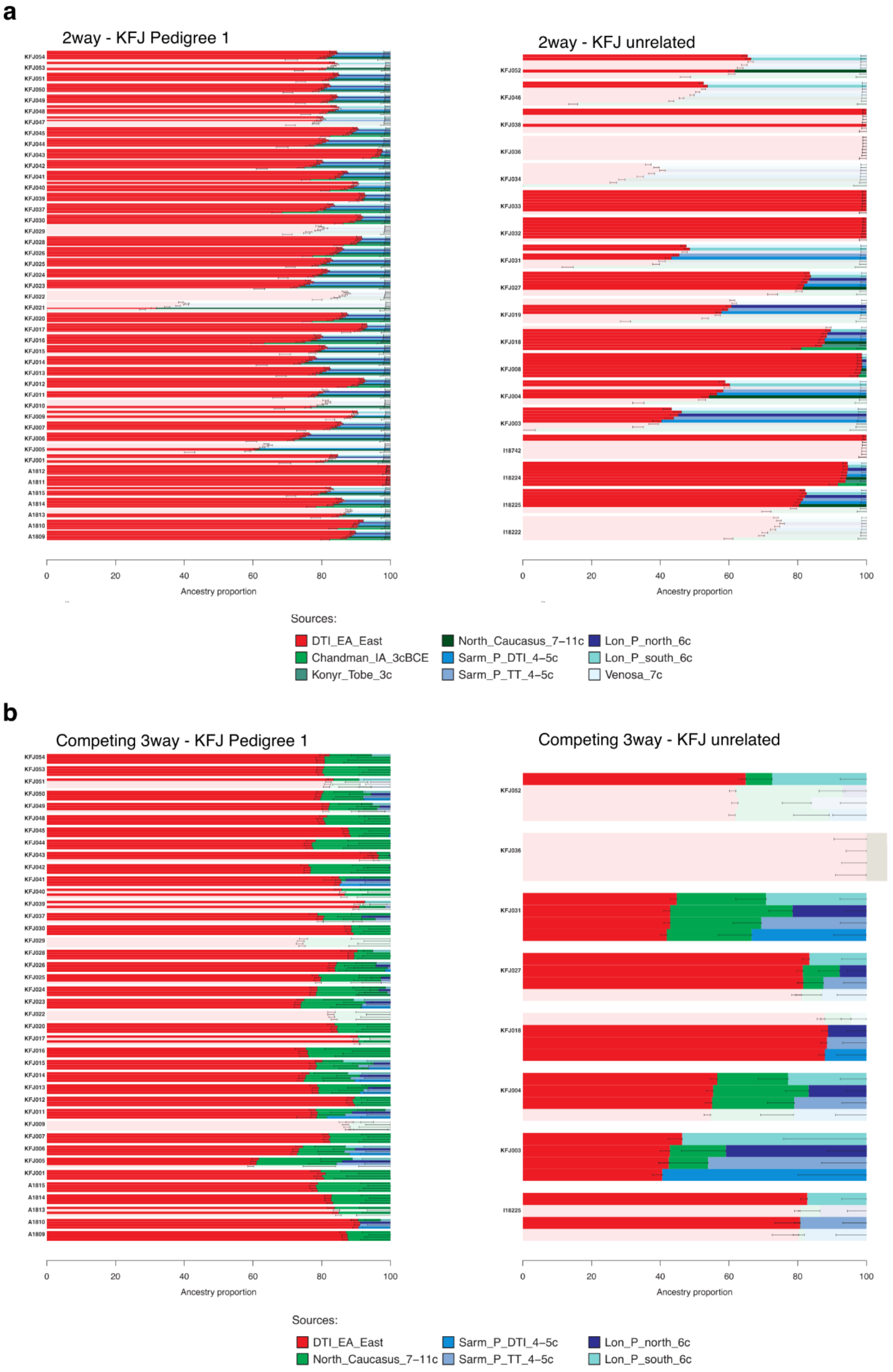

Supplementary Fig. 13 - qpAdm models of the individuals from Kunszállás (KFJ) site. A transparency factor is added to the models with poor fits (p-value < 0.05). **a)** 2-way and **b)** 3way models as described in the Method section. For each model plotted (each single histogram bar), the *p*-values are calculated using a likelihood ratio test in which the constrained model (i.e. the qpAdm admixture model tested) is

the null hypothesis and the non-constrained (testing all combination of "simpler" models by removing one source at the time) is the alternative hypothesis. No adjustment for multiple testing was not done as not applicable. These models are derived from the (*n*-1) x (*m*-1) *f4*-matrix with *n* the number of the target (1) and admixing sources (2-way = 2) and *m* are the 12 outgroup populations (Methods). Ancestry proportions are shown with one standard error, obtained using a standard jackknife approach of 23 independent runs, dropping a chromosome at the time (Methods).

Moving to the middle & late Avar period, we observe that the individuals from the site of KFJ in the DTI region preserve high eastern ancestry ranging from ~70% to ~80%, higher compared to what is observed in the early period in the TT, despite their later chronology (see first chapter of Supplementary Information). Of these, 45 out of 58 individuals are found to be genetically related forming a single extended multi-generation pedigree (Extended Data Fig. 1). Most of the individuals in this pedigree can be modeled with a ~20% to ~30% admixture with a component best matching the North_Caucasus_7-11c resulting after the competing 3-way model strategy described in the Methods and the admixture proportions remain highly homogenous despite the 6 generation extension of the pedigree (Fig. 3; Supplementary Fig. 13). We observe only one individual with significantly higher North of the Caucasus ancestry (>45%), a lineage son (KFJ021) whose mother was not found at the site. Interestingly, all the individuals dated to the MA transition period in KFJ, including the two mother-son pair who were founders of the pedigree (A1811 and A1812), carry the same un-admixed East Eurasian Steppe ancestry found in KUP and in the other EA elite sites as they can be modeled as 100% DTI_EA_East (Supplementary Fig. 13). On the contrary, the unrelated individuals have either an Eastern Steppe un-admixed ancestry or have higher admixture that, contrarily to pedigree 1, is mostly matching European sources and minorly the North of the Caucasus source (Supplementary Fig. 13).

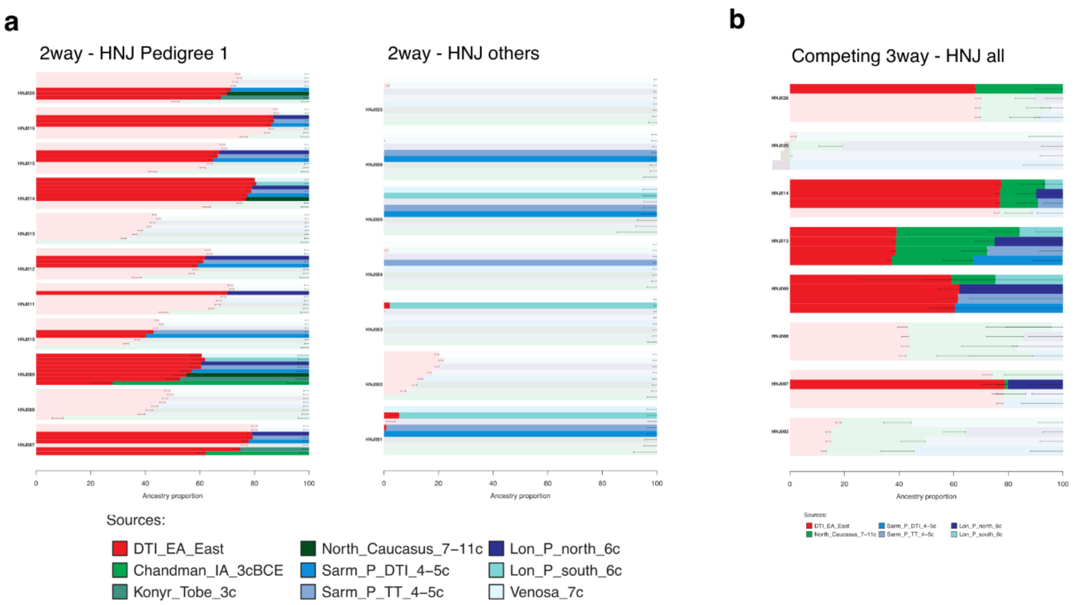

Supplementary Fig. 14 - qpAdm models of the individuals from Hajdúnánás (HNJ) site. a transparency factor is added to the models with poor fits (p-value < 0.05). **a)** 2-way and **b)** 3way models as described in the Method section. For each model plotted (each single histogram bar), the *p*-values are calculated using a likelihood ratio test in which the constrained model (i.e. the qpAdm admixture model tested) is the null hypothesis and the non-constrained (testing all combination of "simpler" models by removing one source at the time) is the alternative hypothesis. No adjustment for multiple testing was not done as not applicable. These models are derived from the (n-1) x (m-1) *f4*-matrix with n the number of the target (1) and admixing sources (2-way = 2) and m are the 12 outgroup populations (Methods). Ancestry proportions are shown with one standard error, obtained using a standard jackknife approach of 23 independent runs, dropping a chromosome at the time (Methods).

In the TT region in the middle Avar period site of HNJ, there is a clear separation between 11 individuals with high Eastern Eurasian ancestry (87%-40%), of which all except one are related in forming a single 3 generations pedigree (pedigree 1; Extended Data Fig. 3; Supplementary Fig. 14). The remaining 7 individuals instead carry European ancestries (Supplementary Fig. 14). Four of them are related, forming pedigree 2 (Extended Data Fig. 3). These two groups of individuals do not share any IBD connections between them (Fig. 3).

In the middle and late Avar period, the RK population admix further decreasing the East Asian ancestry proportion (median 42% in EA to 30% in MA and LA) becoming also more homogeneous around this average (standard deviation from 17% in EA, 15% in the MA to 11% in LA) (Supplementary Table 5). This fits well with the archaeological evidence, as the middle and late Avar periods in the Great Plain are characterized by a more homogeneous culture. This is evidenced by the related grave goods of the RK and KFJ sites (women's jewelry, men's belt sets) and burial customs common throughout the Carpathian Basin (graves with post holes/grave construction).

Possible historical contexts for pre-Avar-period admixture

According to the historical sources, the admixture between Eastern Asian and Pontic/Caucasian steppe ancestry which we estimated (Extended Data Fig. 9) to have happened well before the arrival of the Avars in the Carpathian Basin can mainly have taken place in three scenarios:

- First, it can be a consequence of the arrival of the Huns north of the Black Sea in c. 375, where they subdued Alans, Gothic groups and other previous populations of the region, and closely interacted with them. When the bulk of the Huns moved into the Carpathian Basin in the early 5th century, some Hunnic groups, for instance the Akatzirs, remained north of the Black Sea, and are attested there well into the sixth century. After Attila's death (453), Hunnic groups led by some of his sons returned to the Pontic steppe, where their traces faded before the end of the 5th century.
- Second, admixture may also have happened in the Carpathian Basin. Mixed Gothic-Alan-Hunnic groups may have arrived in Pannonia (Transdanubia) soon after 375[60]. The main body of Huns moved to the Carpathian Basin in the early 5th century, and probably settled around the power centre of the emergent Hunnic Empire in the Danube-Tisza-Interfluve. Admixture with Sarmatians, Alans or other Pontic groups would have been possible then. Unfortunately, genetic data for the Huns are still scarce, also because the archaeological traces of the Hun elite are rather rare in the Carpathian Basin[61,62]. Compared with the thousands of burials from the Sarmatian and Gepidic period, the graves that might be attributed to Huns are extremely sparse. Nine individuals from the period were recently analysed and some showed Central Asian ancestry[63]. The persistence of a substantial group with strong steppe ancestry in the Carpathian Basin over the following 120 years is therefore genetically, archaeologically and historically unlikely.
- Third, we have written evidence that in the second half of the 5th century, new groups from the east arrived in the Pontic steppe; an early report of such a movement is dated to 463 CE. In the 6th century, the presence of a number of Ogur-Turkic peoples is well-documented, among them Ogurs, Onogurs, Utigurs, Kutrigurs and Saragurs. The Bulgars, first attested along the Lower Danube in the late 5th century, are also closely related to this new milieu in the steppe[1,64]. We still lack genomic reference data from these populations, but can assume that they mixed with previous inhabitants of the Pontic steppes. Some of them followed the Avars into the Carpathian Basin (where Bulgars and Kutrigurs are attested). Instead, we have more genomic reference data across the first millennium CE from the Eastern Eurasian Steppe, specifically, from present-day Mongolia[65]. Here, while the majority of the individuals are shifted towards high amounts of East Asian ancestry, there is still a large and heterogeneous east-to-west Eurasian cline of genomic variability. We could speculate that this heterogeneity was also present further west in the Steppe during this period, plausibly with an overall decreasing amount of East Asian ancestry proceeding westwards. This would then be in agreement with this historical scenario.

European ancestry individuals and the smaller pedigrees of RK

In the RK site across the whole Avar period there is a minority (N = 36) of individuals that carry no detectable signs of East Asian admixture and instead are distributed along a gradient of European genomic profiles, in line with the variability observed so far in the Carpathian Basin in the preceding 5th - 6th centuries CE (Extended Data Fig. 8). As described above, only three of these are detected as exogamous mothers included in the main set of pedigrees depicted in Fig. 3. The others are either unrelated individuals or part of 3 smaller pedigrees not related to any other one. These are pedigrees 17, 20 and 21, part of an overall set of 22 related individuals forming 9 smaller pedigrees not reported in Fig. 3 (Supplementary Fig. 15). This set includes these latter 3 pedigrees of European ancestry individuals and 6 others composed of individuals carrying the more common admixed RK ancestry (Supplementary Fig. 16). Interestingly, while not linked by close genetic relatedness to the main RK pedigrees, pedigrees 10, 19 and 14 are still highly interconnected to them by more distant IBD links, fully intertwined inside the RK clusters of the IBD network (Fig. 3; Supplementary Table 4).

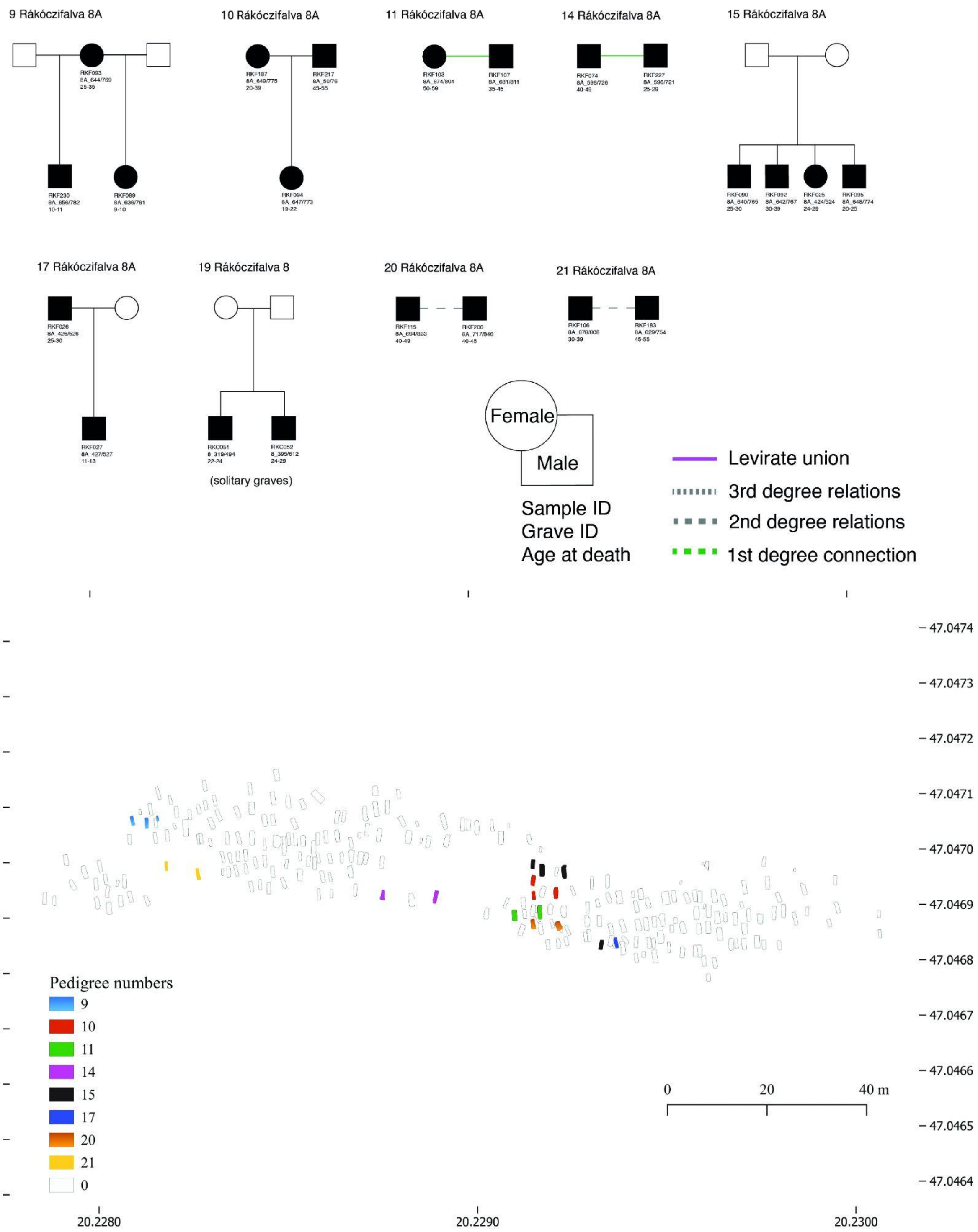

Supplementary Fig. 15 - The remaining 22 related Avar period individuals in RK, not shown in the main text, forming 9 smaller pedigrees (top) and their position in the cemetery map (bottom).

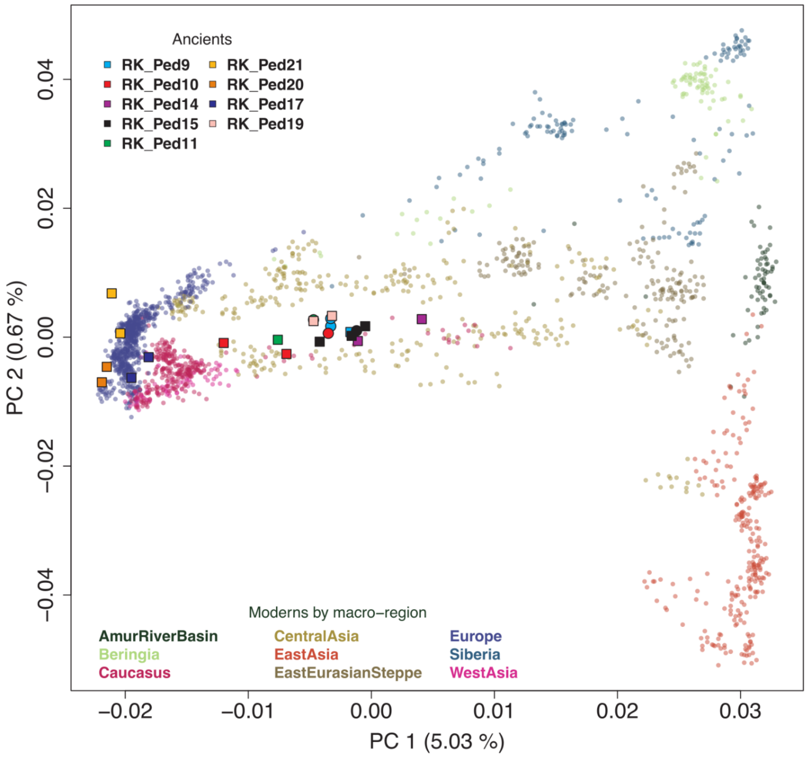

Supplementary Fig. 16 - Eurasian PCA showing the remaining 22 related Avar period individuals in RK that are not part of the main pedigrees shown in the main text. individuals are color coded according to the pedigrees in Supplementary Fig. 15.

Pedigree building strategy

To reconstruct the genealogical structure of the pedigrees, we started by considering the list of pairs of first-degree related individuals estimated by KIN[66]. For a given pair, KIN runs multiple models, each assuming a particular case of relatedness, and infers the best fitting model as the model with the maximum likelihood. It outputs the relatedness corresponding to the best fitting model along with the log of ratio of likelihoods of the two best fitting models (LogL). We took only the assignments with a LogL > 3 to avoid false classification mostly due to low coverage and therefore too low number of overlapping SNPs between the two individuals to securely infer their relatedness (Supplementary Table 4). KIN can also distinguish between first-degree parent-child or sibling relations. We used this information to start building the basic structure of every genealogy: biological father-mother and child/children siblings. Genetic sex is a key element that helps in this process, as only a biological male and female can have offspring together. Uniparental haplogroups were used as a sanity check as sons inherit the same Y-chromosome haplogroup of their father and all the children inherit the mtDNA haplogroup of their mother. Anthropologically determined age-at-death is another key information in pedigree building. It can serve both as a sanity check and, in some instances, it is crucial to determine the directionality of a parent-child or grandparent-grandchild relation: when one of the individuals is an

infant or a child (below 10-12 years of age) it can only be the descendent and not the progenitor. Following this procedure, we were then able to solve most of the genealogical structure of the 31 pedigrees reconstructed in this study.

Within the KUP site, among the 14 EA period individuals, 7 were related in forming two distinct pedigrees and among the 14 LA period, 9 were related in 3 distinct pedigrees (Extended Data Fig. 2). Within the MA and LA period KFJ site, among the 58 individuals, 44 were related in one single pedigree. In the MA period site of HNJ among the 18 individuals, 10 were related in one pedigree and the other 8 were unrelated or part of a smaller pedigree (Extended Data Fig. 1). Finally, within the biggest Avar period site of RK that we analyzed, chronologically spanning the whole Avar period (late 6th to early 9th century CE), among 281 individuals, 202 were related, 3 pairs were genetically identical, 14 were too low coverage (poorly preserved) to assess relatedness and 62 were unrelated to anyone (Supplementary Table 1). Among them 146 formed an extended "macro" pedigree that for practical reasons we numbered it divided into five connected pedigrees 1 to 5 and another 34 individuals formed 3 additional multi generation pedigrees 6, 7, 8 and 12 (Fig. 2; Supplementary Fig. 8). The remaining 22 individuals formed 9 smaller pedigrees (Supplementary Fig. 15). Among the 48 individuals from three RK Sarmatian period cemeteries 19 resulted to be close relatives (including a pair of identical) forming 3 pedigrees and we found a pair of brothers among the 8 Gepid period burials (Supplementary Fig. 6).

We found a total of 9 pairs of genetically identical individuals, for which the most parsimonious explanation is that they are the result of double sampling of the same individual (Supplementary Table 1).

Special cases in pedigree building process

The only inconsistencies (not due to low-coverage and LogL < 3) that we found among the first-degree relatedness estimates involved the pairs of children from levirate unions. In these cases, the individuals are genetically classified as siblings, they share the same mother but are also classified in another parent-child relation with two different biological fathers (Supplementary Table 4). However, we found in all these cases that the two different fathers were themselves classified as being first-degree related (either parent-child or siblings). By having a different but first-degree related father, the children of levirate unions are technically half-siblings but share more DNA in common than the more frequent case of half-siblings, where the two parents not in common are unrelated and therefore the children fall within the second degree variation (Methods).

With this assumption we were able to solve these apparent inconsistencies and build into the pedigree the three first degree levirate unions in RK (the other 2 second degree levirate in RK do not pose this issue) and the one in KFJ (Fig. 2; Extended Data Fig. 1). There is a difference for the presumed levirate union inferred for pedigree 1 of KUP. In this case, all the individuals involved in the levirate union are missing. Here we found a different type of inconsistency, out of three siblings (KUP027, A1820,

A1822/KUP005), only one (KUP027) showed a second-degree relation with A1817. The other two siblings were unrelated to this individual (Supplementary Table 4). Unrelated classification could still be consistent with more distant relatedness (3rd degree or more), but second degree or closer can be excluded for these pairs since they plot outside the distribution of the second-degree simulated data, sharing less IBD than what is expected by different types of second degree relatives (avuncular, grandparent-grandchild and half-siblings; Supplementary Fig. 17).

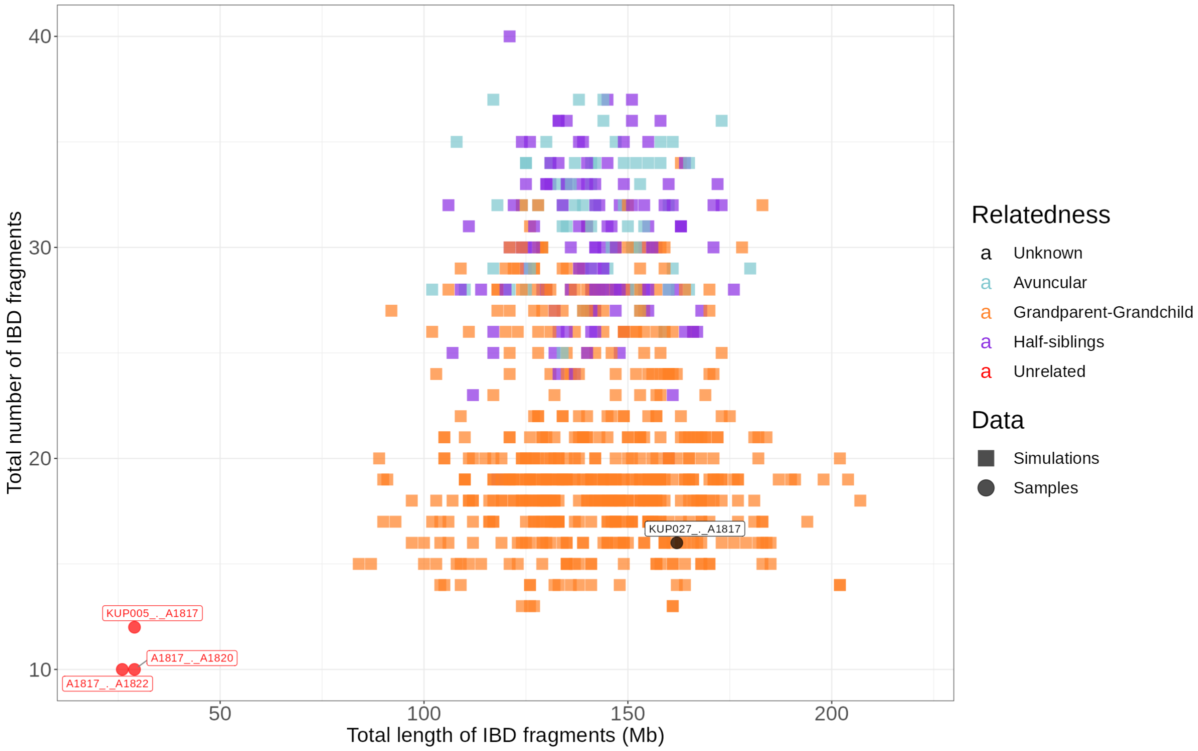

Supplementary Fig. 17 - Plot showing that A1822, A1820, KUP005 are not up to second-degree related with A1817. Simulated pairs of individuals are plotted as squares with different colors representing cases of relatedness, while pairs from this study are plotted as circles.

This type of relatedness configuration would only be consistent with A1817 being a direct descendant only of KUP027, so its grandchild, therefore separating A1817 by one extra degree of relatedness to A1820 and A1822/KUP005 (Extended Data Fig. 2; KUP 1, configuration 1). However, KUP027 (and also A1817) is an infant and it cannot have been a grandparent. We were able to solve this inconsistency only by assuming the three siblings to be a result of a levirate union where KUP027 is technically a half-sibling of the other two full siblings (consistent with all three sharing the same mother having the same mtDNA lineage) with different but first-degree related fathers. We know from the cases described above that in that case they would be still classified as full siblings. Since we are missing all the parents, we cannot choose between the related fathers being siblings or parent-child, both are equally consistent with the data and both would solve the inconsistency posed by the age-at-death as the infants, KUP027

and A1817, are modeled as offspring and not as parents or grandparents (Extended Data Fig. 2; KUP 1 configuration 2 and 3).

We built in the genealogical structure of the pedigrees only the individuals we could securely model into one possible configuration (according to the rationale described above). When we couldn't place the individuals inside the pedigree because multiple configurations were equally possible, we graphically represented the relatedness estimates with different dashed or dotted connecting lines depending on the degree of relatedness estimated by KIN (first-, second-, third- degree; Fig. 2; Extended Data Fig. 1-3). For simplicity, we made exceptions to this and instead decided to choose one between different possible configurations and draw it in the pedigrees in 3 different cases where we had some contextual or logical reasoning to select one of the possible options as more reasonable (Supplementary Fig. 10). These are outlined below.

First, pedigree 7 posed a challenge because of two different missing first-degree links (Supplementary Fig. 10). One involving a second degree between RKF080 and three full siblings (RKF178, RKF186 and RKF060) and with their father RKF076. The only possible configuration that is not inconsistent with the other relations of pedigree 7 would make RKF080 the son of another missing sibling of RKF178, RKF186 and RKF060, so RKF076 would be the grandparent. The distribution of the IBD values of the pairs involving these individuals and RKF080 confirm the directionality as the pair RKF076_RKF080 falls within the distribution of grandparent-grandchild making the alternative scenario less plausible (Supplementary Fig. 18). The second missing first degree link involves other second degree relations between RKF080 and RKF220, RKF234, RKF228. RKF228 is a 5-6 year old child with a parent-child relation with the female adult RKF084 (therefore the mother) who in turn is unrelated to all the other individuals mentioned. In this case, there is uncertainty with different configurations possible but the distribution of all the second-degree relations of RKF220, RKF234, RKF228 with RKF080 show the same distribution at the extreme of the avuncular cloud (Supplementary Fig. 18) while the pairwise relations between RKF220, RKF234, RKF228 are closer to the center of the cloud (more towards the grandparent-grandchild and other half-siblings). Therefore, we modeled RKF220, RKF234, RKF228 as niece and nephews of RKF080, children of his missing brother (because only one mother is possible so it cannot be a missing sister) with three different female partners, which would make them all half-siblings between each other consistent with these trends in the data. However, uncertainty and overlap between the distribution of different categories of second-degrees makes it impossible to rule out completely other possibilities.

The second case is the second degree relation between the two males RKF180 and RKF182 at the top of pedigree 2. The IBD distribution plots in the middle of the cloud making it impossible based on this alone to decide what type of second-degree relation it could be (Supplementary Fig. 18). RKF180 is in a parent-child relation with the adult female RKF061, whose relation with the progeny of RKF180 is consistent with her being the mother of RKF180, which would place her as the only member of the very first generation of pedigree 2 consistent with the very early archaeological chronological date for this

individual. Therefore RKF180 and RKF182 don't share the same mother. This excludes some options but still multiple second degree options remain possible. However, chronologically the two individuals and their descendants are consistent with being roughly contemporaneous and they both had offspring with the same female partner (RKF185; making this a second-degree levirate union). Therefore, the grandparent-grandchild relation seems less likely given the time interval between a grandparent and grandchild. Furthermore, although this cannot be accessed with certainty, their distribution in the IBD plot would suggest rather a half-siblings relation than avuncular (Supplementary Fig. 18). For these reasons and for the sake of simplicity, we decided to draw the half-siblings option also considering that the other possibilities would anyway not alter the interpretations about social structure. RKF180 and RKF182 are in any case two males and close relatives (either uncle-nephew or grandparent-grandchild or half-siblings) that had offspring with the same female individual.

The last uncertainty involves the second degree relation between RKC021 (pedigree 2) and RKC039 (pedigree 3), which is at the center of the Y-chromosome male line shift described in the main text. They have different Y-haplogroups (J1a and J2b) and the same mtDNA haplogroup, for this reason we know they are related via the maternal line. The IBD position of this pair is at the extreme end of avuncular distribution thus excluding a grandparent-grandchild option (Supplementary Fig. 18). Here, again for simplicity, we choose one of the two remaining options (half-siblings or avuncular) on the basis of the overall chronology of pedigree 2 with respect to pedigree 3 that would be more consistent with them being half-siblings through a missing common mother although them being avuncular via maternal line (through a missing sister) is still possible. Again, regardless of their exact relation, the interpretation doesn't change.

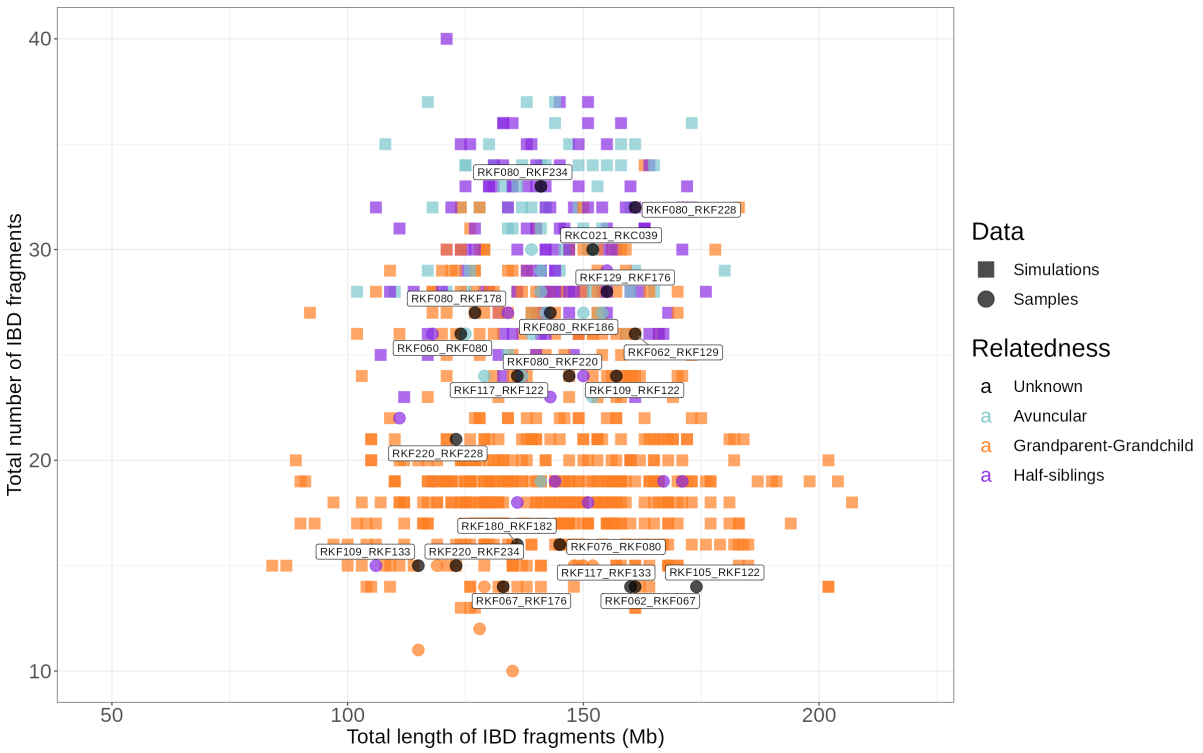

Supplementary Fig. 18 - Plot showing the distribution of the total number and length of IBD for second-degree relatives. Simulated pairs are plotted for reference as squares, and different colors represent cases of relatedness. Pairs from this study are plotted as circles for comparison.

Genetic relatedness validation with ancIBD, KIN and PMR

First, we compared the sum of IBD vs the number of IBD we estimated on our new data to a simulated pedigree dataset on which ancIBD was run with the same parameters as we did (Supplementary Fig. 19-20). The simulations were taken from Ringbauer et al. 2023. According to Caballero et al. 2019 estimates, successful detection of IBDs can extend up to 6th degree which is the maximum degree that was simulated in Ringbauer et al, 2023. From the relationships built in the pedigrees we were able to report up to 8th degree relatives. From this comparison it is possible to appreciate a striking match between the inferred relations from the pedigrees and the expected distribution of IBDs from the simulations. It is also possible to observe that there is no enrichment for low numbers of IBDs in the real data compared to the simulations also for higher degree relatives (3rd to 8th), showing that the different recombination rates in males and females described in Caballero et al 2019 do not have a detectable effect on the IBD sharing patterns.

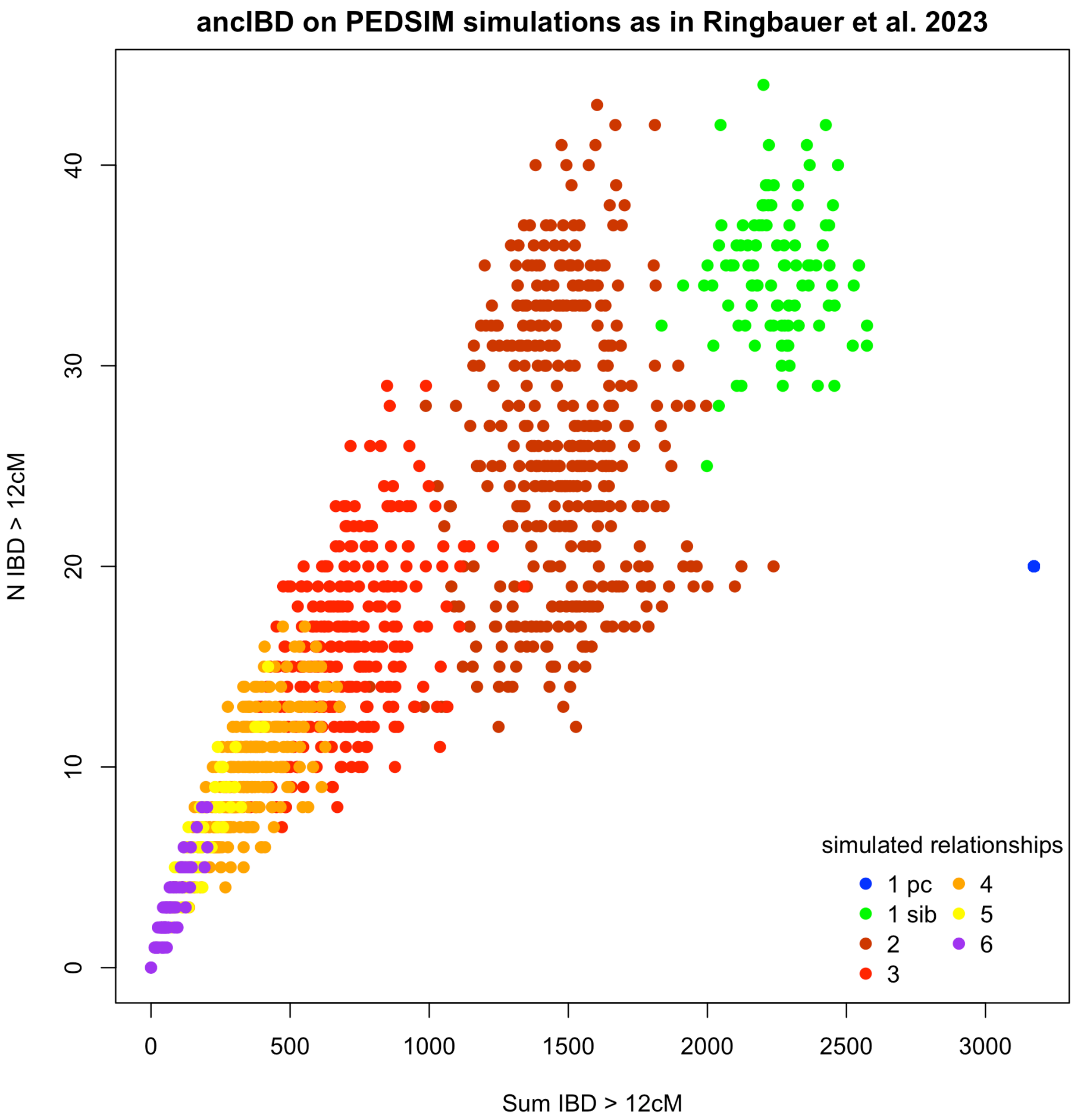

Supplementary Fig. 19 - Distribution of IBD sharing from ancIBD estimates on simulated pedigrees. Degrees of relationships are known from simulations.

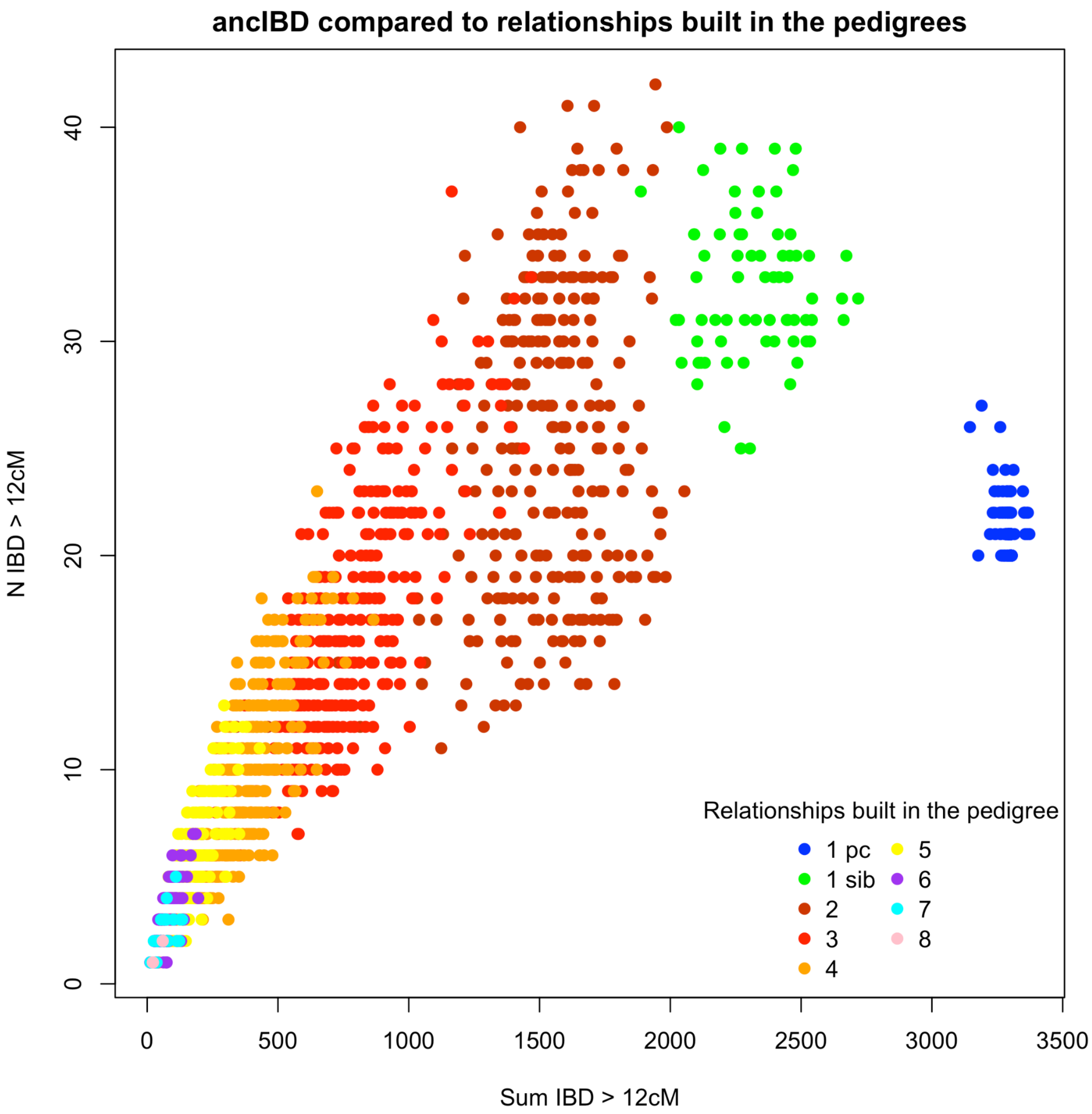

Supplementary Fig. 20 - Distribution of IBD sharing from ancIBD estimates on our new data (all 4 sites combined). Degrees of relationship show are the ones inferred from the pedigrees.

We then compared the close genetic 1st and 2nd degrees estimates from KIN that were used to build the pedigrees with the IBD sharing patterns we inferred with ancIBD (Supplementary Fig. 21). It is possible to appreciate the high correspondence between the two methods.

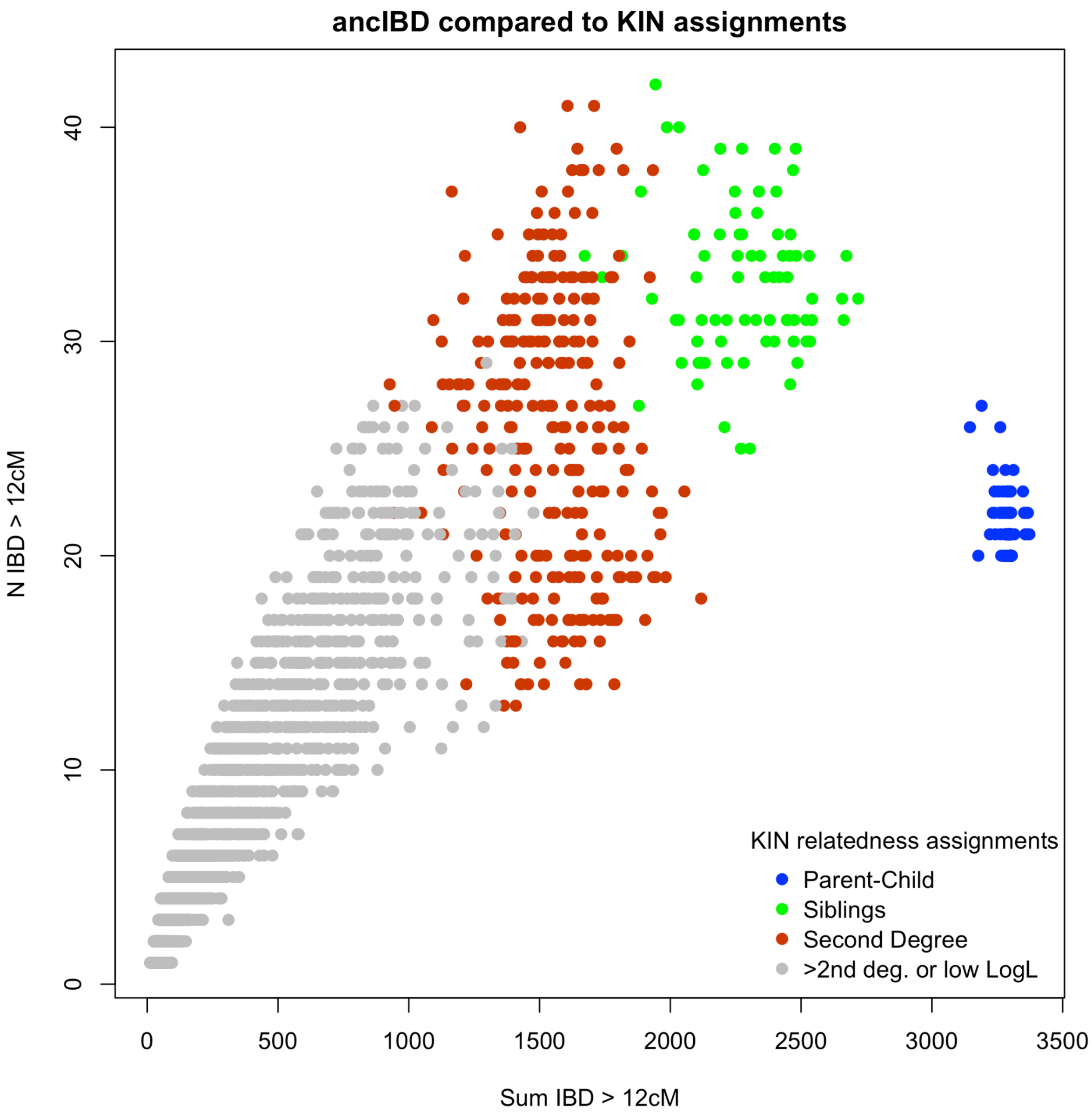

Supplementary Fig. 21 - Distribution of IBD sharing from ancIBD estimates on our new data (all 4 sites combined). Degrees of relationship shown are the ones estimated by KIN (Supplementary Table 4).

Given the higher recombination rates of females with respect to males (Caballero et al. 2019), correct IBD sharing patterns should be able to distinguish for certain types of relationships if they are through female or male line. For example, maternal or paternal half-siblings are among the ones whose IBD sharing most differs (Caballero et al. 2019). To test whether we could appreciate this pattern from the IBD sharing of our reconstructed half-siblings from the pedigree we plotted them on the IBD distribution plot (Supplementary Fig. 22). Indeed we found that maternal half-siblings have a systematically higher number of IBDs (i.e. they are more broken down by higher recombination rates) with respect to paternal half-siblings. We then looked at paternal and maternal grandparents/grandchild.

Given the strict patrilineality we found only one case of maternal grandparent/grandchild in the site of RK (see main text). It is still striking to observe that this single case is indeed showing a higher number of IBDs with respect to all the other paternal grandparent/grandchild relations, as expected (Supplementary Fig. 22).

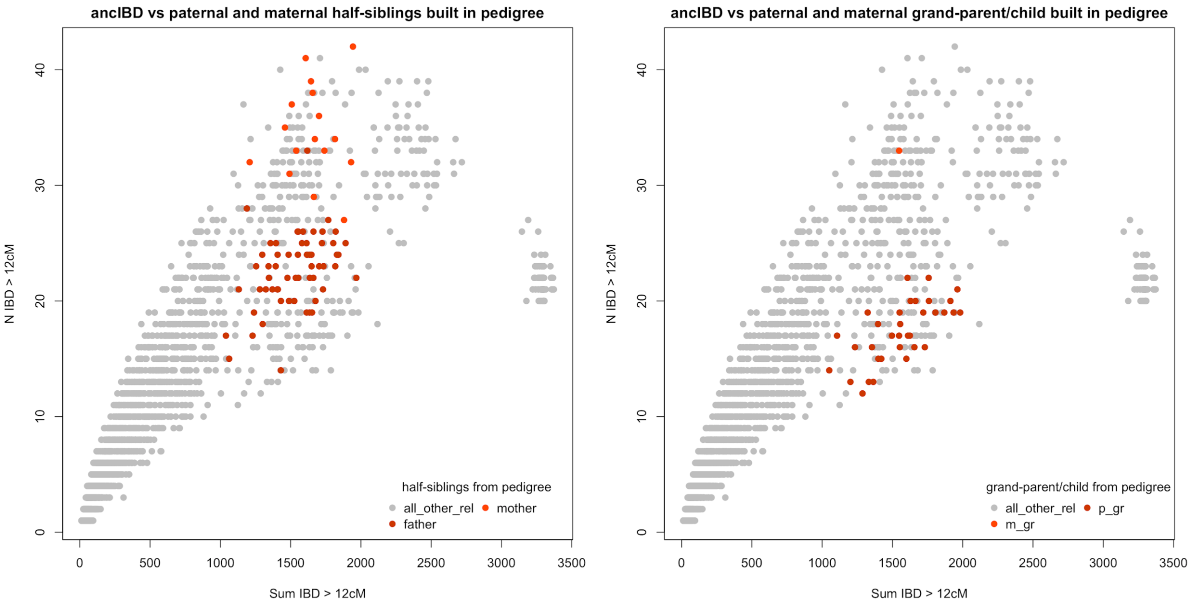

Supplementary Fig. 22 - Distribution of IBD sharing from ancIBD estimates on our new data (all 4 sites combined). On the left: maternal and paternal half-siblings are inferred from the pedigrees. On the right: maternal and paternal grandparents/grandchild are inferred from the pedigree.

We further validated the genetic relatedness estimates of KIN with another widely used statistics for estimating relatedness with ancient DNA genomic data: the pairwise mismatch rate (PMR) (Supplementary Table 4). This validation allows us to include also the individuals without enough coverage for the ancIBD analyses (Methods). A commonly used software that assigns relatedness based on PMR is READ[67]. However, we found it not computationally capable of handling the large amount of pairwise tests (individuals) that our dataset requires to test altogether. Therefore, we used a recently developed R package called BREAD (https://github.com/jonotuke/BREADR) that uses a very similar principle as READ to assign genetic relatedness based on PMR but is computationally more efficient and able to handle the size of our dataset.

With PMR it is possible to derive the standard *r* coefficient of relatedness, through the formula adapted as in reference Jeong et al. 2018[68] first described in reference[69]. Theoretically, r=1 corresponds to identical/twins, r=0.5 first degrees and r=0.25 second degrees. In reality, the stochastic nature of how much DNA is inherited from a common ancestor makes the observed values vary considerably between these boundaries, but it is still a useful and straightforward measure that can help explain discordances detailed below. Being based on basic PMR statistic, these tools don't have the same resolution as KIN[66]

(that instead models the pattern of relatedness along the haploid genome) to distinguish the different within-degree relations (i.e. first degrees: parent-child vs siblings; second degrees: avuncular vs grand-parent/child). For this reason we didn't base the pedigree-building on these results. On the other hand PMR carries less assumptions than KIN[66], so it could serve as a good validation tool for the KIN estimates.

We found that KIN and BREAD results for first and second degree classifications used for pedigree reconstruction match at a 95.8% rate (overall N=738 overlapping comparisons). Of the remaining ~4.2% that don't match (N=30), four are cases where KIN estimates a first degree and BREAD a second degree. These are all levirate pairs with *r* coefficients around 0.3, so quite low for first degree relations (Supplementary Table 4). This is an interesting finding although not unexpected, as levirates are between degrees. They are half-siblings that share more DNA (see above) but still should share less than full-siblings. Similarly to standard half-siblings, how much DNA they inherit in common from the parents is stochastic and it is expected that it can vary considerably (Supplementary Fig. 17 and 18). Furthermore, contrary to the standard half-siblings, this stochasticity applies for both parents and not just the one in common (as the different parents are first degree related thus also sharing a great proportion of DNA in common). So it is not surprising that this variability could be even larger, possibly resulting in cases where the amount of shared DNA falls within the full-sibling range and other cases where it falls within the standard half-sibling one. In conclusion, none of the tools available are fully able to identify levirate unions, only thanks to the amounts of related individuals that we identified in our dataset, we were able to build them in the pedigrees. A future line of improvement for genetic relatedness methods is to explore in more depth the patterns of this and other "in-between" relatedness states to provide higher resolution classifications.

The remaining 26 mismatches are all cases where BREAD estimates first degree and KIN second degree relations. Almost all involve individuals within the KFJ pedigree (Extended Data Fig. 1) and one pair part of the HNJ pedigree 1 (Extended Data Fig. 3). However, for all of these pairs a first degree relation is inconsistent with their other relationships to other samples. For example: the two males HNJ012 and HNJ016 are first degree according to BREAD (although the *r* coefficient is 0.38). Although, both of them are also in a parent-child relation with male HNJ011 which is inconsistent with any pedigree configuration where HNJ012 and HNJ016 would be first degree as well. Instead, their being second-degree grand-parent/child is consistent with their relation to HNJ011 and all the other relations around them (Extended Data Fig. 3). Similar scenarios apply to the 25 KFJ pairs. As all of them have *r* coefficient < 0.5, we interpret that these might be due to background relatedness in KFJ being slightly higher (i.e. lower PMR average/baseline), this would affect the *r* coefficient calculation. To test this hypothesis, we recalculated the *r* coefficient of KFJ using only the within KFJ average PMR, which is in fact lower than the total average (0.31 compared to 0.34). With the new baseline, BREAD classifies all the previous 25 first degrees as second degrees in line with KIN results. Still, six different mismatches result from this new analysis, bringing the overall match rate up to 98.6% (Supplementary

Table 4). All of them are cases where BREAD estimates a second degree and KIN a first degree. Of these, three are levirate sibling pairs for which is valid the same logic as for the RK cases described above. The other three are cases where the BREAD estimates are inconsistent with their other relationships to other samples.

In conclusion, the overall agreement between BREAD and KIN is very high and it's also supported by the ancIBD results (for the subset of the individuals that have enough coverage for IBDs and that we could compare, see Methods), which confirms the relatedness estimates used to build the pedigrees (Supplementary Table 4). The low proportion of mismatches are either levirate pairs connected to the problem of classifying the in-between degree relatedness states described above, or a handful of cases with borderline *r* coefficient values where the KIN classification appears the one supported by other related pairs around. It is thus reasonable that these are few cases where KIN, based on tests of formal relatedness models measuring pattern of DNA sharing along the genome instead of relying only on the overall PMR values, is able to capture the correct relatedness state.

Strontium isotope data of the burials of Kunpeszér, Kunszállás and Rákóczifalva

A total of 76 $^{87}Sr/^{86}Sr$ measurements were carried out on tooth enamel of burials from the Kunpeszér-Felsőpeszéri út and Kunszállás-Fülöpjakab sites (Supplementary Fig. 23; Supplementary Table 3). From the former site, 23 samples were taken from 23 individuals, the minimum value was 0.70913 (Grave 19), and the maximum value was 0.71138 (Grave 18). At Kunszállás, 53 teeth samples were taken from 50 individuals, the minimum value was 0.70910 (Grave 61), and the maximum value was 0.71095 (Grave 38). Two teeth each were sampled from Graves 12 (Inf. II), 18 (Inf. II) and 25 (Inf. I). Among them, Grave 18 yielded the most remarkable results, with an isotopic difference between its two molars (46 and 47) of almost 0.0003. In contrast, the $^{87}Sr/^{86}Sr$ ratios of both teeth of the other two children were almost identical. All three individuals are lineage children (boys). The means at Kunpeszér and Kunszállás were 0.70955 and 0.70953 respectively, and the medians were both 0.70948. When comparing the two sets of samples statistically, normality tests were performed, which revealed that neither of them had a normal distribution, while the Kolmogorov-Smirnov test of the distributions showed that they were equally distributed (D: 0.10911, p(same dist.): 0.98643). By performing the Mann-Whitney test, it was evident that the median of the two sets of samples was also equal (U: 602, p(same med.): 0.93691), so there was no significant difference between the data sets of the two sites in this respect. Moreover, the two sets of samples were subjected to the Grubbs outlier test (Kunpeszér: G: 4.295, p(no outlier): 0; Kunszállás: G: 4.493, p(no outlier): 0), which revealed that the graves with the highest values in both cemeteries were considered outliers.

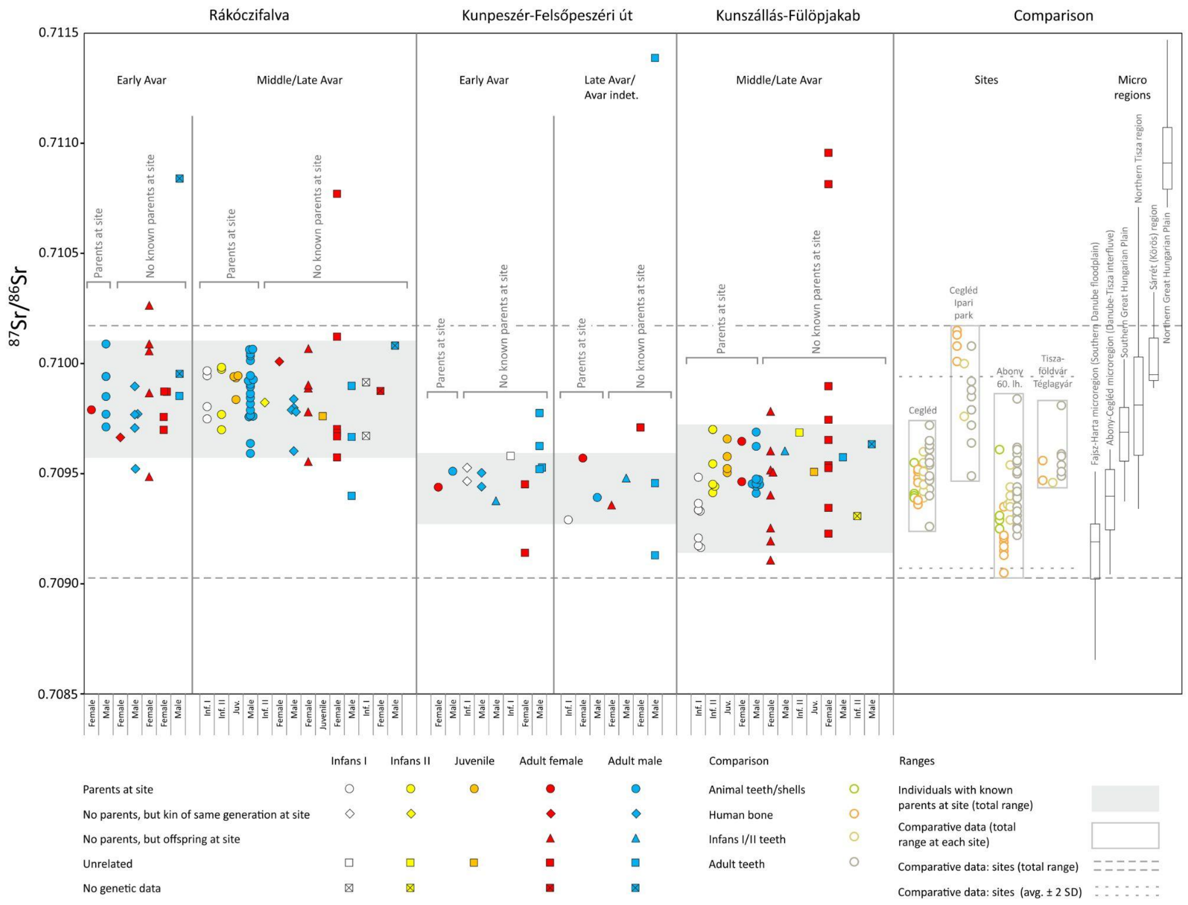

Supplementary Fig. 23 - $^{87}Sr/^{86}Sr$ ratios of the enamel samples of the Avar-period burials of Kunpeszér, Kunszállás and Rákóczifalva (left). The symbols reflect the age of subadult individuals, the sex of adult individuals and information on genetic relations based on the pedigrees. For the three subadults with two sampled teeth, only one value is included. The right side comprises comparative data, including data that reflect the bioavailable $^{87}Sr/^{86}Sr$ values as well as data of human teeth at Neolithic sites in the same area as the cemeteries. Boxplots represent summary data for microregions of the Great Hungarian Plain area based on reference[70,71]. The boxes and whiskers are redrawn based on Depaertmentier et al. 2021, Fig. 4[70] and represent the micro regions e, j, g, i, k, and h. All n >10.

In the case of Kunpeszér, 4 children (Inf. I), 7 females (Adultus 1-2 - Senilis) and 12 males (Adultus 1-2 - Senilis) were sampled, whereas the dataset from Kunszállás comprised 17 children (8 Inf. I - 9 Inf. II), 23 females (Juvenis - Senilis) and 13 males (Adultus 1-2 - Senilis). The above groups showed a mixed normal and non-normal distribution, so comparisons within and between all sites were made by comparing medians for all but the four children from Kunpeszér. The results of the Mann-Whitney paired tests showed that there were no significant differences between the medians of the majority of the groups, only Inf. I child samples from Kunszállás show a significantly lower median than the rest of the samples with the exception of Kunpeszér females (Kunpeszér females: U: 13, p(sam med.): 0.09334; Kunpeszér males: U: 14, p(same med.): 0.00975; Kunszállás Inf. II: U: 10, p(same med.):

0.01408; Kunszállás females: U: 29, p(same med.): 0.004775; Kunszállás males: U: 7, p(same med.): 0.00127). Remarkably, when the Bonferroni correction is taken into account, only Kunszállás males remain significantly different (corrected p(same med.): 0.01905).

Significantly more graves were selected from the Rákóczifalva-Bagi földek 8 and 8A sites, 138 in total (Supplementary Fig. 23). These included a mixture of Sarmatian, Hun, Gepid and Avar period burials, but in this study, we focused exclusively on the population of the Avar period. The lowest value of the 82 teeth sampled was 0.7094 (Grave 147/232), and the highest was 0.71084 (Grave 421/521). All data yielded a mean of 0.70986 and a median of 0.70985. The data set was also subjected to the Grubbs outlier test (G: 4.49; p(no outlier): 0.000127), which revealed that the male grave 421/521 (0.71084) and the female grave 47/78 (0.71077) were outliers, due to their radiogenic values. One of the Inf. I. children (grave 288/443) was sampled twice, but the difference between the two results coming from molar 75 and molar 85 was minimal (0,00002). The first step in the analysis was the approximation of the local value, using the results of the children (12 Inf. I-II individuals).

When comparing females (27 Juvenis - Maturus 1-2) and males (43 Juvenis - Maturus 1-2), we again found a non-normal distribution, so we compared first the distributions (Kolmogorov-Smirnov: D: 0.15676, p(same dist.): 0.77404) and then the medians (Mann-Whitney: U: 579, p(same med.): 0.99037). The results were negative in both cases, with no significant difference between the two groups, and negative when the same tests were performed in pairs and children were included.

The Rákóczifalva data set also provided the opportunity to investigate other aspects, firstly the internal chronology of the cemetery. For this purpose, we included the graves that could be dated, with 20 early, 12 middle and 29 late Avar grave samples. Using the same normality test (Shapiro-Wilk) it revealed that the distributions were normal, so the results of ANOVA show that there is no significant difference between the variance (Levene's test: p(same var.): 0.4004) and mean (df: 2:58, F: 0.2182, p(same mean): 0.8046) of the graves classified in the different periods. An attempt was also made to compare the 'founders' of the genealogical trees, pedigrees, by comparing the results of 9 individuals with the rest of the population. This time, the normal distribution prerequisite was not met, so the results of Mann-Whitney test were again negative, with the selected samples not significantly different from the rest of the community (U: 318.5, p(same med.): 0.88788). Our last criterion was the classification by so-called status objects, for which we were able to consider 18 individuals. Without having normal distributions and comparing the medians with Mann-Whitney test, we again found no significant difference (U: 517, p(same med.): 0.51204).

Incorporating information on biological relatedness based on the pedigrees, we found that individuals whose parents were identified at the respective cemeteries (i.e., who belonged to a second or a later generation) had generally more homogeneous $^{87}Sr/^{86}Sr$ ratios than individuals without identified parents, supporting the hypotheses of having grown up locally. This group included most subadult individuals, who similar to previous studies[72,73], yielded less variable $^{87}Sr/^{86}Sr$ ratios than the adults.

This finding supports that Sr isotope ratios of enamel of subadult individuals provide a good estimation of the isotopic composition of the local biologically available strontium. Individuals whose parents were not identified among the individuals of the investigated burial communities were potentially more likely to have grown up in a distant location. Among them, we differentiated individuals with close relatives of in most cases the same generation (mainly siblings), individuals with identified offspring (most of them exogamous females), unrelated individuals (some of them possibly mothers of offspring/daughters who moved into other communities) and individuals without genetic information. We find that unrelated individuals and individuals who had offspring but no parents at the burial communities, yielded more variable $^{87}Sr/^{86}Sr$ ratios in their teeth than those whose parents were also buried at the respective sites. Indeed, all individuals with more radiogenic $^{87}Sr/^{86}Sr$ ratios than typical for the region where the sites are located (Supplementary Fig. 23), belonged to one of these groups. In addition, each burial community included few potential first-generation non-locals (based on the absence of identified parents) whose $^{87}Sr/^{86}Sr$ ratios were slightly more or less radiogenic than the strontium found among the individuals with identified parents, but still within the regional range. Nevertheless, and even more remarkable, at all sites, the Sr isotope data of the majority of the individuals without identified parents yielded $^{87}Sr/^{86}Sr$ ratios that match those of the individuals whose parents were identified. If they originated from a distant community, it was isotopically indistinguishable. The finding of genetic first-generation arrivals that have $^{87}Sr/^{86}Sr$ ratios within the local and regional ranges is in agreement with the results of the network analysis, which provided evidence for direct connections among the communities in the Carpathian Basin during the Avar period. Yet, individual instances of residential changes over larger distances can also not be excluded as the $^{87}Sr/^{86}Sr$ ratios of a region are not unique to it. However, the homogeneity of the datasets of the Avar-period cemeteries is striking, as e.g., at sites of the 5th and 6th century in the Transdanubian part of the Carpathian Basin, we see more isotopic variation[72,73].

Compared with the most recent bioavailable $^{87}Sr/^{86}Sr$ data from the Carpathian Basin, including Hungary, (Supplementary Fig. 23)[70], it is clear that the Kunpeszér and Kunszállás Inf. I group, the two sets of samples fit perfectly into the Abony-Cegléd micro-region, in a broader sense the Danube-Tisza Interfluve region. Some of the slightly higher values shown by the adults also fit well into this region, overlapping more broadly with the values observed in the Southern Great Hungarian Plain and the Upper Tisza region. The three graves with outstanding values, namely Kunpeszér 18, Kunszállás 36 and 38, can be considered extra local, i.e. non-local, and the closest geographically similar values are found in the foothills of the North Hungarian Range. Grave 18 (KUP014) with no grave goods fits the LA period community of Kunpeszér of 100% local ancestry. Regarding the outliers from Kunszállás, both MA or early LA, Grave 36 (KFJ032) is a 28-36 year-old unrelated female with a pair of gilded silver earrings, who demonstrates 100% Eastern steppe ancestry (like early Avar DTI elites). Grave 38 (KFJ034) is a 43-60 year old unrelated female buried with a pair of gold earrings, spindle whorl, iron

buckle, and animal bones, who represents more admixed ancestry than the rest of KFJ with a higher Northern Caucasus component. The relative wealth of the artifacts suggests that these women came to this community as wives, but we cannot trace their relatedness through children.

The mean of the Inf. I-II samples from Rákóczifalva is 0.004 higher than the means at the other sites, and the reconstructed local value also fits well with the wider northern Great Hungarian Plain regions (Northern Tisza region). However, we do not see a shift between children and adults in this case. Individuals with $^{87}Sr/^{86}Sr$ values different from the local value exhibited both less or more radiogenic values. In the former case, they may have come to the Rákóczifalva area from the aforementioned Danube-Tisza Interfluve area. The Graves 421/521 and 47/78, exhibited values above the local range and included an outlier value. Overall, it did not show a higher value than the sample from Grave 18 in Kunpeszér, but can be placed next to Graves 36 and 38 in Kunszállás. Both Rákóczifalva outliers deserve attention from an archaeological point of view too: 421/521 (RKF022, Grave 36) is a 35-45-year old male with a Martynivka-type belt set, one of the earliest graves of the cemetery (564-597 cal AD unmodeled), from the time when Avars settled in the Carpathian Basin. This individual is a potential new immigrant from the East. Unfortunately, RKF022 was not suitable for a deeper genetic analysis. 47/78 (RKC004, Grave 8) is a 45-55 year old female from a very small and shallow grave-pit, without any grave goods, next to a young, late Avar female's (RKC013) grave of pedigree 3. This individual demonstrates a significant genetic divergence from the Rákóczifalva group, with Northern European ancestry. In this case, non-local origin and different genetic background may have been associated with lower status.

Carbon and nitrogen isotope data

Dataset and collagen quality

Carbon ($\delta^{13}C$) and nitrogen ($\delta^{15}N$) isotope analyses were carried out on bone collagen of 113 individuals of the Avar period from the site of Rákóczifalva 8 and 8A. The samples included 36 adult females, 57 adult males and 1 adult individual of indeterminate sex. Nineteen individuals were of subadult age, including the groups infans I (n=7), infans II (n=6) and juvenile (n=6).

The collagen was overall very well preserved. The samples contained on average 18.1 ± 4.4 % of collagen (min: 5.8; max: 28.7 %) with C contents of between 36.6 and 48.8 % (average: 43.0 ± 1.3 %), N contents of between 13.5 and 18,3 (average 15,8 ± 0.5 %) and atomic C/N ratios of between 3.07 and 3.22 (average: 3,16 ± 0.04). All samples fulfilled the criteria of well-preserved collagen and all isotope data were included in data analysis[74].

General characteristics of the human diet

The $\delta^{13}C$ values of the total dataset of the Avar period varied between -18.71 ‰ (juv., late Avar period) and -14.33 ‰ (adult female, Early Avar), whereas $\delta^{15}N$ values ranged from 9.82 ‰ (adult male, middle/late Avar) to 13.53 ‰ (adult male, Middle Avar) (Supplementary Fig. 24).

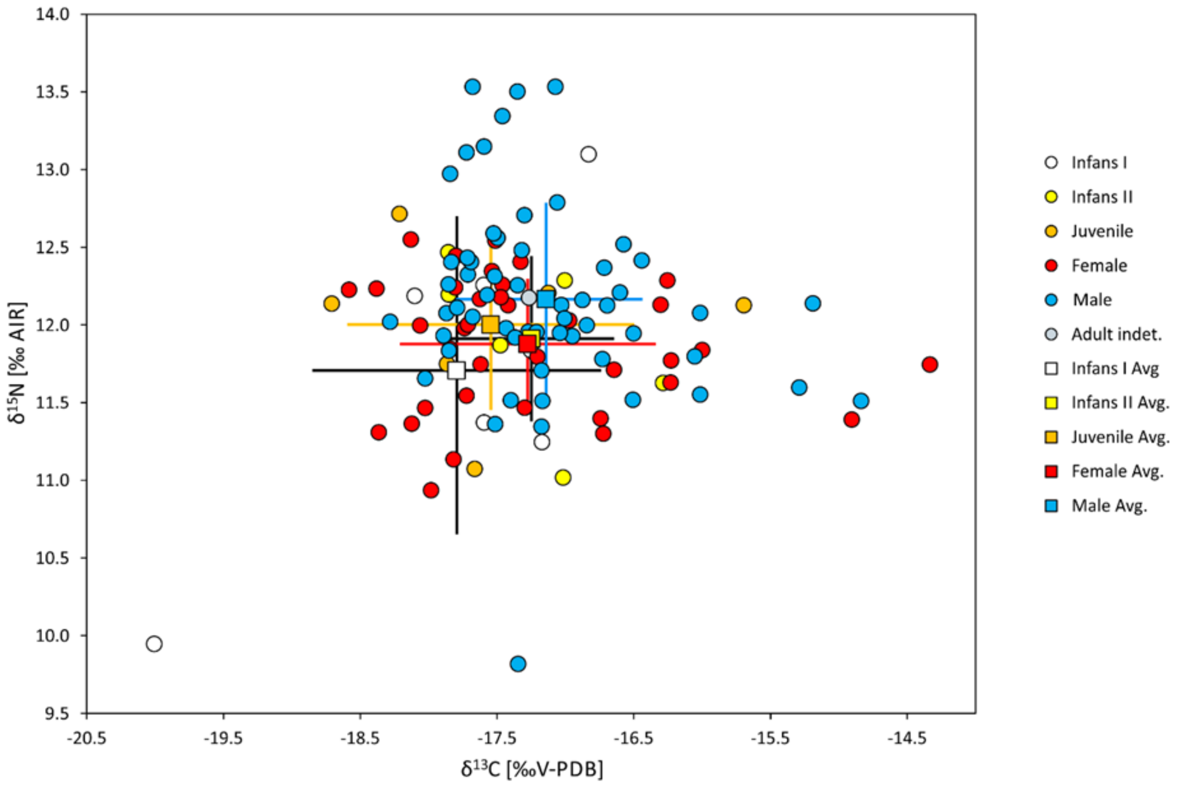

Supplementary Fig. 24 - Scatter plot of $\delta^{13}C$ and $\delta^{15}N$ values of bone collagen from the burial community of Rákóczifalva 8 and 8A. Adult individuals are grouped according to genetic sex, subadult individuals according to age groups. The error bars reflect 1 SD from the averages of the representatives of the age groups infans I (n=7), infans II (n=6), juvenile (n=6), adult female (n=36), and adult male (n=57) also reported in Supplementary Table 8.

In general, the photosynthetic pathways of the plants at the bottom of the food webs have the largest input on the $\delta^{13}C$ values of human bone collagen, going back to either the plant-based foodstuffs or the forage of the animals from which meat and dairy products were derived. The characteristic staple crops of central Europe, including cereals, such as wheat or barley, as well as vegetables and fruit are C_3 plants. Globally, their $\delta^{13}C$ values average at -26 to -27 ‰ V-PDB with forest cover, aridity and humidity, as well as temperature leading to variation[75,76]. Carbon isotope fractionation between plants and collagen of their primary consumers is about 5 ‰, resulting in $\delta^{13}C$ values of bone collagen of C_3-plant-eaters of -22 to -21 ‰. Among the samples from Rákóczifalva, the 4–5 year old child 579/700

(Site 8A; Szakálhát, Neolithic period), who was sampled accidentally and yielded the lowest $\delta^{13}C$ value of the entire data set and appeared to be an outlier, is the only analytical value in that range. These results are in good accordance with the Neolithic data from the Great Hungarian Plain[77]. The Avar-period individuals have higher $\delta^{13}C$ values, starting with -18.71 ‰ (juvenile, female, late Avar), which points to the contribution of a dietary component with higher $\delta^{13}C$ values. Among the C_3 plants, hot and dry environmental conditions have been observed to lead to higher $\delta^{13}C$ values (up to -22 ‰)[75,76], and non-photosynthetic parts, such as roots may also have up to 1 – 2 ‰ higher C isotope values than leaves[78]. However, these variations among the C_3 plants cannot explain the total variation of the $\delta^{13}C$ values observed among the burials from Rákóczifalva. Instead, the extent of the data spectrum indicates the availability of a C_4 plant component, which was most likely millet. Broomcorn millet (Panicum miliaceum L.) and foxtail millet (Setaria italica (L.), P. Beauv.) are the only major domesticated plants indigenous to Eurasia that rely on the C_4 photosynthetic pathway. Both species were cultivated in Northern China in the 6th millennium BC[79]. Millet has a shorter growing season and lower requirements for water and nutrients than C_3 cereals, is adapted to subtropical and temperate climate zones, is gluten-free and enriched in several essential amino acids[80]. Millets grown under modern controlled conditions have $\delta^{13}C$ values of typical of C_4 plants of between -13.6 and -12.7 ‰[79]. Applying a simple linear mixing model that assumes similar C contents of foodstuffs of C_3 and C_4 plant origin, suggests millet contributions at Rákóczifalva that varied from a few percent up to almost half of the dietary carbon, either taken up directly or with dairy products or meat of animals who fed on millet or its by-products. Archaeobotanical investigations of features of the Avar period in the Carpathian basin documented both, common C_3 cereals, such as barley, bread wheat, rye and oat, but also broomcorn millet providing direct evidence of the presences of the C_4 cereal during the respective time[81]. Moreover, previous analyses of skeletal remains of the Avar period from the Carpathian Basin[82,83], modern-day Austria[84] and Croatia[85] documented similar data ranges with elevated $\delta^{13}C$ values being attributed to millet consumption.

The $\delta^{15}N$ values varied from 9.82 to 13.53 ‰. An adult male individual (RKC026) of the middle/late Avar period stands out with a $\delta^{15}N$ value of below 10 ‰. Most data ranged between about 11 and 12.8 ‰, whereas exclusively male individuals formed a group that extended up to the maximal value of above 13.5 ‰. It is common sense that $\delta^{15}N$ values reflect trophic levels of plants and animals in the same habitats. In human diet reconstruction, this translates into a measure of the contribution of meat and dairy products[86,87]. Considering that nitrogen isotope fractionation from one trophic level to the next is around 3 to 5 ‰[88], the observed variation points to substantial variation in the shares of animal-derived foodstuffs of the sampled individuals. However, the isotope composition of the nitrogen that plants take up from the soil also varies spatially, depending on the content and composition of organic matter and human measures to increase soil fertility, such as manuring the agricultural land with animal dung[89,90]. Recent research shows that this does not only apply to the common C_3 staple crops, but manuring also increases $\delta^{15}N$ values of millet by up to 5-7 ‰[79,80].

In general, lower $\delta^{15}N$ values indicate less meat and dairy products in the human diet than higher $\delta^{15}N$ values. Nevertheless, for single individuals it cannot be excluded that a personal origin from a location with diverging baseline values and too short time of bone turnover to display the isotopic signals of food coming from the economic hinterlands of Rákóczifalva also shapes the N isotope record. Moreover, especially for the individuals with the highest $\delta^{15}N$ values, other foodstuff than meat and dairy products of domestic herbivores may also have played a role. Among them are animals of a higher trophic level, including suckling infants[72] or fish that tend to have higher $\delta^{15}N$ values than terrestrial herbivores[91,92].

As no data of contemporary animal collagen and botanical remains are currently available, our analysis highlights general trends, but cannot apply more advanced tools to quantify the possible dietary compositions, such as Bayesian mixing models[93,94]. In comparison with previously published datasets of the Avar period, the $\delta^{15}N$ values from Rákóczifalva are on average higher than those of the majority of sites including Sajópetri[83] and Tiszafüred[82] in the Carpathian basin, several locations in modern-day Austria[84] and Nustar in Croatia[85]. Only the burials at Hortobágy, which appeared to have outstandingly high $\delta^{15}N$ values[82], yielded on average higher $\delta^{15}N$ values. The analytical values from Rákóczifalva ranged between those from Hortobágy and all other sites[82]. Due to the lack of baseline data, it is currently impossible to differentiate and quantify the influence of inter-regional variation of the baseline values and different shares of animal-derived foodstuffs. However, in general, and also in comparison to nitrogen isotope data of burials from other time periods[77,95], the analytical values of human collagen of the Avar period in general, and from Rákóczifalva in particular, are comparatively high. They are consistent with substantial contributions of animal-derived foodstuffs to the human diet.

In summary, the C and N isotope data of the burials from Rákóczifalva point to diets that ranged from being largely based on C_3 plants at the base of the foodwebs to those to which millet, the only dietary C_4 crop, contributed almost half of the dietary carbon. The $\delta^{15}N$ values attest to a mixed diet, with varying, and for many individuals substantial, contributions of meat and dairy products of domesticated animals, whereas the meat of suckling infant animals or fish may also have played a role, especially for the individuals with the highest $\delta^{15}N$ values. The $\delta^{15}N$ values are high in comparison to other more ancient and contemporary communities. They point to a substantial contribution of animal-derived foodstuffs, even though weighting dietary compositions versus the influence of diverging local baselines is limited.

Internal differentiation

As discussed above, the C and N isotope compositions of the bone collagen of the members of the burial community of the cemetery at Rákóczifalva exhibited remarkable variation. In the following, we touch on the complex and interdepending association of their indications regarding dietary differences

according to age, sex, chronological placement, social status and biological relatedness as referred from the pedigrees.

Age

The evaluation of dietary habits of subadult individuals as opposed to those of the adults and weaning patterns were not a major focus of the sampling strategy. Summary statistics of the C and N isotope data of the representatives of age groups infans I, infans II and juvenile (both genetic sexes combined) and adult females and males are presented in Supplementary Table 8.

	**$\delta^{13}C$ (‰ V-PDB)**				**$\delta^{15}N$ (‰ AIR)**				
	**Min**	**Max**	**Avg.**	**Sd.**	**Min**	**Max**	**Avg.**	**Sd.**	**n**
**Inf. I**	-18.10	-16.83	-17.79	1.05	9.95	13.10	11.71	0.99	6
**Inf. II**	-17.86	-16.29	-17.25	0.60	11.02	12.47	11.91	0.53	6
**Juvenile**	**-18.71**	-15.70	-17.55	1.05	11.07	12.72	12.00	0.55	6
**Adult Female**	-18.58	**-14.33**	-17.28	0.94	10.94	12.55	11.88	0.42	36
**Adult Male**	-18.28	-14.84	-17.14	0.71	**9.82**	**13.53**	12.17	0.62	57
**Adult indet.**	-17.27				12.18				1

Supplementary Table 8 - Summary statistics of the $\delta^{13}C$ and $\delta^{15}N$ values of bone collagen of the burials from Rákóczifalva. Bold fonts highlight the minimal and maximal values across the whole dataset.

Overall, the isotope data of the subadult and the adult individuals overlapped widely, especially in the range of the densest cluster of $\delta^{13}C$ values of between about -18.5 and -16.5 ‰ and $\delta^{15}N$ values of between about 11.0 and 12.5 ‰ (Supplementary Fig. 24). An infant individual (RKF139) is noteworthy. The boy died at an age of 1-3 years and its comparatively high $\delta^{15}N$ value of 13.1 ‰, points to an influence of breastfeeding[96]. The burial did not contain any grave goods. Both parents were identified at the site, which suggests shaping of the isotopic signals by locally sourced foodstuffs. The other

representatives of the infans I age group were already older and consumed a diet that was generally similar to the diets of the adults. Supplementary Fig. 24 shows that four subadult individuals have $\delta^{15}N$ values at the lower section of the main data cluster, around 11 ‰. Low nitrogen isotope values have been previously reported for subadult individuals, who lived beyond their weaning age[97,98]. They likely indicate smaller shares of animal-derived foodstuffs to their diet, as there was no clear evidence for a systematically different N isotope fractionation due to growth[99].

Chronology

Regarding changes of dietary compositions over time, we considered individuals of the age groups infans II and older, which excluded the influence of possibly deviating diets during breastfeeding and shortly after weaning.

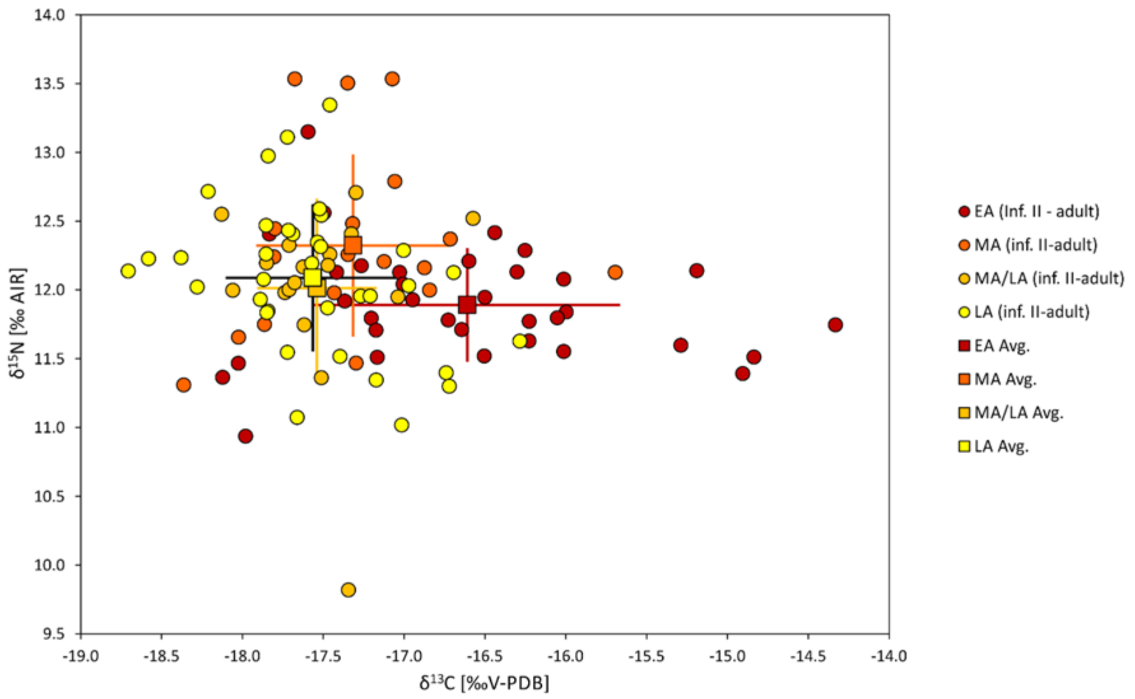

Supplementary Fig. 25 - Scatter plot of $\delta^{13}C$ and $\delta^{15}N$ values of bone collagen from the burial community of Rákóczifalva 8 and 8A (excl. age group infans I). The colors indicate the chronological association of the individuals. The error bars reflect 1 SD from the averages of the different phases of the Avar period: Early Avar n=34, Middle Avar n=18, Middle/Late Avar n=17, Late Avar n=35. Sample numbers for the phases of the Avar period are also given Supplementary Table 9.

The $\delta^{13}C$ values of the representatives of all phases of the Avar period overlap (Supplementary Fig. 25). However, the individuals of the early Avar period (EA) have a higher average value (Supplementary

Table 9) than those of the the middle (MA), middle/late (MA/LA) and late Avar (LA) phases of the Avar period. The data of the EA differed significantly from those of the later phases, whereas those of the MA, MA/LA and LA were statistically indistinguishable from each other (ANOVA - df: 3,100; F: 19.32; p: 5.832E-10; Tukey's pairwise test – Early vs. Middle p: 1.713E-04; Early vs. Middle/Late p: 3.47E-07; Early vs. Late p: 2.369E-09).

	**$\delta^{13}C$ (‰ V-PDB)**				**$\delta^{15}N$ (‰ AIR)**				
	**Min**	**Max**	**Avg.**	**Sd**	**Min**	**Max**	**Avg.**	**Sd**	**n**
**early Avar**	-18.12	**-14.33**	-16.61	0.95	10.94	13.15	11.89	0.41	34
**middle Avar**	-18.37	-15.70	-17.32	0.60	11.31	**13.53**	12.32	0.66	18
**middle/late Avar**	-18.13	-16.57	-17.54	0.37	**9.82**	12.71	12.01	0.65	17
**late Avar**	**-18.71**	-16.29	-17.57	0.54	11.02	13.35	12.09	0.54	35
**Middle-Late summary**	-18.71	-15.70	-17.49	0.52	9.82	13.53	12.13	0.60	70

Supplementary Table 9 - Summary statistics of the $\delta^{13}C$ and $\delta^{15}N$ values of bone collagen of the burials from Rákóczifalva. Bold fonts highlight the minimal and maximal values across the whole dataset.

The different distributions of the data are also reflected in the boxplots (Supplementary Fig. 26) and histograms (Supplementary Fig. 27), which show the higher frequency of $\delta^{13}C$ values of above -17 ‰ during the EA compared to the subsequent phases of the Avar period. The wider data distribution during the EA is also reflected in the larger standard deviations than observed for the MA, MA/LA, and LA (Supplementary Table 9).

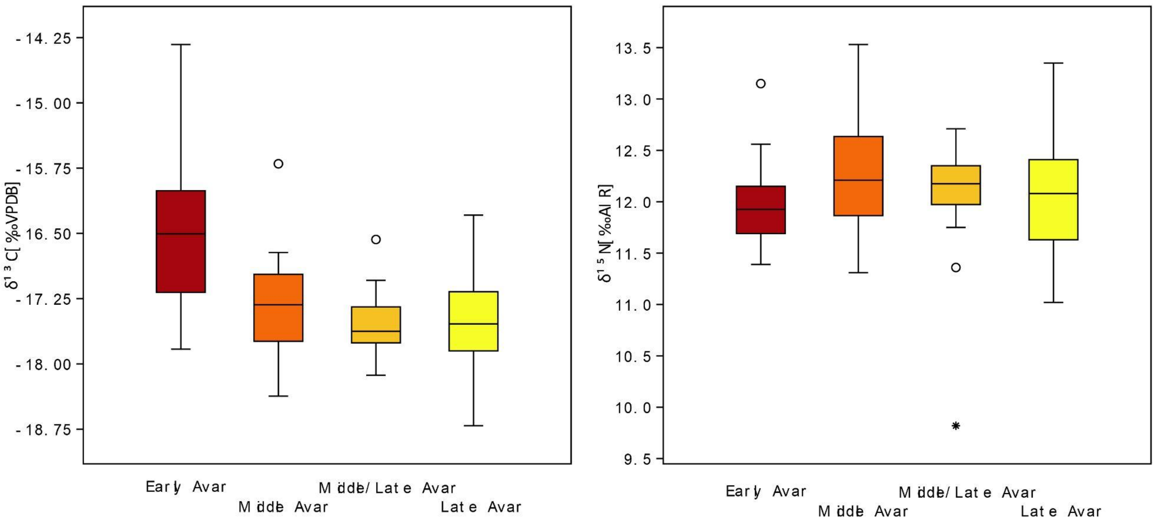

Supplementary Fig. 26 - Box plots of the $\delta^{13}C$ (left) and $\delta^{15}N$ values (right) of bone collagen of the burials from Rákóczifalva differentiated according to chronological phase of the Avar period. (Early Avar n=34, Middle Avar n=18, Middle/Late Avar n=17, Late Avar n=35. Sample numbers for the phases of the Avar period are also given Supplementary Table 9. The plot shows the medians (black line in the box), upper and lower quartiles (the contour of the box) and the whiskers as the minimum and maximum values. Outliers were marked with the respective function of Past software[100] (empty circles - values further than 1.5 times the box height from the box; asterisks - values further than 3 times), whiskers in these cases represent the next minimum/maximum values.

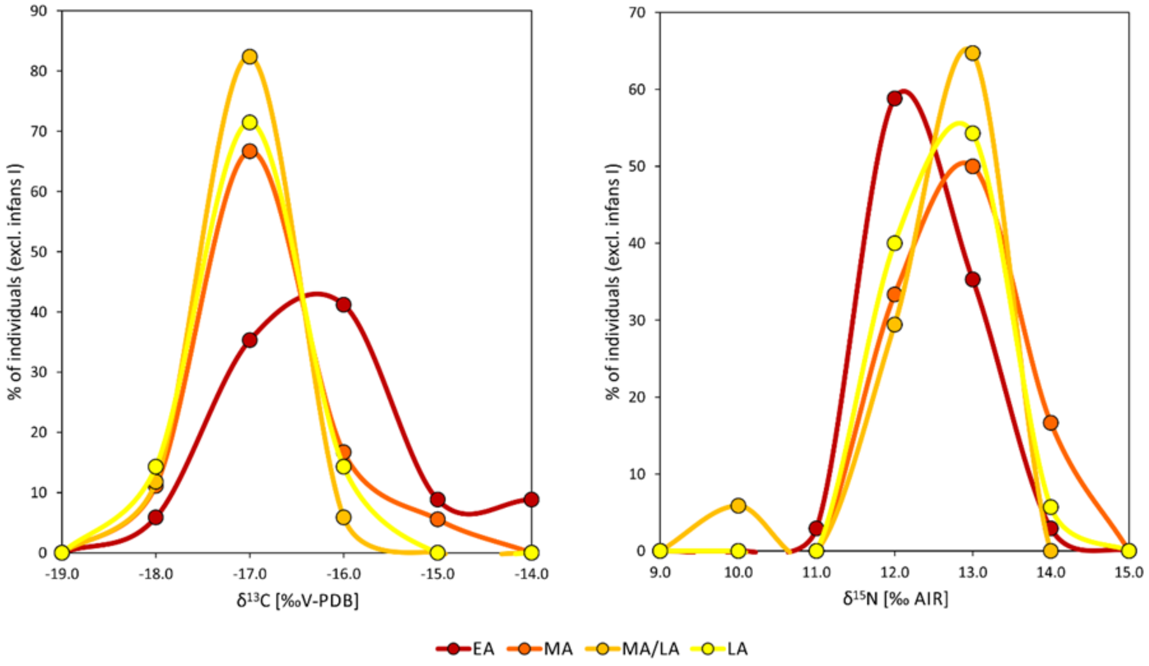

Supplementary Fig. 27 - Histograms of the relative frequencies (% of 104 individuals) of 1-‰-classes of $\delta^{13}C$ and $\delta^{15}N$ values of the representatives of the different chronological phases of the burials at Rákóczifalva (excl. age group infans I).

The difference between the diets of the people of the early and the middle to late phases of the Avar period can be assigned to variations in millet consumption. During the EA, the $\delta^{13}C$ values of about half of the individuals ranged between -16.5 and -14 ‰ which corresponds to millet contributing 25 – 50 % of the dietary carbon. During the MA and LA, almost all data correspond to the lower half of the range of the EA data and values above -16.5 ‰ are exceptional. The decreasing number of individuals consuming high-millet diets from the EA to the MA and LA, points to a decline of the importance of millet in the human diet.

The $\delta^{15}N$ values also overlapped widely among the samples of the different phases of the Avar period (Supplementary Fig. 25). However, the average $\delta^{15}N$ value of the representatives of the EA tended to be slightly lower than of the individuals of the MA to LA (Supplementary Table 9). The difference between the EA and MA individuals was significant, but between the EA and LA it was not (Mann-Whitney pairwise test: Early vs. Middle U: 165.5, p: 0.04868). The variations among the data distributions are also apparent in the box plot (Supplementary Fig. 26) and histogram (Supplementary Fig. 27). Overall, the differences in the nitrogen isotope compositions are more subtle than those of the $\delta^{13}C$ values. While the centers of the data clusters of the different phases largely overlap, the slightly higher averages of the MA to LA individuals are primarily caused by a group of individuals with higher $\delta^{15}N$ values, among which MA and LA individuals prevail. The data do not indicate a steady increase

of the $\delta^{15}N$ values over time. Instead, they point to similar shares of meat, dairy products or fish for most individuals during all three phases of the Avar periods, while representatives of the MA and LA prevail in a group of individuals who consumed relatively more animal-derived foodstuffs.

Overall, the C and N isotope data indicate a mixed diet that consisted of C_3 plants and millet as a C_4 plant at the base of the foodweb and animal derived foodstuffs. While there is considerable overlap among the data from the different phases of the Avar period, noteworthy changes occurred between the EA and successive phases. They include a decrease in the number of individuals with considerable millet consumption and an increase of a group of individuals who consumed more foodstuffs that go back to a higher trophic level. This finding is consistent with the population shift revealed by genetic data and changes in related archaeological phenomena.

Sex and social differentiation

Considering the adult individuals of all chronological phases, the isotope data of males and females overlapped widely (Supplementary Fig. 24). The average values of the $\delta^{13}C$ values of the females and males were very similar (Supplementary Table 8) and the subtle differences were not significant. A lot of outliers occurred both in the group of females (575/687, RKF069 and 744/874, RKF132), and males (547/650, RKF062; 598/726, RKF074; and 596/721, RKF227), their diet comprised significantly more millet. Among these burials, 744/874 (RKF132) is one of the earliest graves of the cemetery (564-596 cal AD), an unrelated female in an end-niche grave. 596/721 (RKF227) and 598/726 (RKF074) (606-646 cal AD), which lie slightly separated from other graves, form the small EA pedigree 14. These two male graves were located on two sides of the - unrelated - female's grave 575/687 (604-641 cal AD), which had similarly high $\delta^{13}C$ values, pointing to substantial millet consumption. In this case, it was possible to link biological and likely social grouping to a certain diet. Because of the lack of normal distributions, the medians of the $\delta^{13}C$ values of females and males were compared, but there was no significant difference (Mann-Whitney - U: 833, p: 0.232). The boxplots (Supplementary Fig. 28) and the histograms (Supplementary Fig. 29) illustrate the almost identical patterns of the distribution of the $\delta^{13}C$ values of adult males and females.

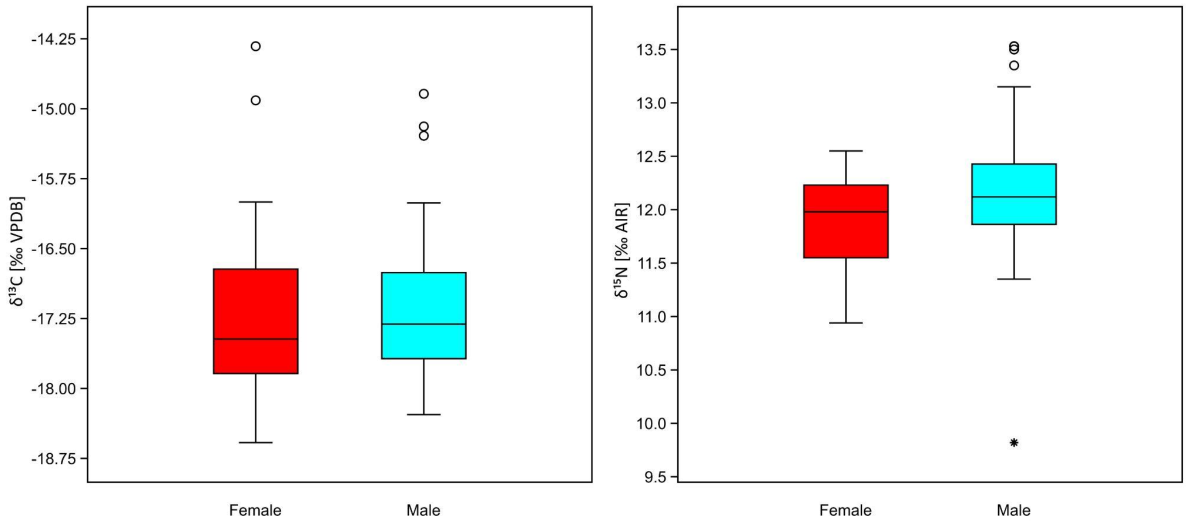

Supplementary Fig. 28 - Box plots of the $\delta^{13}C$ (left) and $\delta^{15}N$ values (right) of bone collagen of the burials from Rákóczifalva differentiated according sex of the adult individuals. (Female n=36, male n=57. The plot shows the medians (black line in the box), upper and lower quartiles (the contour of the box) and the whiskers as the minimum and maximum values. Outliers were marked with the respective function of Past software[100] (empty circles - values further than 1.5 times the box height from the box; asterisks - values further than 3 times), whiskers in these cases represent the next minimum/maximum values.

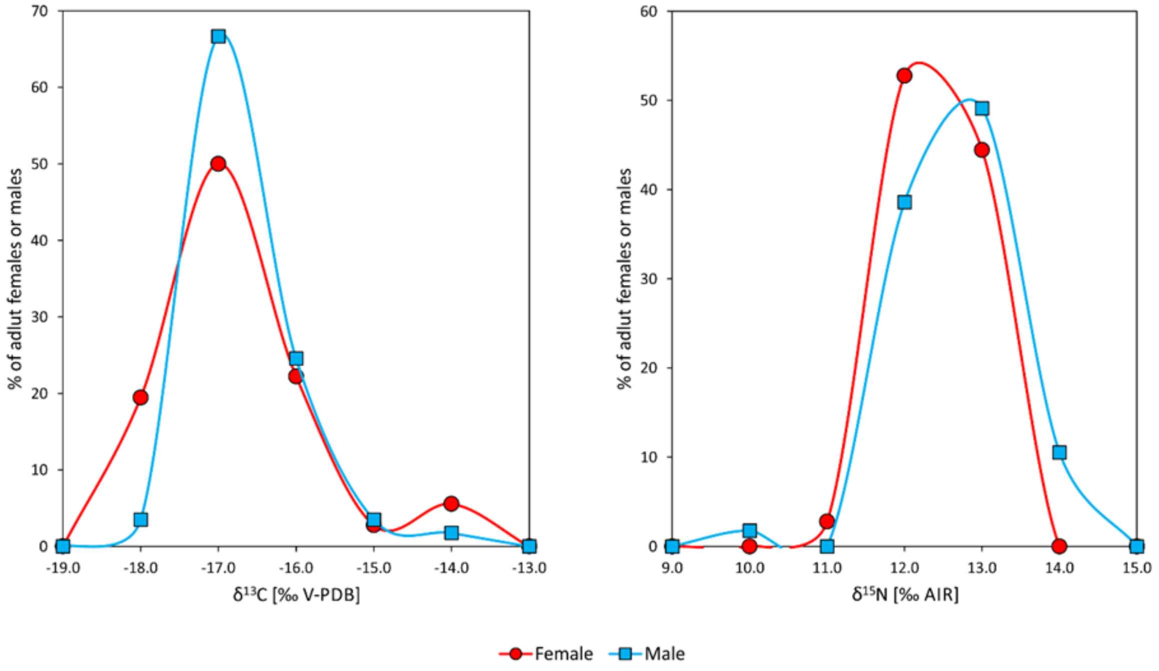

Supplementary Fig. 29 - Histograms of the relative frequencies (% of 36 female and 57 male

individuals) of 1-‰-classes of $\delta^{13}C$ and $\delta^{15}N$ values of adult females and males among the burials at Rákóczifalva.

The trend of largely indistinct $\delta^{13}C$ values of males and females persisted throughout the Avar period. In the EA, the ranges and averages of the values for both sexes were almost identical (Supplementary Table 10; Supplementary Fig. 28 and 25). Respectively, the remarkable variation from average diets that were largely dominated by C_3 plants to those that comprised considerable amounts of millet, were not sex specific. The drastic reduction of evidence for $\delta^{13}C$ values of > -16.5 ‰ in the successive phases of the Avar period affected both sexes, indicating that discontinuation of high-millet diets was primarily an effect of variation over time than it was specific to males or females.

**$\Delta^{13}C$**	**Female**					**Male**				
	**Min**	**Max**	**Avg**	**Sd**	**n**	**Min**	**Max**	**Avg**	**Sd**	**n**
**Early Avar**	-18.12	-14.33	-16.59	1.16	13	-17.83	-14.84	-16.59	0.82	20
**Middle Avar**	-18.37	-17.30	-17.82	0.44	4	-18.03	-16.72	-17.25	0.39	11
**Middle/Late Avar**	-18.13	-17.33	-17.68	0.27	9	-17.71	-16.57	-17.31	0.40	7
**Late Avar**	-18.58	-16.72	-17.56	0.67	9	-18.28	-16.69	-17.59	0.36	18
**Middle-Late summary**	-18.58	-14.33	-17.66	0.51	35	-18.28	-14.84	-17.43	0.40	56

Supplementary Table 10 - Summary statistics of the $\delta^{13}C$ values of bone collagen of adult females and males of the different phases of the Avar period at Rákóczifalva.

$\Delta^{15}N$	Female					Male				
	**Min**	**Max**	**Avg**	**Sd**	**n**	**Min**	**Max**	**Avg**	**Sd**	**n**
**Early Avar**	10.94	12.29	11.71	0.36	13	11.51	13.15	12.00	0.42	20
**Middle Avar**	11.31	12.45	11.87	0.56	4	11.66	13.53	12.57	0.68	11
**Middle/Late Avar**	11.75	12.55	12.14	0.24	9	9.82	12.71	11.82	0.98	7
**Late Avar**	11.30	12.54	11.94	0.44	9	11.35	13.35	12.24	0.52	18
**Middle-Late summary**	10.94	12.55	12.01	0.44	35	9.82	13.53	12.26	0.70	56

Supplementary Table 11 - Summary statistics of the $\delta^{53}N$ values of bone collagen of adult females and males of the different phases of the Avar period at Rákóczifalva.

In contrast, sex had a larger influence on the $\delta^{15}N$ values (Supplementary Fig. 24, Supplementary Table 11), which were significantly higher in males (12.2 ± 0.6 ‰) than in females (11.9 ± 0.4 ‰) (Mann-Whitney - U: 712.5, p: 0.029375). The difference was primarily due to a group of seven burials with $\delta^{15}N$ values of ca. 13 ‰ and above, which exclusively consisted of males. Statistically, the low value of individual 147/232 was an outlier pointing to a very low intake of animal-derived protein. The high values of 410/510 (RKF012), 446/546 (RKF042), and 171/266 (RKC033) are outliers in the other direction pointing to the largest shares of animal-derived foodstuffs among all sampled individuals. Also, within the main data range between about 11 and 13 ‰, there was a slight trend of males presenting more often values towards the upper end and females more often having values towards the lower end of this spectrum (Supplementary Fig. 24). This subtle difference is also expressed in the boxplot (Supplementary Fig. 28) and in the histogram (Supplementary Fig. 29), which illustrates a peak of the data of females at 11-12 ‰ and of males at 12-13 ‰. Higher average $\delta^{15}N$ values in males than in females, persisted during all phases of the Avar period, except for the individuals who could not be clearly assigned to the MA or to the LA (Supplementary Table 11 and Supplementary Fig. 30).

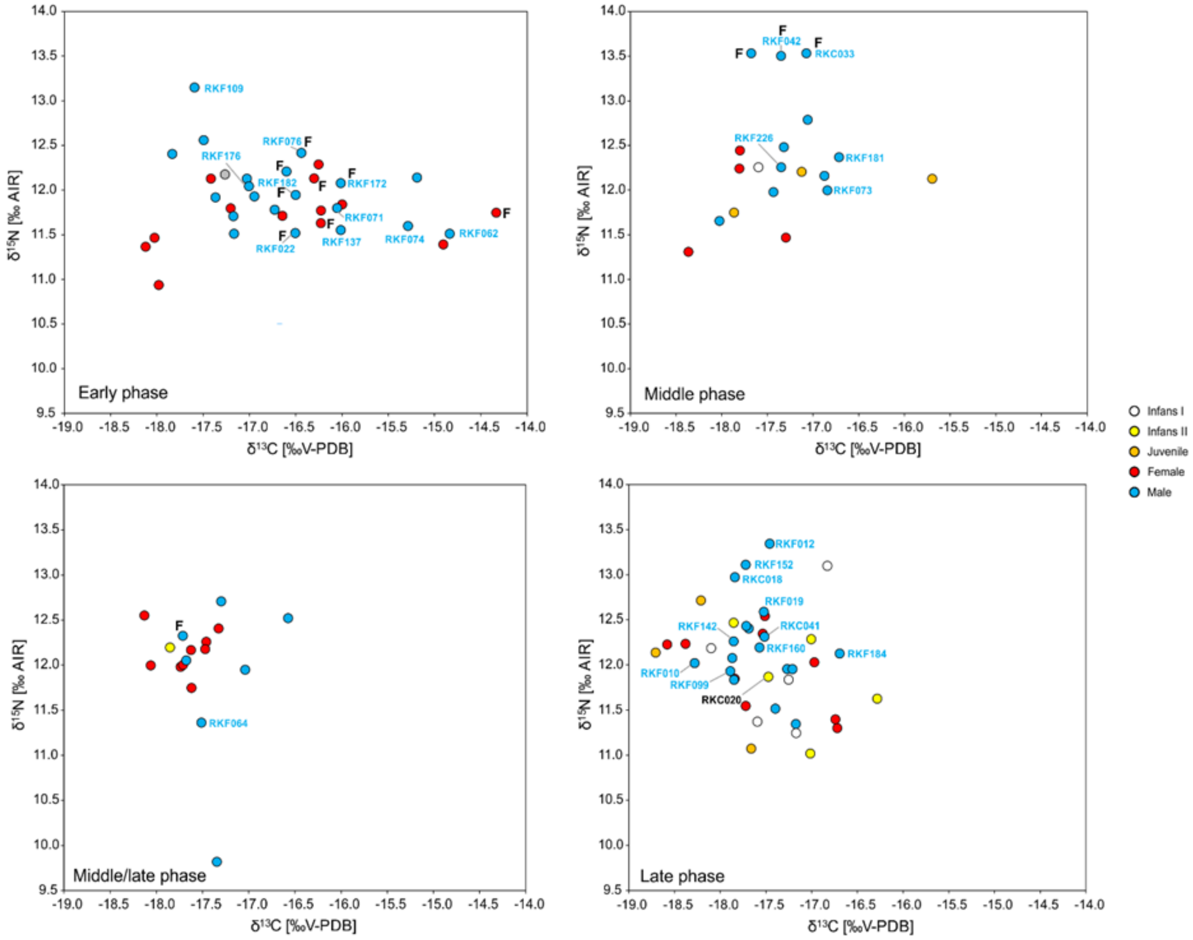

Supplementary Fig. 30 - Scatter plot of $\delta^{13}C$ and $\delta^{15}N$ values of bone collagen from the burial community of Rákóczifalva 8 and 8A differentiated according to chronological affiliation. The labels mark data of individuals whose burials comprised pieces of belt sets and horse/riding equipment. "F" marks individuals of the founder generation of the pedigrees.

The group of males with elevated $\delta^{15}N$ values comprised one individual of the EA and three individuals each of the MA and LA. With one exception (RKC047), all these individuals were buried with horse harnesses/riding equipment and/or belt sets that indicate an elevated social status. Moreover, three of the respective individuals represent the first generation (founders) of the large pedigrees 3 and 4 (RKC033, RKC047, RKF042), who in two cases had offspring with multiple females (RKC033, RKF042). Two further members of the group (RKF012, RKF152) were sons of RKF042. Considering that nitrogen isotope ratios indicate trophic positions, the analytical results suggest that larger shares of meat, dairy products and possibly fish in the diet added to a series of indicators of an elevated social status in the community. Similar parallels of grave furnishing that hinted on an affiliation with social elites and indication of access to supposedly higher quality foodstuffs, such as meat and/or dairy products in life, has equivalents from the Early Bronze Age to the Middle Ages[101].

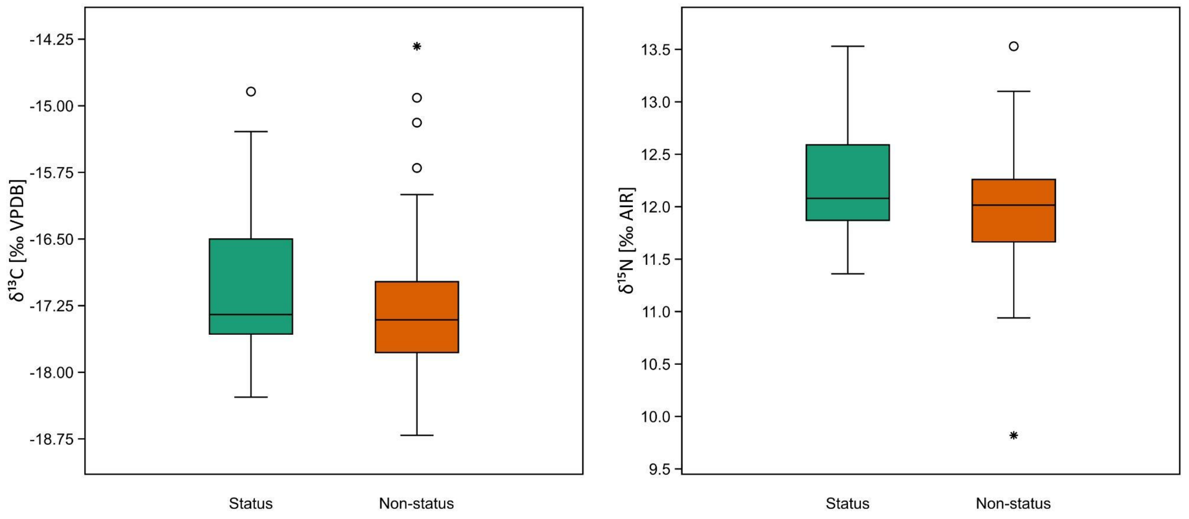

Supplementary Fig. 31 - Box plots of $\delta^{13}C$ (left) and $\delta^{15}N$ (right) values of individuals buried with and without status objects. (Status n=27, non-status n=80. The plot shows the medians (black line in the box), upper and lower quartiles (the contour of the box) and the whiskers as the minimum and maximum values. Outliers were marked with the respective function of Past software[100] (empty circles - values further than 1.5 times the box height from the box; asterisks - values further than 3 times), whiskers in these cases represent the next minimum/maximum values.).

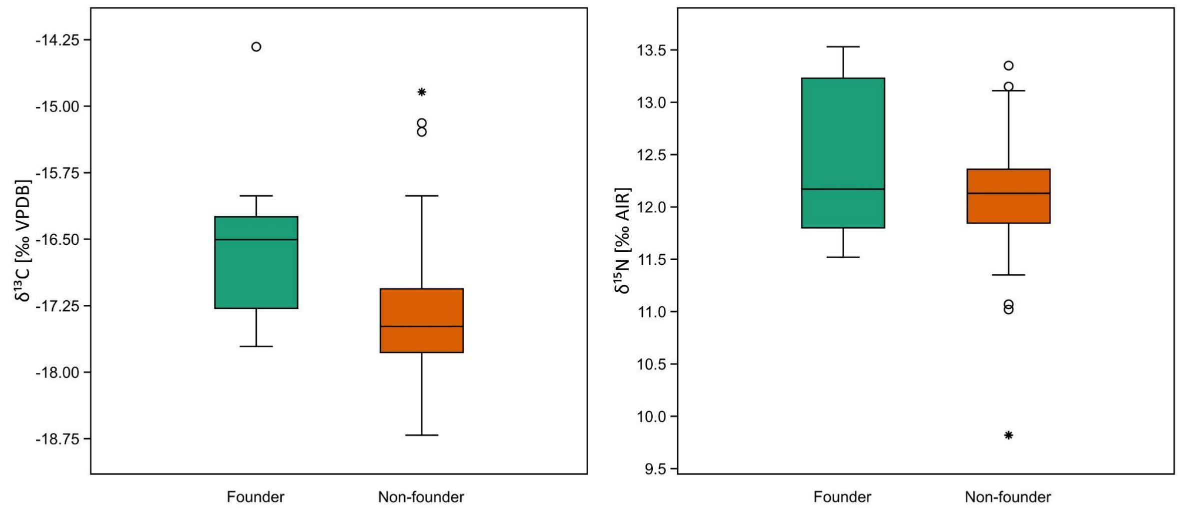

Supplementary Fig. 32 - Box plots of $\delta^{13}C$ (left) and $\delta^{15}N$ (right) values of pedigree founder and non-founder individuals. (Founder n=12, non-founder n=96. The plot shows the medians (black line in the box), upper and lower quartiles (the contour of the box) and the whiskers as the minimum and maximum values. Outliers were marked with the respective function of Past software[100] (empty circles - values

further than 1.5 times the box height from the box; asterisks - values further than 3 times), whiskers in these cases represent the next minimum/maximum values.).

At Rákóczifalva, however, the association of being buried with horse/riding equipment, (parts of) belt sets or belonging to the first genetically detected generation of a pedigree and elevated $\delta^{15}N$ values was not exclusive (Supplementary Fig. 30, 27 and 28), which was reinforced by comparing the medians of the two-two groups with Mann-Whitney test (status vs. non-status - U: 858.5, p: 0.1129; founder vs. non-founder - U: 491.5, p: 0.38349). Instead, there was a spectrum regarding the quality and quantity of these items so that not each single evidence of these categories of equipment may be considered indication of a distinguished social position in the community. Respectively, such burial items were not restricted to the individuals with outstandingly high $\delta^{15}N$ values, but also documented for males whose N isotope signals indicate moderate contribution of animal-derived foodstuffs to the average diet in life. Regarding the members of the first generation of the pedigrees, observations of variation were similar and also included a chronological component. The founders of the smaller pedigrees of the EA had moderate $\delta^{15}N$ values, indicating that representing the first generation at the site, was not exclusively related to a diet rich in animal-derived components. Moreover, apparently higher $\delta^{13}C$ values among members of founding generations in comparison to non-founders, are also primarily due to the larger numbers of pedigrees and founders dating to the EA than to the MA and LA, and millet being generally more common in EA diets (Mann-Whitney - U: 236.5, p: 9.1791E-4). The gradual and complex indicators of social differentiation within the community and their relation to dietary habits will be explored in more detail in a separate publication.

Detailed ^{14}C results and bayesian modeling

57 samples from the whole site-complex were selected for ^{14}C dating. All samples have good preservation with collagen yields above the critical value of 0.5% where ^{14}C dating would not be carried out. CN-ratios are in the range of 2.9 - 3.3 showing well preserved collagen. Supplementary Table 6 summarizes the ^{14}C dating results. 27 samples are part of the pedigrees discussed here (Supplementary Fig. 33).

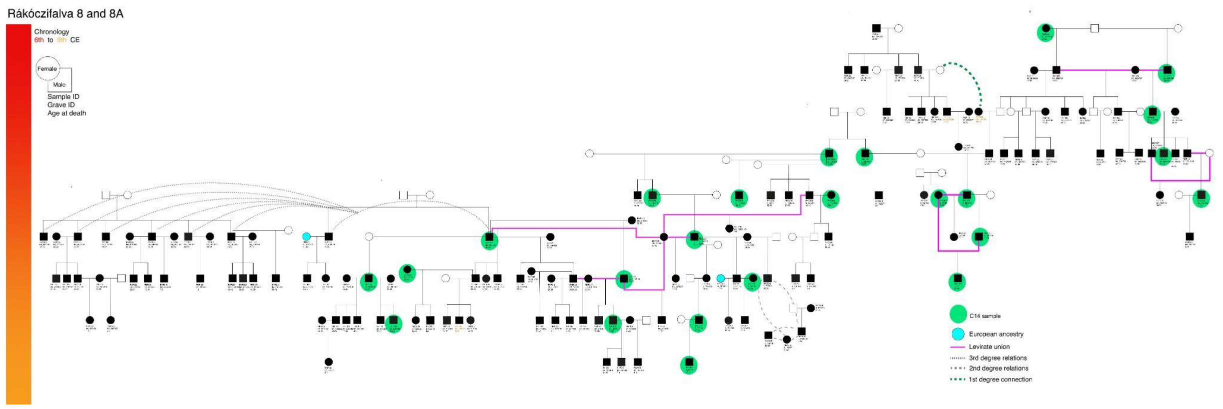

Supplementary Fig. 33 - ^{14}C samples from the extended pedigree 1-5 of Rákóczifalva.

Supplementary Fig. 34, shows the calibrated ^{14}C dates of those 27 samples graphically. Due to the shape of the calibration curve exhibiting two plateau sections around 750 CE and 850 CE (Supplementary Fig. 35), some calibrated date ranges (the 2-sigma probability distributions of the calibrated dates) are relatively wide, spanning up to 200 years. Exemplary, ^{14}C dating of the individuals of pedigree 2 (Supplementary Fig. 33) can distinguish the first generations (RKF056, RKF182 and RKF 148) from the younger ones.

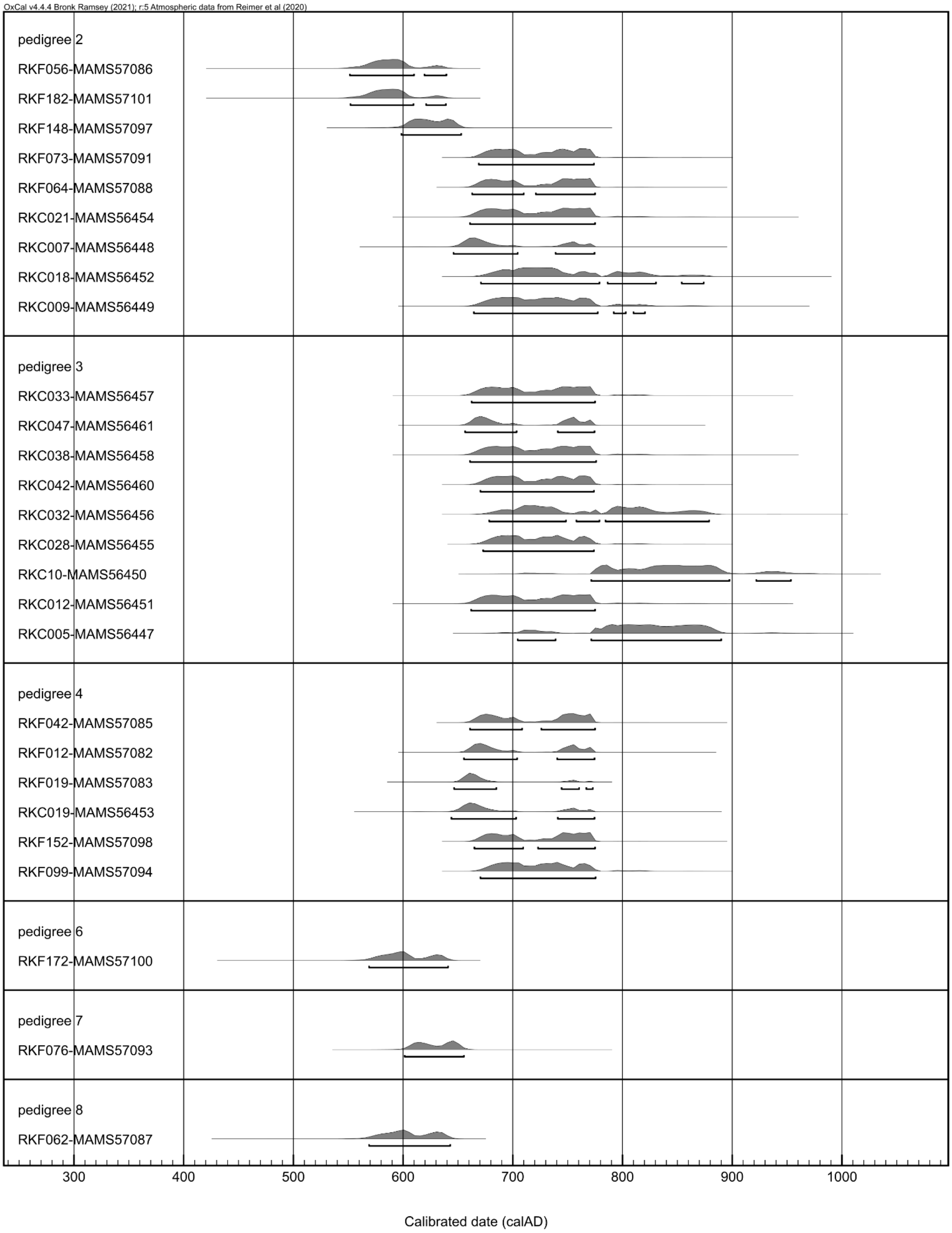

Supplementary Fig. 34 - Calibrated ^{14}C dates of 27 samples included in the pedigrees. The area under the curve indicates the probability of dating the individual to the corresponding time-period.

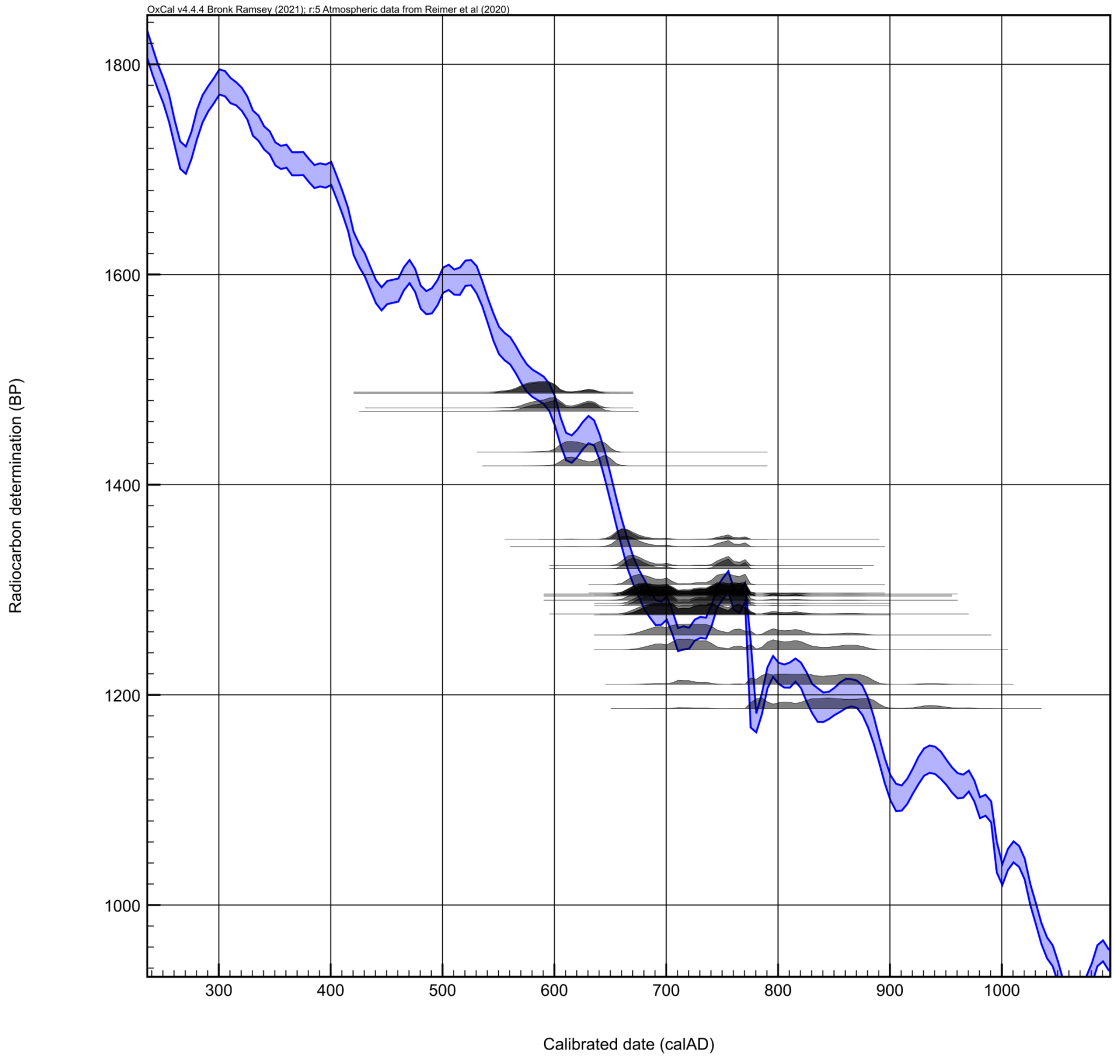

Supplementary Fig. 35 - ^{14}C calibration curve for the investigated time-period with studied samples probability distribution. Two plateaus around 750 CE and 850 CE can be observed.

However, all individuals in subsequent generations (RKF073, RKF064, RKC021, RKC007, RKC018 and RKC009) fall into the same calibrated ranges and can't be distinguished from each other. Reducing the uncertainty, respectively narrowing the calibrated range, is only possible by providing supporting information to the ^{14}C ages that carry some sort of chronological information. The pedigrees define a sequence of generations that have lived partially contemporary but also subsequent to each other and provide relative chronological information that can be modeled by a Bayesian approach. Massy et al. have shown the applicability of such an approach[102]. Pedigrees 2, 3 and 4 contain individuals that have been dated and lived in subsequent generations. The software OxCal [103] was used for Bayesian modeling incorporating the following information: (1) The ^{14}C dates of the individuals, (2) sequencing the ^{14}C dates of the individuals according to their generations using the command "sequence" , (3) constraining the length of a generation by adding an interval between the generations of 30 +- 15 years

and (4) correction the ^{14}C ages for the Human-Bone-Collagen-Offset (HBCO) in order to provide ^{14}C ages for the year of death [104]. Sequencing the generations (2) is possible since all individuals died of similar age - adultus and older. Hence, we can assume that children died after their parents. This fact also provides the possibility to constrain the generations to a minimum length (3) that is considering the minimum and maximum reproductive age of the parents. Since this information is not known for every individual a large uncertainty of +- 15 years so that this parameter can be between 15 to 45 years of age. This uncertainty is carried into the modeling procedure resulting in wider dating ranges. However, the certainty that the actual ages are within the modeled data is higher. ^{14}C ages of individuals of the same generation are grouped by the OxCal command "phase" so that there is no specific order of those dates. For some generations no direct parent-child connection is available so that the next possible member of one generation is used for modeling. This is not ideal since children of siblings, even though in the same generation, do not necessarily follow the assumptions, points 2 and 3, on which our model is based.

Considering all those prescribed information the following modeled ^{14}C dates are obtained (oxcal code is provided as Oxcal-files).

Pedigree 2: Supplementary Fig. 36 and 37 show the modeled ^{14}C dates compared to the unmodeled. First the right side of the pedigree is modeled containing the individuals (in that order) RKF056, RKF182, RKF148, RKF073, RKF064. Second the model is extended with the individuals of the left strain of the pedigree containing individuals (in that order) RKF056, RKF182, RKF148, RKF073, RKC021 and RKC007 (grouped in one phase), RKC018, RKC009. The uncertainty of some of the modeled data is reduced by about 50% (individual RKF073 of generation 4). While other dates (e.g. generation 5, 6 and 7) still exhibit a wide range, generally all date ranges are reduced. Concluding, the dated individuals of pedigree 2 span a time frame (2-sigma dates) beginning with 563-629 calAD (individual RKF056) and ending at 710-871 calAD (individual RKC009).

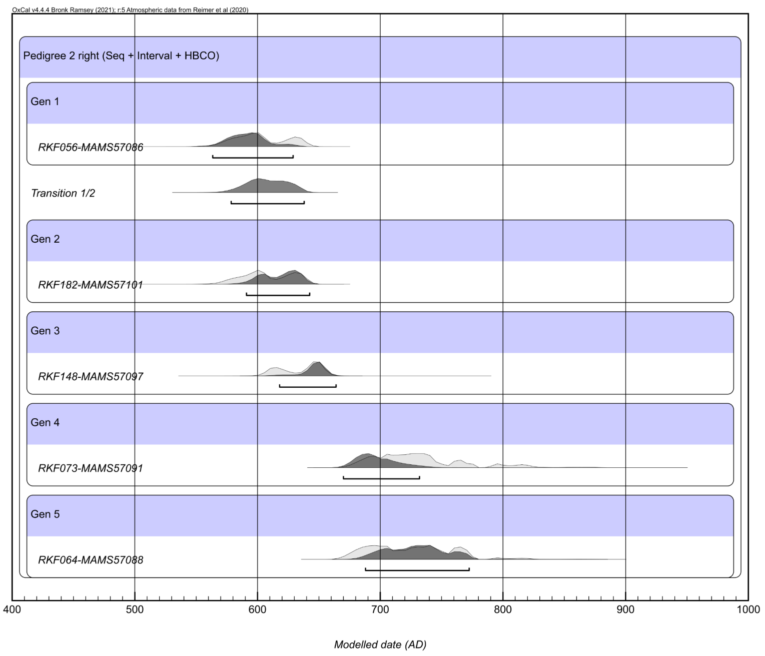

Supplementary Fig. 36 - Modeled ^{14}C dates of the studied individuals for each generation (Gen) on the right side of pedigree 2 compared to the unmodeled ^{14}C dates.

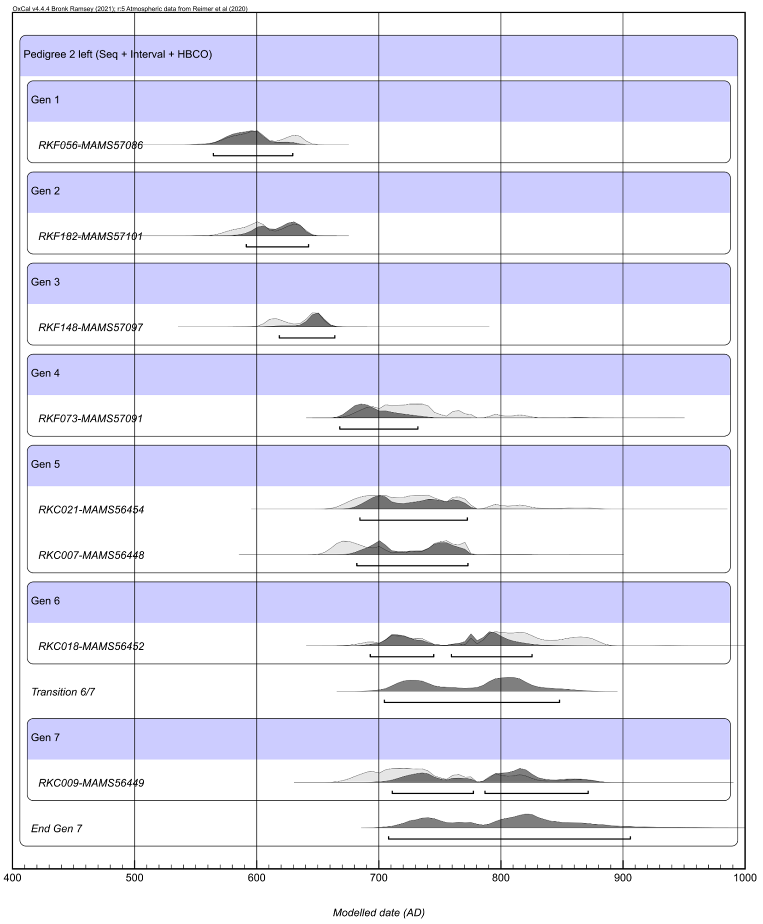

Supplementary Fig. 37 - Modeled ^{14}C dates of the studied individuals on the left side of pedigree 2 compared to the unmodeled ^{14}C dates.

Pedigree 3: Similarly to the individuals of pedigree 2 Supplementary Fig. 38 and 39 show the modeling results for pedigree 3 comprised of the individuals (in that order) RKC033 and RKC047 (one group), RKC038, RKC028, RKC010, RKC012, RKC005. This case is complicated by the fact that no date for individual RKC024 is available which would connect the other individuals more directly with each other. Alternatively, individual RKC010 from the same generation was used. However, RKC024 is a lineage daughter while RKC010 is her half-brother's exogamous partner. Hence, those individuals may have died at different times. The raw 14C dates suggest such since it is one of the youngest dates in this pedigree. However, not considering the date of RKC010 and just leaving the interval between the generations, does affect the model outcome slightly. In all cases the precision of the modeled ^{14}C dates is improved compared to the unmodeled values. Here, we will use the model results without using RKC010, which constrains the time span of the dated individuals of pedigree 3 (2-sigma dates) starting with 662 - 753 calAD (individual RKC047) and ending at 771 - 881 calAD (individual RKC005).

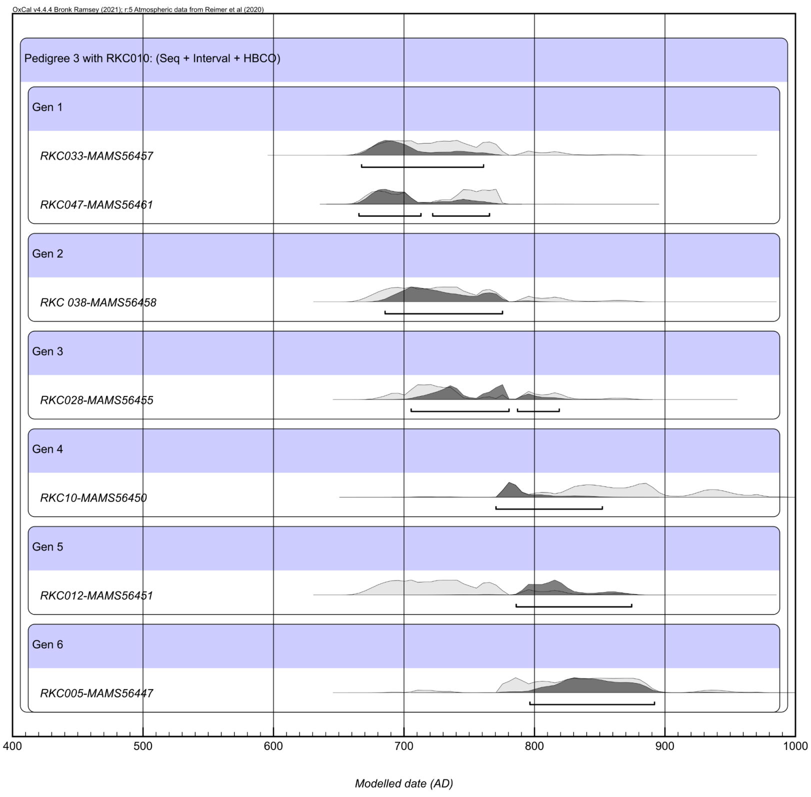

Supplementary Fig. 38 - Modeled ^{14}C dates of the studied individuals on the right side of pedigree 3 compared to the unmodeled ^{14}C dates.

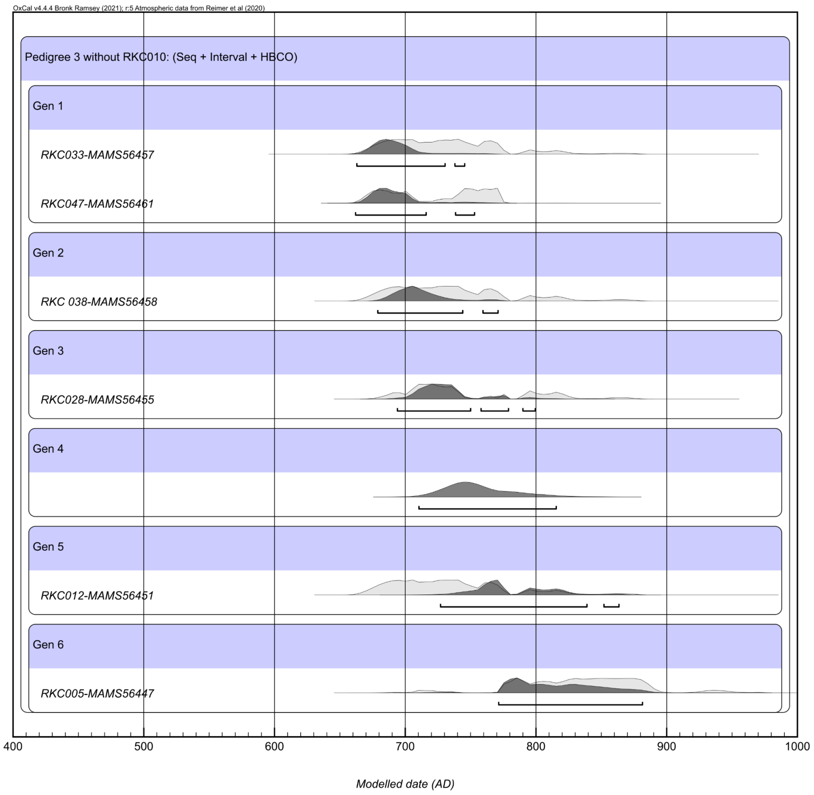

Supplementary Fig. 39 - Modeled ^{14}C dates of the studied individuals on the left side of the pedigree 3 compared to the unmodeled ^{14}C dates.

Pedigree 4: Pedigree 4 can be modeled using the left side of the tree (individuals RKF042, RKF012, RKF019) and the right side of the tree (individuals RKF042, RKF152, RKF099). The results are shown in Supplementary Fig. 40 and 41. The modeled dates of individual RKF042 which forms generation 1 in both models is constrained to a very similar age range independent of the side of the tree (right side of the tree: 665 - 755 calAD, left side of the tree: 665 - 766 calAD). Dependent on the modeled side of the tree, their dated individuals constrain pedigree 4 to a start date of (right side of the tree) 665 - 755 calAD (Individual RKF042) ending at 671 - 778 calAD (individual RKF019). Considering the left side of the tree, pedigree 4 starts at 665 - 766 calAD (individual RKF042) ending at 693 - 830 calAD (individual RKF099).

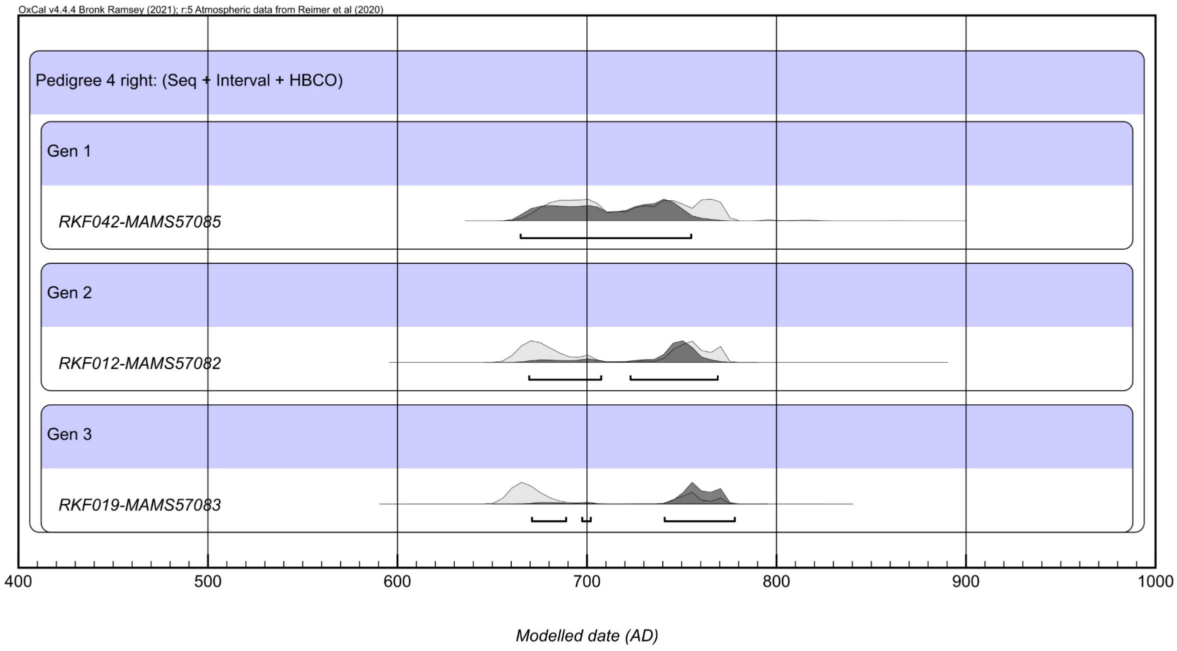

Supplementary Fig. 40 - Modeled ^{14}C dates of the studied individuals on the right side of the pedigree 4 compared to the unmodeled ^{14}C dates.

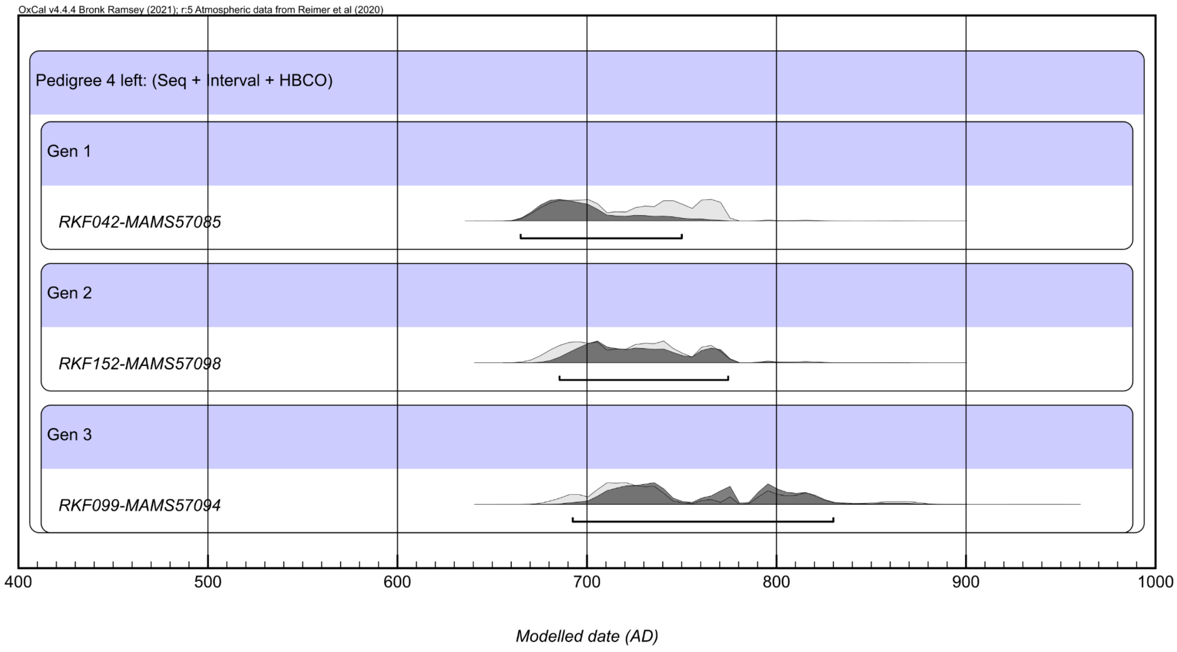

Supplementary Fig. 41 - Modeled ^{14}C dates of the studied individuals on the left side of the pedigree 4 compared to the unmodeled ^{14}C dates.

Pedigrees 3 and 4 are not entirely separate and independent from each other. Individual RKF140 was partner to an individual in pedigree 3 (RKC028) and pedigree 4 (RKF042). That provides the

opportunity to extend pedigree 4 into the older line of pedigree 3. Even though the order of deaths of individuals RKF042 and RKC038 is not well defined by the pedigree or archaeological information, individual RKC033 (pedigree 3) has certainly died before RKF042 (pedigree 4). Hence, adding RKC033 into pedigree 4 may better constrain the beginning of pedigree 4. Supplementary Fig. 42 shows the result of this kind of model setup for the right side of pedigree 4 (normal pedigree 4, right side + RKC033). The right side of pedigree 4 (starting with RKC042) can now be further constrained to have started at 683-760 cal AD (RKF042) ending at 742-776 calAD (RKF019). Besides shifting the beginning of the pedigree to slightly younger ages - as advised by pedigree 3 - the ambiguity of the date of RKF019 can be fully resolved and the dating range reduced substantially to only span 34 years.

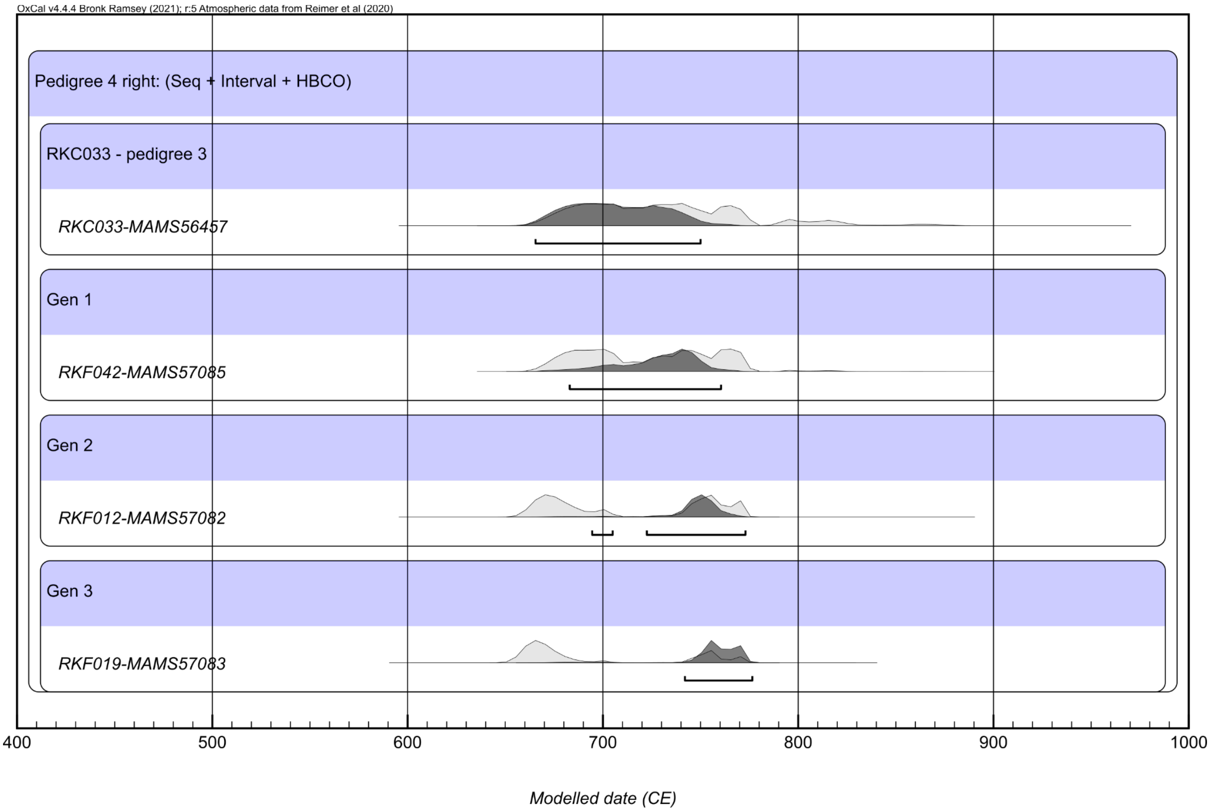

Supplementary Fig. 42 - Modeled ^{14}C dates of the studied individuals on the right side of the pedigree 4 compared to the unmodeled ^{14}C dates, including the RKC033 individual.

Pedigrees 6, 7 and 8: Those pedigrees only contain one dated individual so that bayesian modeling cannot be applied. However, even without modeling the results show that the dated individuals agree well with the oldest ones of pedigree 2 (see Supplementary Fig. 34)

Overall the pedigrees that contain dated individuals can be chronologically placed using ^{14}C as shown in Supplementary Fig. 42.

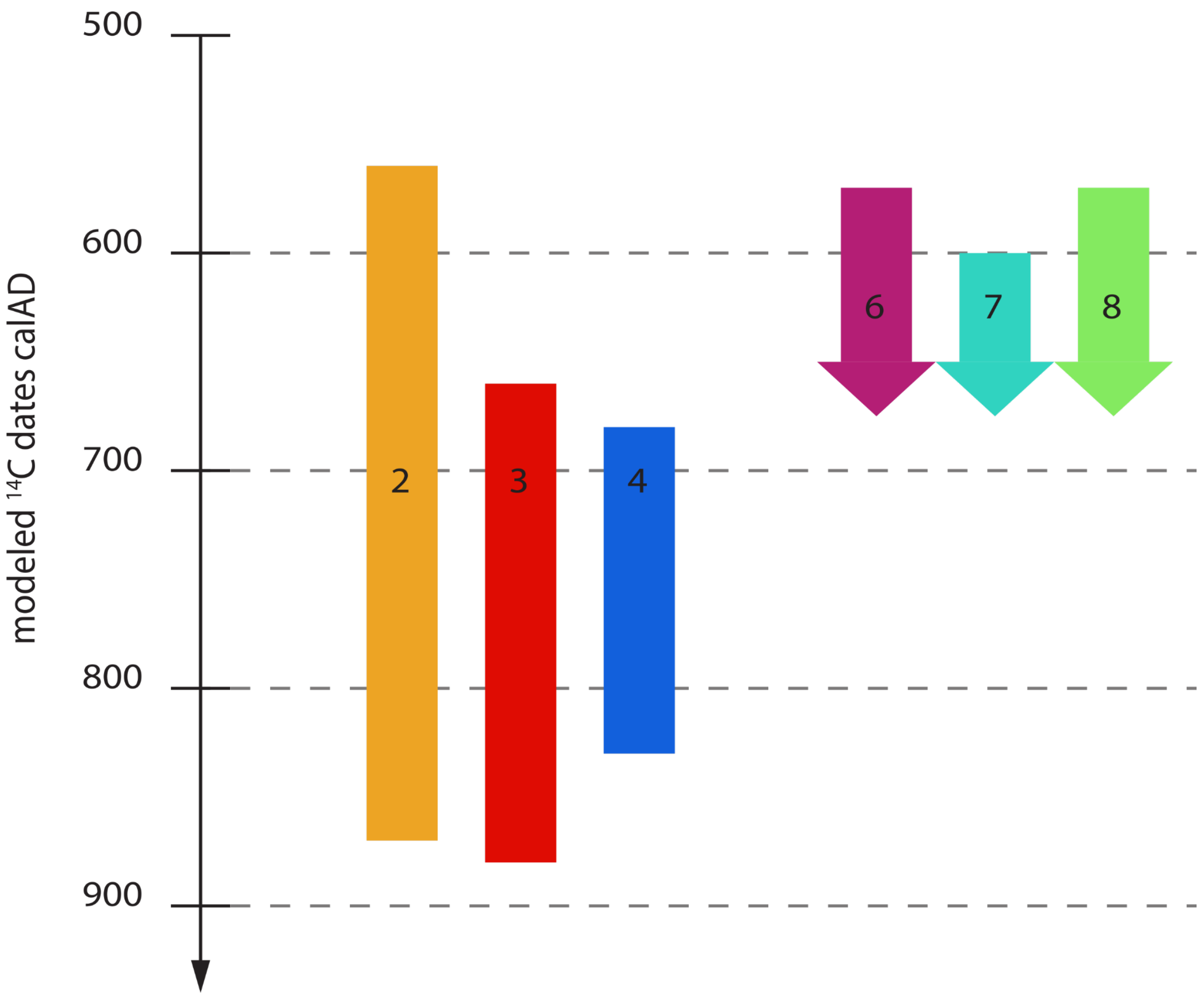

Supplementary Fig. 43 - Chronology of the pedigrees that contain ^{14}C dated individuals.

IBD network statistics

Considering the IBD network of the adults, the strength (*w*) distributions of males and females are significantly different (Methods) showing the same trend as the degree centrality (*k*) distribution reported in Fig. 4b (Supplementary Fig. 44).

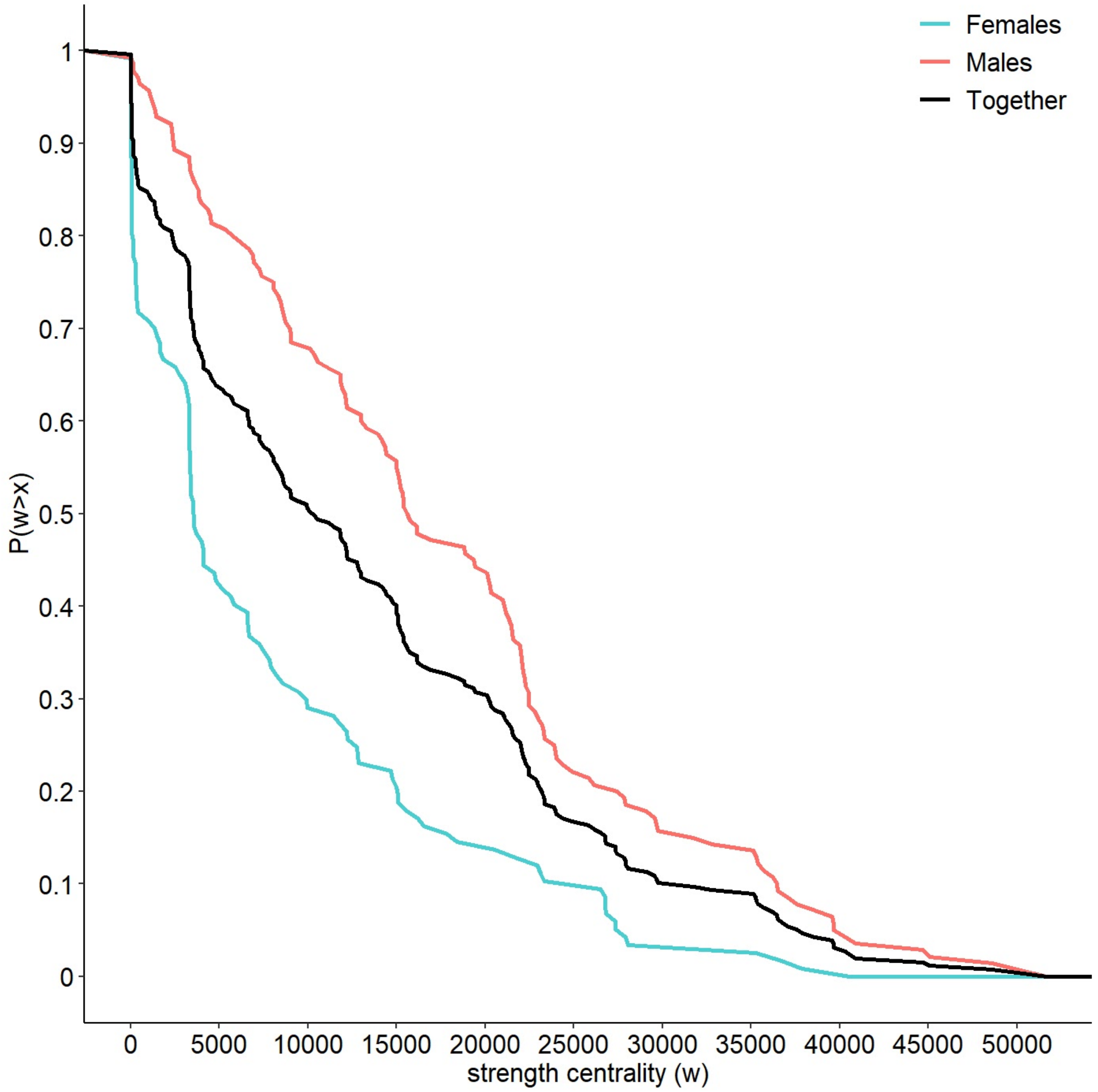

Supplementary Fig. 44 - The cumulative density function of the strength (w) distribution.

In order to analyze the average extent of relatedness that individuals have, we calculated the mean strength of the within- and between-module links. We considered the total sum of IBDs > 12cM as the weights of the links revealed that the mean between-module strength (mean w_B) did not differ between sexes. Moreover, these links have lower mean strength than the within-module (w_W) links. There are more females with large mean w_W but they do not have large k values as the size of the nodes indicates (Supplementary Fig. 45). They are the females who have a few first-degree relatives in the cemetery, but their lineages do not continue for a long time.

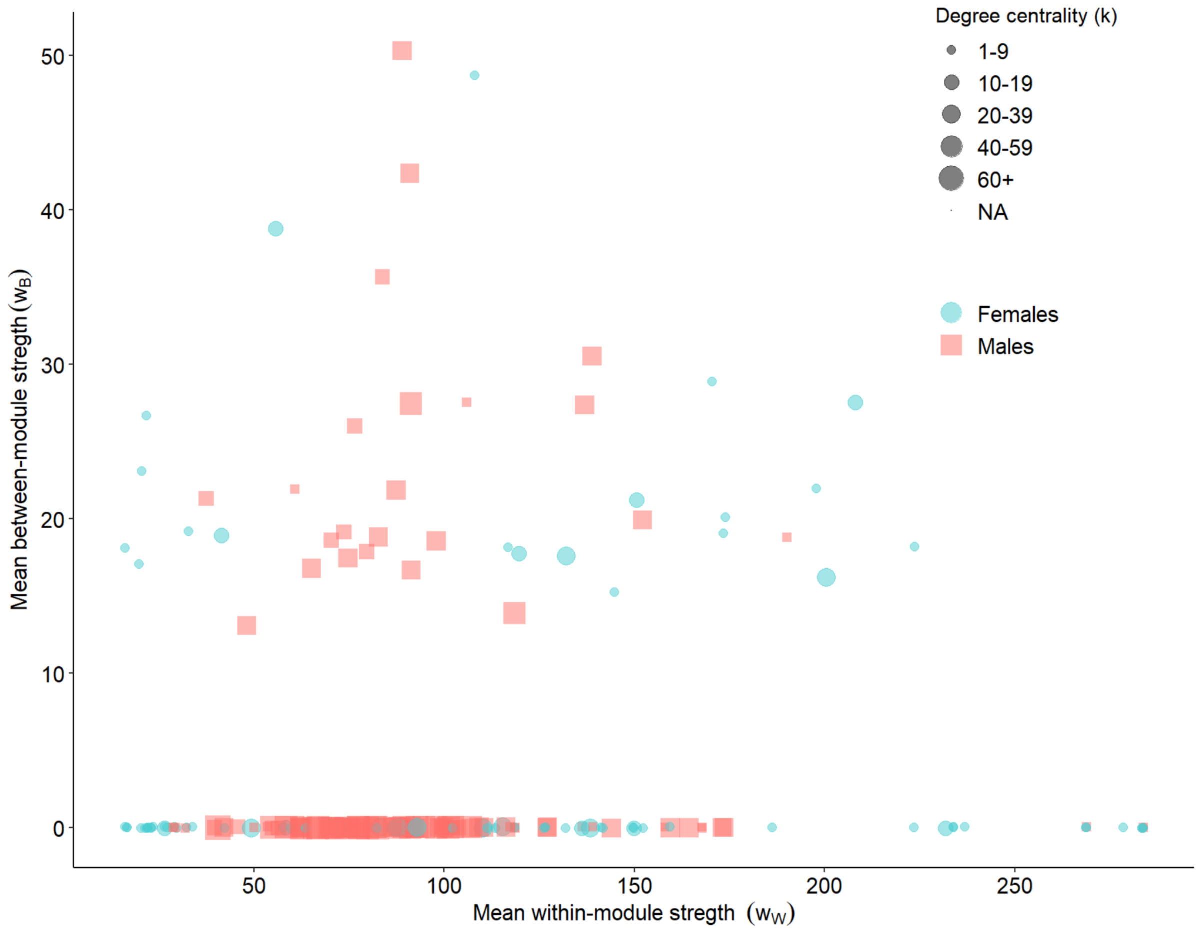

Supplementary Fig. 45 - Mean within-module strength (w_W) and mean between-module strength (w_B). The size of the points indicates degree centrality (k).

The clustering coefficient (C) measures how connected an individual's neighbors are to one another. Similar to the k_B/k ratio, the value of the clustering coefficient is strongly dependent on the node's degree. Among individuals with $k<20$, females tend to have higher C, but it is not exclusive as there is a large variability in this group. Since the IBD analysis was able to identify many distant relatives, C does not decrease abruptly as the k increases as it would have been otherwise expected. In the $20<k<40$ section, the females and males from KFJ possess a considerably large C implying a strong interconnection among the neighbors of these individuals. The $k>40$ section is dominated by RKF individuals as it was the largest sampled archaeological site. Nonetheless, in this section the C values do not decline considerably neither for females nor for males which still indicates a strong interconnection between neighbors. Since the connections are based on the IBDs, the high C value is not so surprising, as "my relative's relative is my relative" principle predicts connections between neighboring nodes. However, the large k and C together indicate extended biological relatedness between the individuals of RKF that was captured between even more distant relatives too (Supplementary Fig. 46).

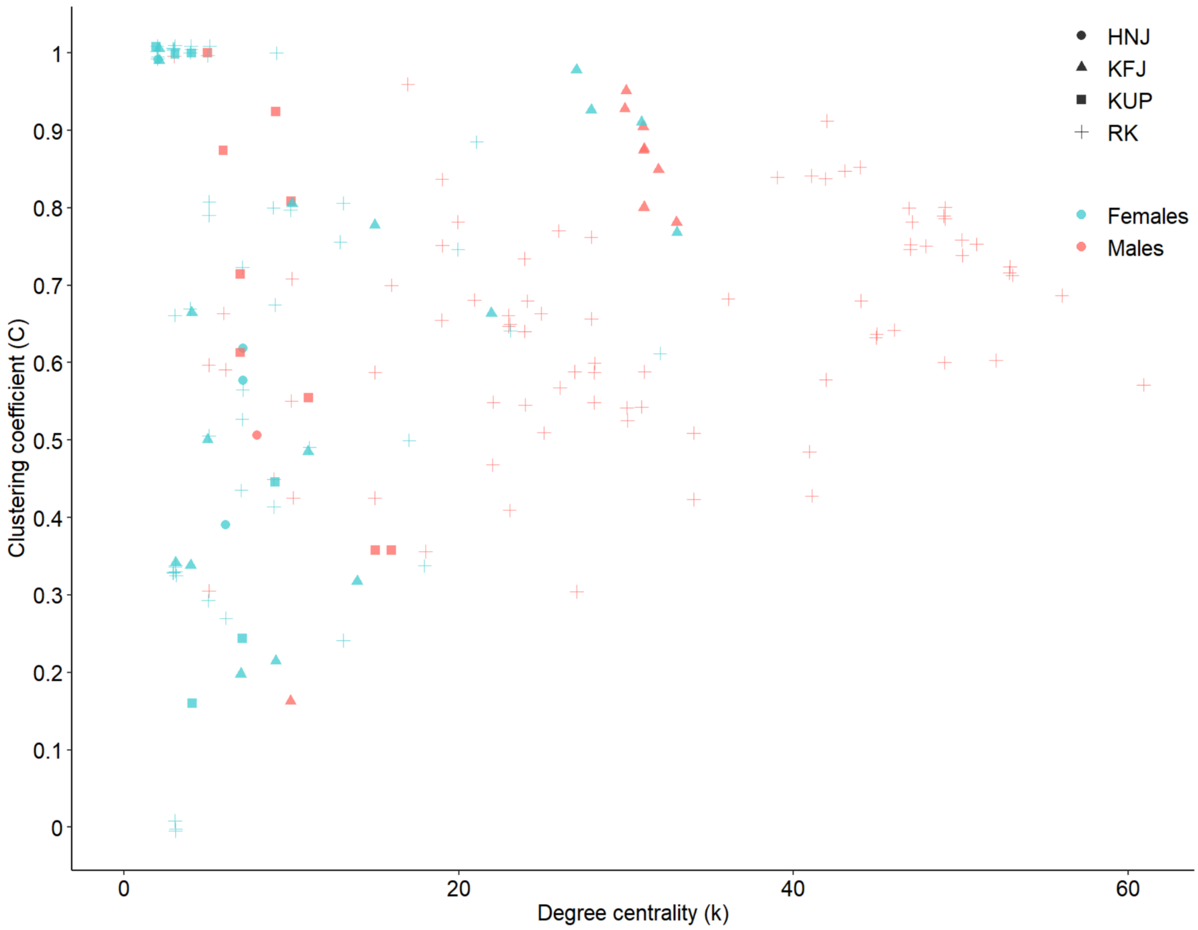

Supplementary Fig. 46 - Clustering coefficient (*C*) and degree centrality (*k*). Individuals are color coded by sex and shown with different symbols representing the four different sites.

A clique is a subset of nodes in an undirected graph network where every pair of vertices are adjacent to one another, meaning that its induced subgraph is complete. The largest clique contains 21 nodes (10.7% of the nodes) thus 210 links (17.3% of the links). The clique is composed of members from the Ped4, Ped5 and 1 unrelated individual from Rákóczifalva. The presence of a large clique could indicate a recent common ancestor, which is consistent with them being part of two connected pedigrees. What is more surprising is that despite all the individuals being connected through IBDs with each other forming a closed circle of interrelatedness, we still didn't find any consanguineous union (i.e. a biologically related male and female having offspring together). The presence of this large clique together with the absence of consanguinity amongst the individuals is yet another strong indication that these individuals knew their biological relatives. The sex ratio within the clique is unequal (21 males, 0 females) which further confirms the patrilineal descent. More distant relatives probably could not be detected by the IBD analysis, as there are first-degree relatives in the family tree who were not included in this clique.

When the entire Avar Period network is considered, the subadults (62 individuals; 35 males, 27 females) were also added to the analysis. In the subadult age group the degree and the strength distribution does not reveal any statistically significant difference between the sexes (p=0.603; p=0.420, respectively). So that the sex-specific differences in the entire network are less pronounced than if only adults were considered. Despite this, the degree distribution between the males and females remained significantly different for the entire network ($p<0.05$ with Kolmogorov-Smirnov test) due to the marked differences among the adults (Supplementary Fig. 47). Similarly, the k_B/k ratio for the entire network showed that females had higher ratios of connections between sites ($p<0.05$ with Welch's t-test) (Supplementary Fig. 48).

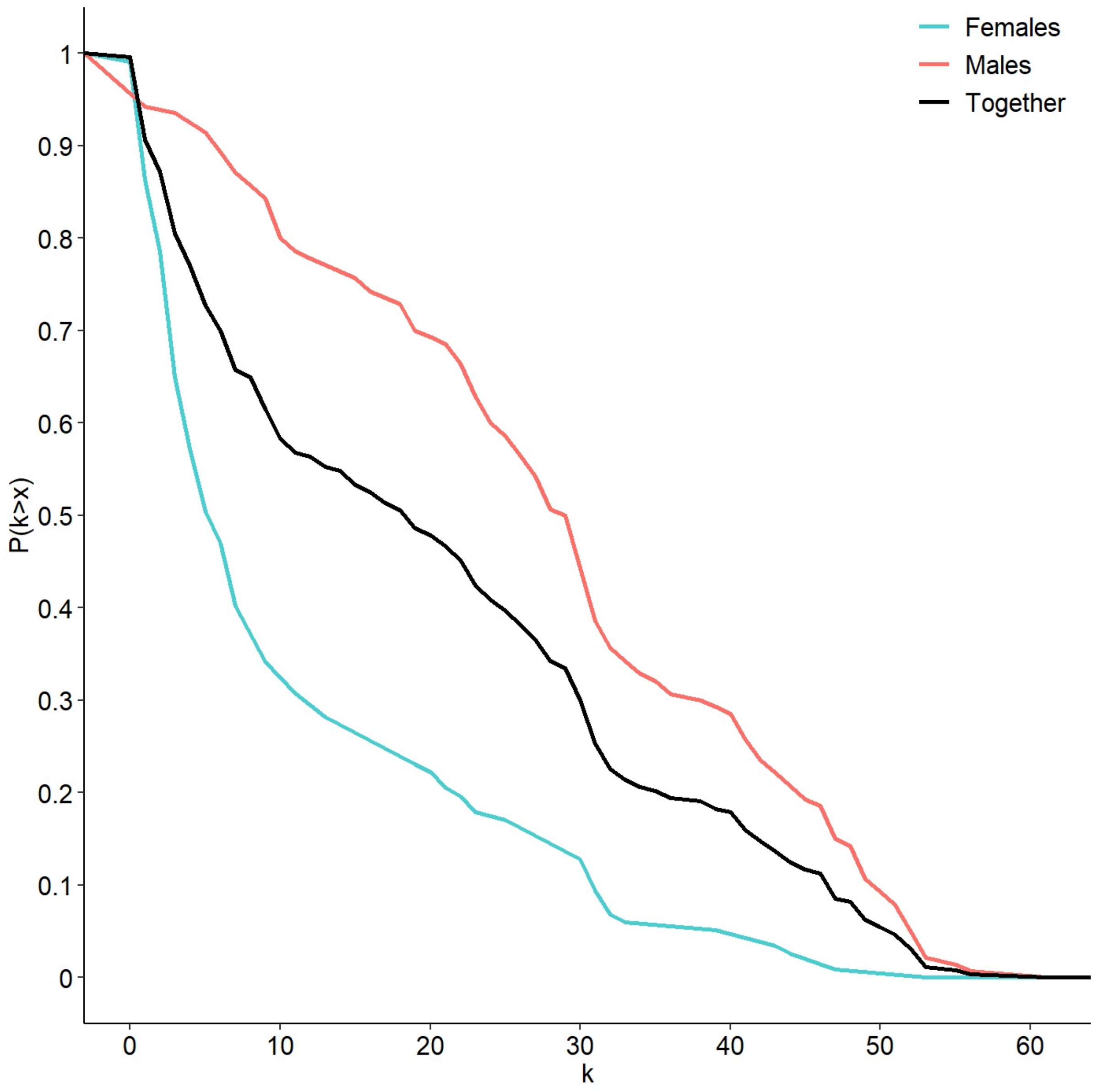

Supplementary Fig. 47 - Whole network (adults and sub-adults) statistics: the degree centrality, *k* (defined as the number of links held by the node) is plotted vs the cumulative density function of the degree's distribution.

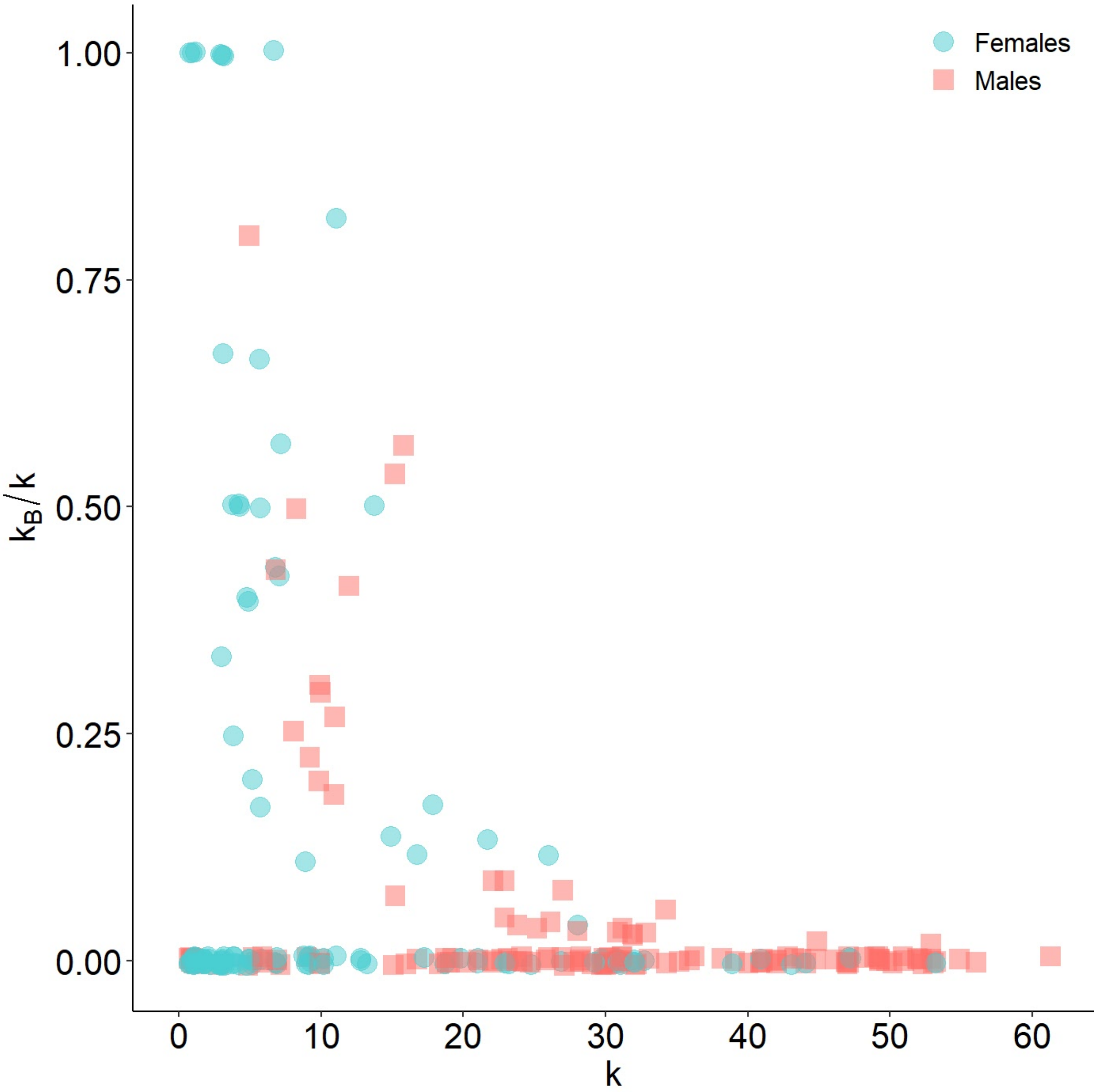

Supplementary Fig. 48 - Whole network (adults and sub-adults) statistics: the total *k* is plotted vs the ratio of *k* calculated between-site edges and total *k* (k_B/k).

Finally, to further test whether our additional conservative filtering of removing pairs of individuals that had only 1 segment in IBD shorter than 16cM (always including only IBDs longer than 12cM) would introduce some biases in the network statistics we calculated (*k*) and (*w*) on an adults only network including all pairs with at least 1 segment > 12cM. We found that including these pairs in the

network did not have any effect on the general trends observed for *k* and *w* nor the k_B/k ratio (Supplementary Fig. 49 and 50)

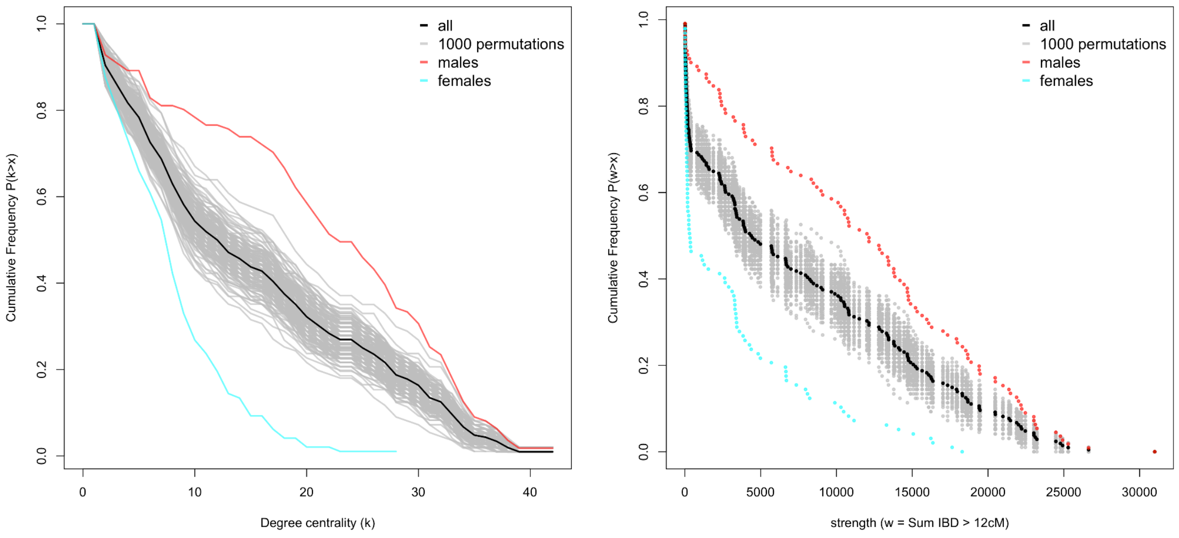

Supplementary Fig. 49 - Statistics including pairs with only 1 IBD segment < 16 cM: the degree centrality *k* (left) and the strength *w* (right) are plotted vs the cumulative density function of their respective distributions.

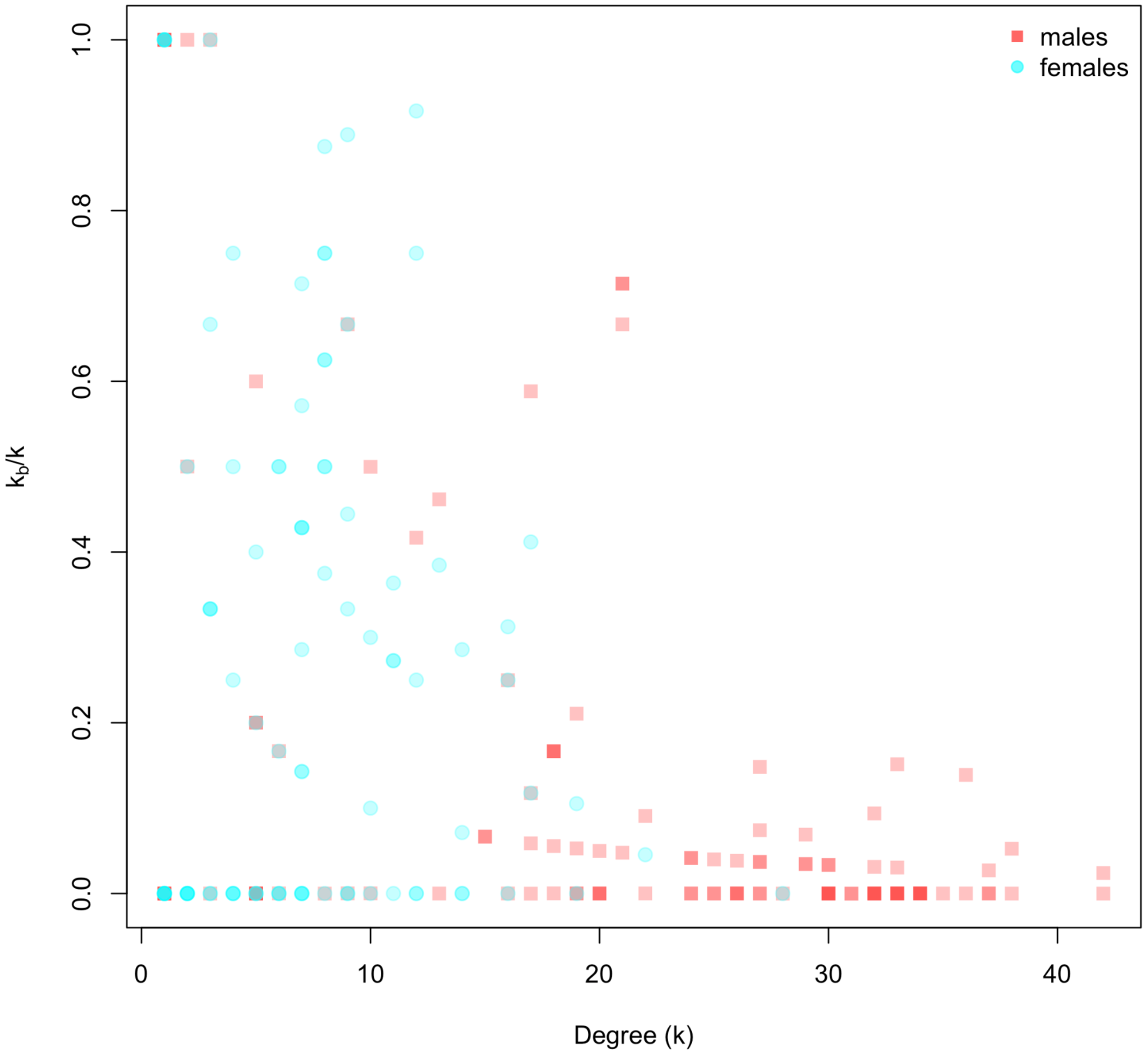

Supplementary Fig. 50 - Statistics including pairs with only 1 IBD segment < 16 cM: the total k is plotted vs the ratio of k calculated between-site edges and total k (k_B/k).

References

1. Pohl, W. *The Avars: A Steppe Empire in Central Europe, 567--822*. (Cornell University Press, 2018).

2. Pohl, W. Ethnonyms and Early Medieval Ethnicity: Methodological Reflections. *The Hungarian Historical Review* **7**, 5–17 (2018).

3. Brather, S. *Ethnische Interpretationen in der frühgeschichtlichen Archäologie*. (De Gruyter,

2004).

4. Geary, P. & Veeramah, K. Mapping European population movement through genomic research. *Mediev. Worlds* **medieval worlds**, 65–78 (2016).
5. Gnecchi-Ruscone, G. A. *et al.* Ancient genomes reveal origin and rapid trans-Eurasian migration of 7 century Avar elites. *Cell* **185**, 1402–1413.e21 (2022).
6. Pohl, W., Di Cosmo, N. & Maas, M. Ethnicity and Empire in the Western Eurasian Steppes. in *Empires and Exchanges in Eurasian Late Antiquity: Rome, China, Iran, and the Steppe, ca. 250–750* 189–205 (Cambridge University Press, 2018).
7. Hinds, K. *Scythians and Sarmatians*. (Marshall Cavendish, 2010).
8. Istvánovits, E. & Kulcsár, V. *Sarmatians History and Archaeology of a Forgotten People*. (RGZM Verlag, 2017).
9. Pohl, W. Nomad Kingdoms of the Eurasian Steppe and the Restructuring of the Danubian Frontier: Sarmatians, Huns, Avars, and Slavs, in *The Oxford Handbook of the Roman World* (ed. Lenski, N.) vol. 8 (Oxford and New York).
10. Heather, P. *The Fall of the Roman Empire: A New History of Rome and the Barbarians*. (Oxford University Press, 2005).
11. Pohl, W. Goths and Huns. in *A Companion to Ethnicity in the Ancient Mediterranean* 555–568 (John Wiley & Sons, Inc, 2014).
12. Steinacher, R. *Rom und die Barbaren: Völker im Alpen- und Donauraum (300-600)*. (Kohlhammer Verlag, 2017).
13. Golden, P. B. Some Notes on the Avars and Rouran. in *The steppe lands and the world beyond them : studies in honor of Victor Spinei on his 70th birthday* (eds. Spinei, V., Curta, F., Maleon, B.-P., Treadgold, W. & Shepard, J.) 43–66 (Editura Universității „Alexandru Ioan Cuza'' din Iași, 2013).
14. Mácsai, V. A Rákóczifalva-Bagi-földek 8A avar temetőjének feldolgozása. (Eötvös Loránd Univ. Budapest, 2012).
15. Schmid, M. M. E. *Das Gräberfeld von Rákóczifalva in Zentralungarn und die Chronologie des spätawarenzeitlichen Fundmaterials*. (Habelt, 2015).

16. Kondé, Z. Avar kori földbe mélyített és földfelszíni épületek Rákóczifalva-Bagi-Földek lelőhelyen. *Archaeol. Ért.* **140**, 71–92 (2015).
17. Vig, L. Avar kori település Rákóczifalva - Bivaly-tó, Bagi-földek lelőhelyen. (Eötvös Loránd Univ. Budapest, 2022).
18. Volk, A. A. & Atkinson, J. A. Infant and child death in the human environment of evolutionary adaptation. *Evol. Hum. Behav.* **34**, 182–192 (2013).
19. Gulyás, B. A kelet-európai sztyeppe és a Kárpát-medence kapcsolata az 5-7. században. A tiszántúli kora avar kori népesség eredete. (Eötvös Loránd Univ. Budapest, 2022).
20. Komar, A. V. Кочевники восточноевропейских степей второй половины VI – первой половины VIII вв. in *Западный Тюркский Каганат. Атлас.* (eds. Dosymbaeva, A. & Zholdasbekov, M.) 673–739 (Astana: Service Press, 2013).
21. Gulyás, B. & Lőrinczy, G. A részleges állatmelléklet egy sajátos változata a Szegvár-oromdűlői kora avar kori temető sírjaiban és annak kelet-európai kapcsolatai. in *Tomka 80. Ünnepi tanulmányok Tomka Péter köszöntésére.* (ed. Nemes, G.) 179–190 (Győr: Rómer Flóris Művészeti és Történeti Múzeum, 2020).
22. Blay, A. & Samu, L. Awarenzeitliche Netzwerke aufgrund archäologischer Funde vom 6. bis zum Ende des 7. Jahrhunderts. in *Grenzüberschreitungen - Reiternomaden in Mitteleuropa, ihre östlichen Wurzeln und Verbindungen* (eds. Meller, H. & Daim, F.) 293–314 (14th Central German Archaeological Conference, 7-9 October 2021, 2022).
23. Rácz, Z. *Die Goldschmiedegräber der Awarenzeit.* vol. 116 (Monographien des Römisch-Germanischen, 2014).
24. Garam, É. *Funde byzantinischer Herkunft in der Awarenzeit vom Ende des 6. bis zum Ende des 7. Jahrhunderts*. (Monumenta Avarorum Archaeologica 5, 2001).
25. Szenthe, G. Antique meaning — Avar significance complex iconographic schemes on early medieval small objects. *Acta Archaeologica Academiae Scientiarum Hungaricae* **64**, 139–172 (2013).
26. Masek, Z. A Közép-Tisza-vidék településtörténete a Kr. u. 4–6. században Rákóczifalva-Bagi-földek 5–8–8A. lelőhely értékelése alapján. (Eötvös Loránd University Budapest, 2018).

27. Masek Z. Settlement History of the Middle Tisza Region in the 4th–6th centuries AD. *DissArch* 597–619 (2018).

28. Rácz, Z. & Szenthe, G. Avar temető Hajdúnánás határában. *Communicationes Archaeologicae Hungariae* vol. 2009 309–335 Preprint at https://doi.org/10.54640/cah.2009.309 (2009).

29. Hajdu, T., Guba, Z. & Pap, I. A hajdúnánási avar temető embertani leletei. *Communicationes Archaeologicae Hungariae* vol. 2009 339–358 Preprint at https://doi.org/10.54640/cah.2009.339 (2009).

30. Siklósi, Z. *Tiszavasvári-Kashalom-dűlőben és Hajdúnánás-Fürj-halom-járáson feltárt avar sírok radiokarbon keltezése (Radiocarbon dating of Avar graves excavated in Tiszavasvári-Kashalom-dűlő and Hajdúnánás-Fürj-halom-járás)*. 229–236 (A Nyíregyházi Jósa András Múzeum Évkönyve 56, 2014).

31. Hampel, J. Nemzeti Múzeumi régiségtár gyarapodásam. *Archaeologiai Értesítő* **14**, 184–187 (1894).

32. Tóth, H. E. Korai avar vezetõréteg családi temetõje a kunbábonyi kagán szállásterületén. *Múzeumi Kutatások Bács-Kiskun Megyében* 10–20 (1984).

33. Balogh, C. A Duna-Tisza köze avar kori betelepülésének problémái. (Eötvös Loránd Univ. Budapest, 2013).

34. Marcsik, A. A kunpeszéri avar kori széria humán csontanyagának feldolgozása. in *„...In terra quondam Avarorum." Ünnepi tanulmányok H. Tóth Elvira 80. születésnapjára.* (eds. Somogyvári, Á. & Székely, G.) 175–190 (Archaeologia Cumanica 2, 2009).

35. Csáky, V. *et al.* Genetic insights into the social organisation of the Avar period elite in the 7th century AD Carpathian Basin. *Sci. Rep.* **10**, 948 (2020).

36. Lezsák, M. G. *Avarok a Herke-tónál. A Kunszállás-fülöpjakabi avar temető története*. (Antológia Kiadó, 2008).

37. Mende, B. G. Metodikai tanulmány Kunszállás-Fülöpjakab avarkori temetőjének embertani anyagán. (University of Szeged, 1995).

38. Krader, L. Principles and Structures in the Organization of the Asiatic Steppe-Pastoralists. *Southwest. J. Anthropol.* **11**, 67–92 (1955).

39. Amorim, C. E. G. *et al.* Understanding 6th-century barbarian social organization and migration through paleogenomics. *Nat. Commun.* **9**, 3547 (2018).
40. Schlee, G. Omaha Terminologies: Global Distribution Patterns and How They May Have Come About. *Cross Cult. Res.* **51**, 117–141 (2017).
41. Rácz, P., Passmore, S. & Jordan, F. M. Social Practice and Shared History, Not Social Scale, Structure Cross-Cultural Complexity in Kinship Systems. *Top. Cogn. Sci.* **12**, 744–765 (2020).
42. Taşbaş, E. The Turkic kinship system. *Acta Orient.* **72**, 245–258 (2019).
43. Kara, D. S. The Formation of Modern Turkic 'Ethnic' Groups in Central and Inner Asia. *The Hungarian Historical Review* **7**, 98–110 (2018).
44. Kradin, N. N. From Tribal Confederation to Empire: The Evolution of the Rouran Society. *Acta Orientalia Academiae Scientiarum Hungaricae* vol. 58 149–169 Preprint at (2005).
45. Onon, U. *The Secret History of the Mongols*. (Routledge, 2005).
46. Khazanov, A. M. *Nomads and the Outside World*. (The University of Wisconsin Press, 1994).
47. Holmgren, J. Imperial Marriage in the Native Chinese and Non-Han State, Han to Ming. in (Oxford University Press, 1991).
48. Szenthe, G. Crisis or Innovation? A Technology-Inspired Narrative of Social Dynamics in the Carpathian Basin during the Eighth Century. in *Zwischen Byzanz und der Steppe. Archäologische und historische Studien. Festschrift für Csanád Bálint zum 70. Geburtstag* (eds. Bollók, Á., Csiky, G. & Vida, T.) 351–370 (Institute of Archeology, Hungarian Academy of Sciences, 2016).
49. Blay, A. & Samu, L. Térbeli átrendeződés a 7. századi Kárpát-medencében/Regional shifts in the Carpathian Basin during the seventh century. in *Hatalmi központok az Avar Kaganátusban/Power centres of the Avar Khaganate* (eds. Balogh, C., Szentpéteri, J. & Wicker, E.) 263–332 (2019).
50. Bálint, C. Gyula László's theory of the 'two-time conquest of the Magyars' and the archaeology of the Avars. *Acta Archaeologica Academiae Scientiarum Hungaricae* **74**, 121–141 (2023).
51. Szenthe, G. The 'Late Avar' Reform and the 'Long Eighth Century': A Tale of the Hesitation between Structural Transformation and the Persistent Nomadic Traditions (7th to 9th Century

AD). *Acta Archaeologica Academiae Scientiarum Hungaricae* **70**, 215–250 (2019).

52. Vida, T. The process of the settlement of the Carpathian Basin by the Avars and their configuration of power. in *The Huns to the Turks – Mounted warriors in Europe and Central Asia* (ed. Daim, F., Meller, H., Pohl, W.) 171–189 (Tagungen des Landesmuseums für Vorgeschichte Halle, 2021).
53. Harney, É., Patterson, N., Reich, D. & Wakeley, J. Assessing the performance of qpAdm: a statistical tool for studying population admixture. *Genetics* **217**, (2021).
54. Yüncü, E. *et al.* False discovery rates of qpAdm-based screens for genetic admixture. *bioRxiv* 2023.04.25.538339 (2023) doi:10.1101/2023.04.25.538339.
55. Vida, T. Neue archäologische und archäogenetische Forschungen zur awarischen Elite des 7. Jahrhunderts im Karpatenbecken. in *Crossing boundaries - Mounted nomads in Central Europe, their eastern roots and connections.* (ed. Daim, F., Meller, H.) 260–275 (Tagungen des Landesmuseums für Vorgeschichte Halle, 2022).
56. Csiky, G. Innovations in the weaponry of early medieval mounted nomads. in *Crossing boundaries - Mounted nomads in Central Europe, their eastern roots and connections.* (ed. Daim, F., Meller, H.) 33–44 (Tagungen des Landesmuseums für Vorgeschichte Halle, 2022).
57. Lőrinczy, G. Frühawarenzeitliche Bestattungßitten im Gebiet der Großen Ungarischen Tiefebene östlich der Theiß. Archäologische Angaben und Bemerkungen zur Geschichte der Region im 6. und 7. Jahrhundert. *Acta Archaeologica Academiae Scientiarum Hungaricae* vol. 68 137–169 Preprint at (2017).
58. Gulyás, B. ONE PEOPLE, TWO REGIONS? Thoughts on the Early Avar Period System of Relationships in Eastern Europe beyond the Tisza River. *Hungarian Archaeology* **E-journal**, (2016).
59. Stark, S. A 'Rouran Perspective' on the Northern Chinese Frontier during the Northern Wei Period. Some thoughts on the Yihe-nur tombs. in *From the Huns to the Turks – Mounted warriors in Europe and Central Asia.* (ed. Daim, F., Meller, H., Pohl, W.) 59–87 (Tagungen des Landesmuseums für Vorgeschichte Halle, 2021).
60. Kovács, P. & Wolff, C. Notes on the 'Pannonian' foederati. in *Les auxiliaires de l'armée*

romaine. Des alliés aux fédérés. Actes du sixième Congrès de Lyon (23–25 octobre 2014) (ed. Wolff, C.) 575–601 (Diffusion Librairie De Boccard, 2016).

61. Istvánovits, E. & Kulcsár, V. The 'argumentum ex silentio': a possible new approach in the research of the Hun period. in *Attila's Europe? Structural transformation and strategies of success in the European Hun period* (eds. Rácz, Z. & Szenthe, G.) 435–447 (Benedek Varga; Dávid Bartus, 2021).
62. Szenthe, G. Social power, identity and the ritual deposits in "Attila's Europe". in *Attila's Europe? Structural transformation and strategies of success in the European period* (eds. Rácz, Z. & Szenthe, G.) 563–579 (Benedek Varga; Dávid Bartus, 2021).
63. Maróti, Z. *et al.* The genetic origin of Huns, Avars, and conquering Hungarians. *Curr. Biol.* **32**, 2858–2870.e7 (2022).
64. Curta, F. Ethnicity in the Steppe Lands of the Northern Black Sea Region During The Early Byzantine Times. *Archaeol. Bulgarica* **23**, 33–70 (2019).
65. Jeong, C. *et al.* A Dynamic 6,000-Year Genetic History of Eurasia's Eastern Steppe. *Cell* **183**, 890–904.e29 (2020).
66. Popli, D., Peyrégne, S. & Peter, B. M. KIN: a method to infer relatedness from low-coverage ancient DNA. *Genome Biol.* **24**, 10 (2023).
67. Monroy Kuhn, J. M., Jakobsson, M. & Günther, T. Estimating genetic kin relationships in prehistoric populations. *PLoS One* **13**, e0195491 (2018).
68. Jeong, C. *et al.* Bronze Age population dynamics and the rise of dairy pastoralism on the eastern Eurasian steppe. *Proc. Natl. Acad. Sci. U. S. A.* **115**, E11248–E11255 (2018).
69. Kennett, D. J. *et al.* Archaeogenomic evidence reveals prehistoric matrilineal dynasty. *Nat. Commun.* **8**, 14115 (2017).
70. Depaermentier, M. L. C., Kempf, M., Bánffy, E. & Alt, K. W. Modelling a scale-based strontium isotope baseline for Hungary. *Journal of Archaeological Science* vol. 135 105489 Preprint at https://doi.org/10.1016/j.jas.2021.105489 (2021).
71. Depaermentier, M. L. C., Kempf, M., Bánffy, E. & Alt, K. W. Tracing mobility patterns through the 6th-5th millennia BC in the Carpathian Basin with strontium and oxygen stable isotope

analyses. *PLoS One* **15**, e0242745 (2020).

72. Knipper, C. *et al.* Coalescing traditions-Coalescing people: Community formation in Pannonia after the decline of the Roman Empire. *PLoS One* **15**, e0231760 (2020).

73. Alt, K. W. *et al.* Lombards on the move--an integrative study of the migration period cemetery at Szólád, Hungary. *PLoS One* **9**, e110793 (2014).

74. Klinken, G. J. Bone Collagen Quality Indicators for Palaeodietary and Radiocarbon Measurements. *J. Archaeol. Sci.* **26**, 687–695 (1999).

75. Kohn, M. J. Carbon isotope compositions of terrestrial C3 plants as indicators of (paleo)ecology and (paleo)climate. *Proc. Natl. Acad. Sci. U. S. A.* **107**, 19691–19695 (2010).

76. Diefendorf, A. F., Mueller, K. E., Wing, S. L., Koch, P. L. & Freeman, K. H. Global patterns in leaf 13C discrimination and implications for studies of past and future climate. *Proc. Natl. Acad. Sci. U. S. A.* **107**, 5738–5743 (2010).

77. Giblin, J. I. & Yerkes, R. W. Diet, dispersal and social differentiation during the Copper Age in eastern Hungary. *Antiquity* **90**, 81–94 (2016).

78. Cernusak, L. A. *et al.* Why are non-photosynthetic tissues generally C enriched compared with leaves in C plants? Review and synthesis of current hypotheses. *Funct. Plant Biol.* **36**, 199–213 (2009).

79. Christensen, B. T., Jensen, J. L., Dong, Y. & Bogaard, A. Manure for millet: Grain δ15N values as indicators of prehistoric cropping intensity of Panicum miliaceum and Setaria italica. *J. Archaeol. Sci.* **139**, 105554 (2022).

80. Dong, Y. *et al.* The potential of stable carbon and nitrogen isotope analysis of foxtail and broomcorn millets for investigating ancient farming systems. *Front. Plant Sci.* **13**, 1018312 (2022).

81. Gyulai, F. Archaeobotanical overview of rye (Secale cereale L.) in the Carpathian-basin I. from the beginning until the roman age. *Columella: Journal of Agricultural and Environmental Sciences* (2014).

82. Faragó, N. *et al.* Dietary and cultural differences between neighbouring communities: A case study on the early medieval Carpathian Basin (Avar and post-Avar period, 7th–9th/10th centuries

AD). *Journal of Archaeological Science: Reports* **42**, 103361 (2022).

83. Noche-Dowdy, L. D. Multi-Isotope Analysis to Reconstruct Dietary and Migration Patterns of an Avar Population from Sajópetri, Hungary, AD 568-895. (University of South Florida, 2015).

84. Herold, M. Sex differences in mortality in Lower Austria and Vienna in the Early Medieval period: an investigation and evaluation of possible contributing factors. (University of Vienna, 2008).

85. Vidal-Ronchas, R., Rajić Šikanjić, P., Premužić, Z., Rapan Papeša, A. & Lightfoot, E. Diet, sex, and social status in the Late Avar period: stable isotope investigations at Nuštar cemetery, Croatia. *Archaeol. Anthropol. Sci.* **11**, 1727–1737 (2019).

86. Katzenberg, M. A. Stable isotope analysis: A tool for studying past diet, demography, and life history. in *Biological Anthropology of the Human Skeleton* 411–441 (John Wiley & Sons, Inc., 2008).

87. Lee-Thorp, J. A. On isotopes and old bones. *Archaeometry* **50**, 925–950 (2008).

88. Hedges, R. E. M. & Reynard, L. M. Nitrogen isotopes and the trophic level of humans in archaeology. *J. Archaeol. Sci.* **34**, 1240–1251 (2007).

89. Fraser, R. A. *et al.* Manuring and stable nitrogen isotope ratios in cereals and pulses: towards a new archaeobotanical approach to the inference of land use and dietary practices. *J. Archaeol. Sci.* **38**, 2790–2804 (2011).

90. Bogaard, A., Heaton, T. H. E., Poulton, P. & Merbach, I. The impact of manuring on nitrogen isotope ratios in cereals: archaeological implications for reconstruction of diet and crop management practices. *J. Archaeol. Sci.* **34**, 335–343 (2007).

91. Robson, H. K. New ichthyoarchaeological data from the Mesolithic lakeshore settlement site of Friesack IV. in *Subsistenz und Umwelt der Feuchtbodenstation Friesack 4 im Havelland: Ergebnisse der naturwissenschaftlichen Untersuchungen* 160–177 (Brandenburgisches Landesamt für Denkmalpflege, 2016).

92. Häberle, S. *et al.* Carbon and nitrogen isotopic ratios in archaeological and modern Swiss fish as possible markers for diachronic anthropogenic activity in freshwater ecosystems. *Journal of Archaeological Science: Reports* **10**, 411–423 (2016).

93. Fernandes, R., Grootes, P., Nadeau, M.-J. & Nehlich, O. Quantitative diet reconstruction of a Neolithic population using a Bayesian mixing model (FRUITS): The case study of Ostorf (Germany). *Am. J. Phys. Anthropol.* **158**, 325–340 (2015).

94. Fernandes, R., Millard, A. R., Brabec, M., Nadeau, M.-J. & Grootes, P. Food reconstruction using isotopic transferred signals (FRUITS): a Bayesian model for diet reconstruction. *PLoS One* **9**, e87436 (2014).

95. Hoekman-Sites, H. A. & Giblin, J. I. Prehistoric animal use on the Great Hungarian Plain: A synthesis of isotope and residue analyses from the Neolithic and Copper Age. *Journal of Anthropological Archaeology* **31**, 515–527 (2012).

96. Reynard, L. M. & Tuross, N. The known, the unknown and the unknowable: weaning times from archaeological bones using nitrogen isotope ratios. *J. Archaeol. Sci.* **53**, 618–625 (2015).

97. Nitsch, E. K., Humphrey, L. T. & Hedges, R. E. M. Using stable isotope analysis to examine the effect of economic change on breastfeeding practices in Spitalfields, London, UK. *Am. J. Phys. Anthropol.* **146**, 619–628 (2011).

98. Reitsema, L. J. & Muir, A. B. Growth velocity and weaning δ(15)N 'Dips' during ontogeny in Macaca mulatta. *Am. J. Phys. Anthropol.* **157**, 347–357 (2015).

99. Waters-Rist, A. L. & Katzenberg, M. A. The effect of growth on stable nitrogen isotope ratios in subadult bone collagen. *Int. J. Osteoarchaeol.* (2009) doi:10.1002/oa.1017.

100. Hammer, Ø., Harper, D. A. T., Ryan, P. D. & Others. PAST: Paleontological statistics software package for education and data analysis. *Palaeontol. Electronica* **4**, 9 (2001).

101. Knipper, C. *et al.* Superior in Life—Superior in Death: Dietary Distinction of Central European Prehistoric and Medieval Elites. *Curr. Anthropol.* **56**, 579–589 (2015).

102. Massy, K., Friedrich, R., Mittnik, A. & Stockhammer, P. W. Pedigree-based Bayesian modelling of radiocarbon dates. *PLoS One* **17**, e0270374 (2022).

103. Ramsey, C. B. Radiocarbon Calibration and Analysis of Stratigraphy: The OxCal Program. *Radiocarbon* vol. 37 425–430 Preprint at https://doi.org/10.1017/s0033822200030903 (1995).

104. Barta, P. & Štolc, S. HBCO Correction: Its Impact on Archaeological Absolute Dating. *Radiocarbon* vol. 49 465–472 Preprint at https://doi.org/10.1017/s0033822200042399 (2007).

101. Bálint, Cs. Gyula László's theory of the "two-time conquest of the Magyars" and the archaeology of the Avars. Acta Archaeologica Academiae Scientiarum Hungaricae 74, issue 1 (2023) 121-141 https://doi.org/10.1556/072.2023.00009

102. Vida, T. The process of the settlement of the Carpathian Basin by the Avars and their configuration of power. in From the Huns to the Turks – Mounted warriors in Europe and Central Asia. (ed. Daim, F., Meller, H., Pohl, W.) 171–189 (Tagungen des Landesmuseums für Vorgeschichte Halle, 2021)

103. Szenthe, G. The »Late Avar reform« and the »long eighth century«: A tale of the hesitation between structural transformation and the persistent nomadic traditions (7th to 9th century AD). Acta Arch. Acad. Scien. Hungaricae 70, 2019, 215–250.

104. Lechner Nonprofit Kft. (2023).

105. Photo of Kunpeszér sword: Katona József Museum (Kecskemét, Hungary); photo of Rákóczifalva excavation: Sándor Hegedűs.

106. Made with Natural Earth. Free vector and raster map data @ naturalearthdata.com.
