# Supplementary material for: Network of large pedigrees reveals social practices of Avar communities
Source: Nature. 2024 Apr 24;629(8011):376–83. doi: 10.1038/s41586-024-07312-4 (PMC11078744; doi:10.1038/s41586-024-07312-4)
Supplement: Supplementary file 2 — Reporting Summary [file 41586_2024_7312_MOESM2_ESM.pdf]

Corresponding author(s): Guido Alberto Gnecchi-Ruscone

Last updated by author(s): 23/01/24

Reporting Summary

Nature Portfolio wishes to improve the reproducibility of the work that we publish. This form provides structure for consistency and transparency in reporting. For further information on Nature Portfolio policies, see our Editorial Policies and the Editorial Policy Checklist.

Statistics

For all statistical analyses, confirm that the following items are present in the figure legend, table legend, main text, or Methods section.

n/a	Confirmed	
☐	☒	The exact sample size (*n*) for each experimental group/condition, given as a discrete number and unit of measurement
☐	☒	A statement on whether measurements were taken from distinct samples or whether the same sample was measured repeatedly
☐	☒	The statistical test(s) used AND whether they are one- or two-sided<br>*Only common tests should be described solely by name; describe more complex techniques in the Methods section.*
☒	☐	A description of all covariates tested
☒	☐	A description of any assumptions or corrections, such as tests of normality and adjustment for multiple comparisons
☐	☒	A full description of the statistical parameters including central tendency (e.g. means) or other basic estimates (e.g. regression coefficient) AND variation (e.g. standard deviation) or associated estimates of uncertainty (e.g. confidence intervals)
☐	☒	For null hypothesis testing, the test statistic (e.g. *F*, *t*, *r*) with confidence intervals, effect sizes, degrees of freedom and *P* value noted<br>*Give P values as exact values whenever suitable.*
☐	☒	For Bayesian analysis, information on the choice of priors and Markov chain Monte Carlo settings
☒	☐	For hierarchical and complex designs, identification of the appropriate level for tests and full reporting of outcomes
☒	☐	Estimates of effect sizes (e.g. Cohen's *d*, Pearson's *r*), indicating how they were calculated

Our web collection on statistics for biologists contains articles on many of the points above.

Software and code

Policy information about availability of computer code

Data collection	No specific software was used for sample collection. Genotype data were generated from sequencing reads. All software used in this study is listed below.
Data analysis	The following freely available software were used for data analyses, the corresponding citations are provided in the Methods section: nf-core/eager v2.3.2 (https://nf-co.re/eager); AdapterRemoval (v2.3.1), BWA (v0.7.17), MarkDuplicates v2.22.9 (https://github.com/broadinstitute/picard) , mapDamage (v2.0), BamUtil (v1.0.13), ANGSD (v0.910), Schmutzi (v0.7.12), pileupCaller (https://github.com/stschiff/sequenceTools), samtools (v1.9), HaploGrep2 (v2.4.0), smartpca (v16000; EIGENSOFT v6.0.1), qpWave/qpAdm (v1520), yHaplo (v11.249) & Y-LineageTracker using the ISOGG SNP index (v.11.349 and v15.73), ATLAS (v0.9), R(v 4.05), DATES (v 753), AncIBD v0.5 (https://pypi.org/project/ancIBD/), hapROH v0.64 (https://pypi.org/project/hapROH/), KIN v3.1.3 (https://github.com/DivyaratanPopli/Kinship_Inference), BREADR (https://github.com/jonotuke/BREADR), Cytoscape (v3.9.1), Oxcal (v.4.4.4).

For manuscripts utilizing custom algorithms or software that are central to the research but not yet described in published literature, software must be made available to editors and reviewers. We strongly encourage code deposition in a community repository (e.g. GitHub). See the Nature Portfolio guidelines for submitting code & software for further information.

Data

Policy information about availability of data

All manuscripts must include a data availability statement. This statement should provide the following information, where applicable:

- Accession codes, unique identifiers, or web links for publicly available datasets
- A description of any restrictions on data availability
- For clinical datasets or third party data, please ensure that the statement adheres to our policy

The newly produced sequence data is deposited in the European Nucleotide Archive (ENA) with the following accession number: PRJEB72021. The new haploid genotype data is available through the Poseidon framework (https://github.com/poseidon-framework/community-archive) under https://github.com/poseidon-framework/community-archive/tree/master/2024_GnecchiRuscone_HungaryAvarPedigrees.

Field-specific reporting

Please select the one below that is the best fit for your research. If you are not sure, read the appropriate sections before making your selection.

☒ Life sciences ☐ Behavioural & social sciences ☐ Ecological, evolutionary & environmental sciences

For a reference copy of the document with all sections, see nature.com/documents/nr-reporting-summary-flat.pdf

Life sciences study design

All studies must disclose on these points even when the disclosure is negative.

Sample size	We did not rely on statistical methods to predetermine sample sizes. Sample sizes for ancient populations depended solely on the availability of archaeological material and on ancient DNA preservation. We applied a "whole cemetery sampling" approach, meaning that we exhaustively sampled all the individuals excavated from the four cemeteries under investigation
Data exclusions	Data from specimens that showed insufficient levels of ancient DNA content or high levels of DNA contamination were excluded from further analyses.
Replication	We studied unique entities (past and present populations) and did not perform experiments or study various treatments, so replication is not applicable. But we note that samples from the same population carry similar genetic signatures. Moreover, genome-wide data allows for the analysis of multiple realisations of the sample history, by studying hundreds of thousands of SNP sites.
Randomization	We studied unique entities (past and present populations) and did not perform experiments or study various treatments, so randomization is not applicable.
Blinding	We studied unique entities (past and present populations) and did not perform experiments or study various treatments, so blinding is not applicable.

Reporting for specific materials, systems and methods

We require information from authors about some types of materials, experimental systems and methods used in many studies. Here, indicate whether each material, system or method listed is relevant to your study. If you are not sure if a list item applies to your research, read the appropriate section before selecting a response.

Materials & experimental systems

n/a	Involved in the study
☒	☐ Antibodies
☒	☐ Eukaryotic cell lines
☐	☒ Palaeontology and archaeology
☒	☐ Animals and other organisms
☒	☐ Human research participants
☒	☐ Clinical data
☒	☐ Dual use research of concern

Methods

n/a	Involved in the study
☒	☐ ChIP-seq
☒	☐ Flow cytometry
☒	☐ MRI-based neuroimaging

Palaeontology and Archaeology

Specimen provenance	The Rákóczifalva (RK) cemetery was excavated in 2005-2006 by the Institute of Archaeological Sciences of the Eötvös Loránd University (Katalin Sebők, Gábor Szabó, Katalin Kovács, and Gábor Váczi). The Hajdúnánás-Fürj-halom-járás site (M3 motorway Site 41A) was a rescue excavation in 2005 led by Gábor Szabó from the Institute of Archaeological Sciences of the Eötvös Loránd University. The cemetery of Kunszállás-Fülöpjakab was excavated by Elvira H. Tóth between 1967 and 1979. The site of Kunpeszér,

Felsőpeszéri út (Bács-Kiskun county, Hungary) was retrieve during a rescue excavation, between 1982 and 1984 conducted by Elvira H. Tóth from the Katona József Museum (Kecskemét). Both KFJ and KUP skeletal specimens are conserved at the Department of Biological Anthropology, University of Szeged (Hungary).

Specimen deposition	Specimens were returned to the owning institutions after laboratory analyses. Left over bone powder samples from the specimens are conserved at the Institute of Archaeogenomics, Research Centre for the Humanities, Eötvös Loránd Research Network, Budapest (Hungary).
Dating methods	New AMS 14C dates were obtained from ultra-filtrated collagen. Collagen extraction and 14C measurements were carried out at the Curt-Engelhorn-Center Archaeometry gGmbH (CECA), Manheim, Germany.

☒ Tick this box to confirm that the raw and calibrated dates are available in the paper or in Supplementary Information.

Ethics oversight	No ethical oversight was required strictly. However, we confirm that all analyses followed established ethical guidelines for archaeogenetic research, as detailed in Wagner et al., AJHG, 2020 and Alpaslan-Roodenberg, Nature, 2021. Ester Banfy, the Ethics Advisor of the ERC funded project HistoGenes (grant agreement number 856453 ERC-2019-SyG), approved the study

Note that full information on the approval of the study protocol must also be provided in the manuscript.
